# Substituted Aminoacetamides as Novel Leads for Malaria Treatment

**DOI:** 10.1002/cmdc.201900329

**Published:** 2019-07-03

**Authors:** Neil R. Norcross, Caroline Wilson, Beatriz Baragaña, Irene Hallyburton, Maria Osuna‐Cabello, Suzanne Norval, Jennifer Riley, Daniel Fletcher, Robert Sinden, Michael Delves, Andrea Ruecker, Sandra Duffy, Stephan Meister, Yevgeniya Antonova‐Koch, Benigno Crespo, Cristina de Cózar, Laura M. Sanz, Francisco Javier Gamo, Vicky M. Avery, Julie A. Frearson, David W. Gray, Alan H. Fairlamb, Elizabeth A. Winzeler, David Waterson, Simon F. Campbell, Paul A. Willis, Kevin D. Read, Ian H. Gilbert

**Affiliations:** ^1^ Drug Discovery Unit Division of Biological Chemistry and Drug Discovery School of Life Sciences University of Dundee Dundee DD1 5EH UK; ^2^ Imperial College South Kensington London SW7 2AZ UK; ^3^ Discovery Biology Griffith Institute for Drug Discovery Griffith University Nathan Queensland 4111 Australia; ^4^ Department of Pediatrics University of California San Diego School of Medicine 9500 Gilman Drive 0741 La Jolla CA 92093 USA; ^5^ GlaxoSmithKline, Diseases of the Developing World – Tres Cantos Medicines Development Campus c/ Severo Ochoa 2, Tres Cantos 28760 Madrid Spain; ^6^ Medicines for Malaria Venture International Centre, Cointrin, Entrance G, 3rd Floor Route de Pré-Bois 20, PO Box 1826 Geneva 1215 Switzerland

**Keywords:** aminoacetamides, antimalarial agents, hit optimization, malaria, *Plasmodium falciparum*

## Abstract

Herein we describe the optimization of a phenotypic hit against *Plasmodium falciparum* based on an aminoacetamide scaffold. This led to *N*‐(3‐chloro‐4‐fluorophenyl)‐2‐methyl‐2‐{[4‐methyl‐3‐(morpholinosulfonyl)phenyl]amino}propanamide (compound **28**) with low‐nanomolar activity against the intraerythrocytic stages of the malaria parasite, and which was found to be inactive in a mammalian cell counter‐screen up to 25 μm. Inhibition of gametes in the dual gamete activation assay suggests that this family of compounds may also have transmission blocking capabilities. Whilst we were unable to optimize the aqueous solubility and microsomal stability to a point at which the aminoacetamides would be suitable for in vivo pharmacokinetic and efficacy studies, compound **28** displayed excellent antimalarial potency and selectivity; it could therefore serve as a suitable chemical tool for drug target identification.

## Graphical abstract

**New roads to novel chemotypes**: Compound selection from the TCAMS library (GSK), followed by iterative rounds of inhibitor design and synthesis afforded a single‐digit nanomolar inhibitor of *P. falciparum* (3D7), based on an aminoacetamide core. Compound **28** is a potent antimalarial and displayed excellent selectivity in a mammalian counter‐screen. This compound could be used as a suitable chemical tool for drug target identification or as a lead compound for further optimization.

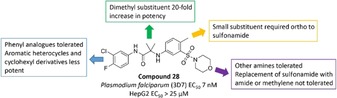

## Introduction

Malaria is a devastating parasitic disease, which is endemic in many parts of the developing world. Human malaria is caused by five species of *Plasmodium*, of which, *P. falciparum* causes the greatest number of deaths, affecting many parts of Africa. *P. vivax* causes severe morbidity and affects mainly Latin America and South East Asia.^1^ In 2017, there were 219 million reported cases of malaria, and >400 000 deaths, many of which were in children under the age of five years.^2^ The malaria parasite is transmitted to humans through the bite of an infected female anopheles mosquito when taking a blood meal. Following infection, malaria parasites in the form of sporozoites, migrate from the skin to the liver, where they invade and develop into merozoites. When merozoites outgrow liver cells, they are released into the blood stream, invading red blood cells, where they continue to grow and develop, repeating the cycle in the erythrocytes. During the intraerythrocytic stages of infection, some parasites differentiate into the sexual form (gametocytes), while others remain as the asexual form, progressing through multiple stages of development within the blood cells. When the parasites are mature, infected red blood cells burst, releasing parasites into the bloodstream, causing the febrile symptoms associated with malaria. Asexual merozoites are then free to invade other red blood cells, whereas gametocytes may be taken up through the bite of a feeding mosquito and transmitted to another human, perpetuating the malaria parasite life cycle.^3^ Current front‐line therapies for the treatment of malaria are failing due to the increasing development of drug resistance and new antimalarial treatments are urgently needed.^4^ Furthermore, novel therapies that target the malaria parasite in all life‐cycle stages are required, to both treat and prevent the spread of malaria and to help in the process of eliminating this disease.^5^

## Results and Discussion

### Project initiation

To develop potential new antimalarials, we focused our attention on the publicly available results of a high‐throughput screening (HTS) campaign of nearly two million compounds carried out by GlaxoSmithKline (GSK), to identify new chemical matter with antimalarial activity.^6^ The screening campaign identified >13 500 compounds malaria blood‐stage actives (Tres Cantos Antimalarial Set, TCAMS). Chemical filtering and prioritization resulted in 47 different chemotypes with antimalarial potency (EC_50_) <2 μm. Herein we describe the selection of one particular series of inhibitors from the TCAMS library screen and the subsequent optimization of a new class of antimalarials, based on a substituted amidoacetamide core.

### Hit selection

**Figure 1:**
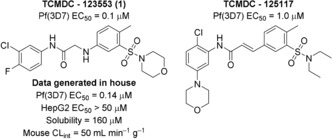
Compounds from TCAMS Series 10.^6^

Amidoacetamide TCMDC‐123553 (Series 10)^7^ was selected as a new chemical starting point based on good antimalarial potency against chloroquine/pyrimethamine‐sensitive and ‐resistant strains, suitable physicochemical properties for further development^8^ and was not subject to drug discovery efforts from any other MMV funded programs. Series 10 comprised a cluster of two compounds: TCMDC‐123553 and TCMDC‐125117 (Figure 1). The enamide moiety of TCMDC‐125117 was flagged as a possible Michael acceptor and with the added risk of epoxidation of the double bond to give a reactive intermediate.^9^ Therefore, our focus was directed toward TCMDC‐123553 (**1**) for further in vitro assessment.

**Figure 2:**
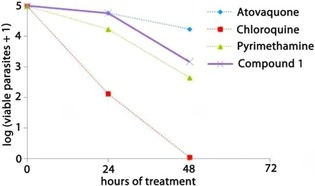
Parasite reduction ratio (PRR) assessment of compound **1**. Standard antimalarials atovaquone, chloroquine, and pyrimethamine were used as controls. Data for the controls were reported previously.^9^

As a suitable chemical starting point, TCMDC‐123553 (**1**) displayed good antimalarial activity in vitro and was inactive in a mammalian counter‐screen. In a preliminary malaria parasite rate of kill assay (the parasite reduction ratio (PRR) assay^10^), **1** showed a relatively slow rate of kill, intermediate between pyrimethamine and atovaquone (Figure 2). In this assay, parasites were treated with 10×EC_50_ concentrations of **1** and samples of parasites taken from treated cultures after 24 and 48 h, and the number of viable parasites determined.

Compound **1** was investigated for activity against other *Plasmodium* life‐cycle stages. In a *P. berghei* liver‐stage schizont assay,^11^ compound **1** was found to be inactive up to 50 μm. It displayed no effect in *P. cynomolgi* liver‐stage assay^12^ against both large forms and small forms (the latter indicative of *P. vivax* hypnozoites) at 10 μm. Therefore, compounds of this series do not appear to be suitable for malaria chemoprotection or radical cure. Compound **1** (incubated at a concentration of 1 μm) gave decreases of 99 and 81 % in male and female gamete formation (sexual stages found in the mosquito) assays,^13^ respectively. However, although **1** was an inhibitor of male and female gamete formation, it was inactive in a late‐stage (stage IV/V) gametocyte assay^14^ at 40 μm. This latter assay measures the ability of compounds to kill mature gametocytes. Based on the inhibition in the dual gamete formation assay the aminoacetamide series would be expected to show transmission blocking activity, but this would need to be confirmed in a standard membrane feeding assay, which uses mosquito‐based readouts.

In vitro assessment of **1** showed moderate solubility (160 μm) and poor metabolic stability in mouse liver microsomes (CL_int_>50 mL min^−1^ (g liver)^−1^). Therefore, our initial design efforts were focused on improving solubility and metabolic stability, whilst improving or retaining potency. To obtain sufficient compound exposure in vivo, our initial goal was to identify antimalarials displaying aqueous (kinetic) solubility of ideally >250 μm and in vitro metabolic clearance of <5 mL min^−1^ (g liver)^−1^, in mouse liver microsomes,^15^ for pharmacokinetic (PK) and efficacy experiments.

### Optimization of R^1^

**Figure 3:**
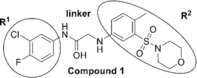
Developing structure–activity relationships (SARs).

Modifications at R^1^ (Figure 3 and Table 1) were initially investigated around the substituted phenyl ring. Modifications were focused on improving or retaining activity, whilst increasing metabolic stability and solubility. We envisaged that an increase in metabolic stability could be achieved by modifications to the phenyl ring by the introduction of heteroatoms to both modulate log*P* and remove potential sites of metabolism. Alternatively, replacement of the phenyl ring with aliphatic polar and/or basic groups, could simultaneously improve metabolic stability and aqueous solubility. Removal of the chlorine atom at the C3 position to afford fluorophenyl **2**, decreased lipophilicity and improved solubility, but resulted in a small drop in potency. Removal of the fluorine atom at C4 (**4**) had little effect on potency, whilst removal of both halogens (**3**) gave a 10‐fold decrease in activity. Furthermore, removal of one or both halogens had little effect on metabolic stability. Synthetic modifications at R^1^ were then directed toward aromatic heterocycles. Direct replacement of the di‐halogenated phenyl of **1** with a pyrazine (**5**) did not improve solubility or metabolic stability and lost all antimalarial activity. Thiazole (**6**) and oxazole (**7**) however, did improve solubility although all antimalarial activity was lost and metabolic stability was not improved.

**Table 1:** Modifications at R^1^.

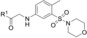	R^1^	EC_50_ [μm]^[a]^		*M*_r_ [Da]	clog*P* ^[b]^	Mouse microsomal CL_int_ [mL min^−1^ (g liver)^−1^]	Sol. [μm]^[c]^
		*Pf* (3D7)	HepG2				
**1**	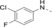	0.14 (*n*=6) CI 0.18–0.10	>50 (*n*=3)	442	2.4	>50	164
**2**	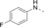	0.42 (*n*=4) CI 0.52–0.34	>25 (*n*=2)	407	2.1	45	>250
**3**	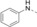	1.45 (*n*=4) CI 1.51–1.31	>25 (*n*=2)	389	2.0	42	183
**4**	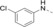	0.19 (*n*=4) CI 0.28–0.13	>25 (*n*=2)	424	2.3	>50	219
**5**	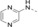	>50 (*n*=5)	4.98 (*n*=3) CI 17.59–1.64	391	1.5	42	55
**6** ^[d]^	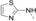	>50 (*n*=3)	>25 (*n*=2)	396	1.6	32	>250
**7**	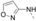	>50 (*n*=3)	>25 (*n*=2)	380	1.3	9.7	>250
**8**	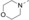	>50 (*n*=3)	>25 (*n*=2)	395	2.2	3.8	110
**9** ^[e]^	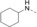	2.67 (*n*=3) CI 3.37–2.17	>25 (*n*=3)	383	0.8	37	20
**10**	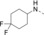	1.71 (*n*=6) CI 2.24–1.21	>25 (*n*=3)	431	2.2	19	>250
**11**	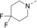	>50 (*n*=3)	>25 (*n*=2)	417	1.7	10	>250
**12**	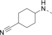	>50 (*n*=3)	>25 (*n*=2)	406	1.2	4.6	>250
**13**	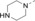	>50 (*n*=5)	>25 (*n*=3)	382	0.6	1.1	>250
**14**	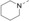	>50 (*n*=3)	>25 (*n*=2)	381	1.7	11	>250

[a] *n*: number of screening replicates; CI: 95 % confidence interval. [b] Values were calculated using StarDrop^®^ from Optibrium. [c] Kinetic solubility in water. [d] Purity: 84 %. [e] Purity: 81 %.

Despite the relatively low clog*P*, the compounds still showed poor metabolic stability. We then focused on aliphatic heterocycles. Substituting the R^1^ di‐halogenated aniline of **1** for morpholine **8**, lost all antimalarial activity, although led to an improvement in metabolic stability. Cyclohexylamine **9** retained some activity, but suffered from ≈20‐fold drop in antimalarial potency relative to compound **1**, and was metabolically unstable. The difluoro derivative (**10**) showed similar activity to compound **9**, but with significantly improved solubility, possibly due to the difluoro giving a small dipole moment.^16^ Replacement of the morpholine oxygen atom in **8** with difluoro **11** was also inactive, and lost metabolic stability relative to **8**. The addition of a nitrile group on the cyclohexyl ring of **9**, as exemplified by **12**, led to a loss of activity. Replacement of the R^1^ amino cyclohexyl moiety of **9** with a piperazine (**13**) improved metabolic stability and aqueous solubility but not metabolic stability and also lost all antimalarial activity. Substitution at R^1^ with piperidine **14** afforded a compound with lower solubility and microsomal stability than **13**, and again rendered the compound inactive. Efforts to improve potency and physicochemical properties were then directed toward changes at the R^2^ position.

### Optimization of R^2^

Substitution of the methyl group of **1** at R^2^ with fluorine to afford **16**, displayed a small drop in potency, but **16** was still rapidly metabolized (Table 2). Replacement of the methyl group on R^2^ with a chlorine atom (**15**), displayed a moderate improvement in potency, but aqueous solubility and metabolic stability were still poor. Removal of the methyl group altogether with **17**, did not improve metabolic stability or solubility and gave rise to a ≈100‐fold drop in potency. Modifications at R^2^ were then directed toward replacement of the morpholine sulfonamide moiety. Removal of the morpholine oxygen atom from **1** gave the piperidine sulfonamide **18**, which showed similar potency but increased lipophilicity and decreased aqueous solubility. The smaller and less lipophilic diethyl sulfonamide (**19**) and dimethyl sulfonamide (**20**) did not improve metabolic stability or solubility relative to **1**, although **19** retained potency, and **20** only showed a small drop. Replacement of the sulfonamide morpholine group of **1** with a morpholine amide (**21**), led to a loss of activity. However, compound **21** showed increased solubility, although the metabolic stability was still too high. To try and recover potency, we removed the carbonyl group of morpholine amide **21** to afford benzyl morpholine **22**. We envisaged that free rotation of the alkyl‐linked morpholine around the phenyl ring of R^2^ may allow the morpholine group to find an optimal interaction with the unknown biological target. However, **22** was inactive against malaria parasites. We then explored the bridged morpholine sulfonamide moiety **23**, to decrease the planarity of the molecule, with a view to increasing solubility through the possible disruption of crystal packing^17^ Compound **23** retained only a slightly reduced potency relative to compound **1**, and may have shown an marginal improvement in solubility but was very unstable with microsomes. We then moved to the, bridged homomorpholino to investigate if we could modulate activity with a larger sulfonamide. Compound **24** displayed similar potency to **23**, but decreased solubility. We then investigated changes to the linker functionality, to improve potency and modulate physicochemical properties.

**Table 2:** Modifications at R^2^.

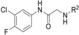	R^2^	EC_50_ [μm]^[a]^		*M*_r_ [Da]	clog*P* ^[b]^	Mouse microsomal CL_int_ [mL min^−1^ (g liver)^−1^]	Sol. [μm]^[c]^
		*Pf* (3D7)	HepG2				
**1**	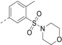	0.14 (*n*=6) CI 0.18–0.10	>25 (*n*=3)	442	2.4	>50	164
**15**	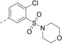	0.06 (*n*=7) CI 0.11–0.03	25.72 (*n*=3) CI 26.39–24.69	462	2.5	29	79
**16**	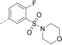	0.58 (*n*=3) CI 0.79–0.44	>25 (*n*=2)	446	2.3	48	219
**17** ^[d]^	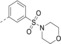	11 (*n*=5) CI 22–4.8	>25 (*n*=5)	427	2.1	>50	187
**18**	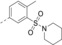	0.22 (*n*=4) CI 0.28–0.16	24.03 (*n*=3) CI 24.97–23.37	440	3.2	>50	55
**19**	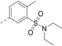	0.27 (*n*=4) CI 0.41–0.18	>25 (*n*=2)	428	2.2	>50	79
**20** ^[e]^	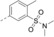	0.69 (*n*=4) CI 0.79–0.59	>25 (*n*=2)	400	2.3	>50	181
**21**	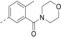	>50 (*n*=3)	>25 (*n*=2)	405	2.3	5.6	>250
**22**	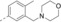	>50 (*n*=5)	>25 (*n*=3)	392	3.2	41	>250
**23** ^[f]^	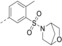	0.64(*n*=7 CI 1.0–0.35	>25 (*n*=3))	454	2.7	>50	219
**24**	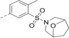	0.76 (*n*=3) CI 1.1–0.55	>25 (*n*=2)	468	2.9	>50	79

[a] *n*: number of screening replicates; CI: 95 % confidence interval. [b] Values were calculated using StarDrop^®^ from Optibrium. [c] Kinetic solubility in water. [d] Compound **17** was only weakly active, with two of five replicates returning an EC_50_ value greater than the top concentration tested. [e] Purity: 83 %. [f] Purity: 88 %.

### Increasing antimalarial activity: modifications to the linker

Initial changes to the aliphatic linker sought to determine the importance of the linker hydrogen bond donors for antimalarial activity, through the systematic methylation of each NH functionality. Methylation of the R^1^ amide NH (**25**) gave rise to a loss in potency. However, methylation of the R^2^ aniline NH (**26**) was tolerated although aqueous solubility and metabolic stability were not improved compared with **1**. Removal of the amide carbonyl to afford **27** resulted in >100‐fold drop in potency and also decreased aqueous solubility. We then investigated if the metabolic instability of **1** was associated with metabolism α to the carbonyl, which would also be N‐dealkylation, a known phase 1 biotransformation.^18^ A common approach to decrease metabolic instability is the geminal dimethylation of metabolically labile regions.^19^ The dimethylated analogue **28** did not show improved metabolic stability but displayed an approximate 20‐fold improvement in potency relative to hit compound **1** (Table 3).

**Table 3:** Modification of linker.

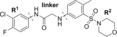	Linker	EC_50_ [μm]^[a]^		*M*_r_ [Da]	clog*P* ^[b]^	Mouse microsomal CL_int_ [mL min^−1^ (g liver)^−1^]	Sol. [μm]^[c]^
		*Pf* (3D7)	HepG2				
**1**	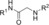	0.14 (*n*=6) CI 0.18–0.10	>25 (*n*=3)	442	2.4	>50	164
**25**	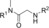	>50 (*n*=5)	>25 (*n*=3)	456	2.2	39	180
**26**	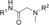	0.29 (*n*=3) CI 0.57–0.16	>25 (*n*=2)	456	2.8	>50	142
**27**	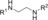	22.57 (*n*=3) CI 27.83–18.76	>25 (*n*=2)	428	2.6	37	39
**28**	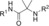	0.007 (*n*=7) CI 0.015–0.003	>25 (*n*=7)	470	3.0	>50	110

[a] *n*: number of screening replicates; CI: 95 % confidence interval. [b] Values were calculated using StarDrop^®^ from Optibrium. [c] Kinetic solubility in water.

### Met ID studies

To understand what was causing the microsomal instability, we sought to identify the chemical functional groups that were susceptible to metabolism, incubating mouse liver microsomes in the presence **1** and monitoring for metabolites by mass spectrometry. Two main metabolites were identified, [*M*+H]^+^ 458 and [*M*+H]^+^ 440, suggesting hydroxylation and dehydration. However, the exact sites of metabolism and metabolite structures were not clear from our Met ID experiments.

### Combining key SAR

A final attempt was made to investigate different combinations of previously tried functional groups, to improve physicochemical characteristics as well as potency against *P. falciparum* (Table 4). Substitution of the linker moiety with a *geminal* dimethyl group, combined with bridged morpholine analogues at R^2^, afforded **29** and **32**. Both gave a 10‐fold increase in potency relative to **1**. However, solubility and metabolic stability were not improved. New combinations at R^2^, replacing the methyl group with methoxy as exemplified by **30**, also resulted in a 10‐fold increase in potency and improved solubility relative to **1**, although again, metabolic stability was not improved. Substitution of the R^2^ morpholine on **1** with the spirocyclic morpholine sulfonamide of **31** displayed similar potency to **1** but the desired increased levels of metabolic stability and aqueous solubility were not obtained.

**Table 4:** Combining SAR.

No.	Key compounds	EC_50_ [μm]^[a]^		*M*_r_ [Da]	clog*P* ^[b]^	Mouse microsomal CL_int_ [mL min^−1^ (g liver)^−1^]	Sol. [μm]^[c]^
		*Pf* (3D7)	HepG2				
**1**	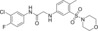	0.14 (*n*=6) CI 0.18–0.10	>25 (*n*=3)	442	2.4	>50	164
**28**	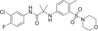	0.007 (*n*=7) CI 0.015–0.003	>25 (*n*=8)	470	3.0	>50	110
**29**	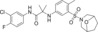	0.035 (*n*=6) CI 0.08–0.01	>25 (*n*=3)	496	3.5	>50	157
**30**	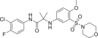	0.014 (*n*=3) CI 0.02–0.01	>25 (*n*=2)	470	3.0	40	219
**31**	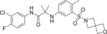	0.12 (*n*=3) CI 0.14–0.10	>25 (*n*=2)	470	3.0	22	167
**32**	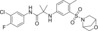	0.014 (*n*=5) CI 0.02–0.01	>25 (*n*=3)	482	3.3	>50	143

[a] *n*: number of screening replicates; CI: 95 % confidence interval. [b] Values were calculated using StarDrop^®^ from Optibrium. [c] Kinetic solubility in water.

## Conclusions

Through iterative rounds of drug design and synthesis, we were able to improve the antimalarial potency of **1** by >10‐fold to afford **28**, which displayed single digit nanomolar potency against *P. falciparum* and good cellular selectivity. It should be noted, however, that not all compounds in this series inhibited growth of *P. falciparum* to 100 % in vitro. Unfortunately, we were unable to identify compounds that combined both potency and good metabolic stability to progress to in vivo pharmacokinetic studies. Furthermore, we were not able to understand the reasons for the metabolic instability. Within this series, only compounds with very low lipophilicity (clog*P*<1) displayed the desired levels of microsomal stability (<5 mL min^−1^ (g liver)^−1^). Unfortunately, these compounds lost all antiparasitic activity. Therefore, during the optimization of the aminoacetamides, lowering lipophilicity was not sufficient to significantly decrease microsomal instability to the required levels. It should also be noted that compounds with very low log*P* values are likely to have a large free unbound fraction, which could lead to higher levels of clearance in vivo.^19^

Given the excellent antimalarial activity and selectivity of **28**, mode of action studies to determine the molecular target of this series may be valuable. These results may open new avenues to identify novel chemotypes with improved metabolic stability, aqueous solubility and potent antimalarial activity.

## Experimental Section

Please refer to the Supporting Information for all experimental details.

The human biological samples were sourced ethically, and their use in research was in accord with the terms of the informed consents under an IRB/EC‐approved protocol.

## Conflict of interest

*C.d.C., B.C., F.J.G., and L.M.S. are employees of GlaxoSmithKline and own shares of the company*.

## Supporting information

As a service to our authors and readers, this journal provides supporting information supplied by the authors. Such materials are peer reviewed and may be re‐organized for online delivery, but are not copy‐edited or typeset. Technical support issues arising from supporting information (other than missing files) should be addressed to the authors.

Supplementary
