# Supplementary material for: Substituted Aminoacetamides as Novel Leads for Malaria Treatment
Source: ChemMedChem. 2019 Jul 3;14(14):1329–35. doi: 10.1002/cmdc.201900329 (PMC6899483; doi:10.1002/cmdc.201900329)

CHEMMEDCHEM
Chemistry & Drug Discovery

Supporting Information

Substituted Aminoacetamides as Novel Leads for Malaria Treatment

Neil R. Norcross,[a] Caroline Wilson,[a] Beatriz Baragaña,[a] Irene Hallyburton,[a] Maria Osuna-Cabello,[a] Suzanne Norval,[a] Jennifer Riley,[a] Daniel Fletcher,[a] Robert Sinden,[b] Michael Delves,[b] Andrea Ruecker,[b] Sandra Duffy,[c] Stephan Meister,[d] Yevgeniya Antonova-Koch,[d] Benigno Crespo,[e] Cristina de Cózar,[e] Laura M. Sanz,[e] Francisco Javier Gamo,[e] Vicky M. Avery,[c] Julie A. Frearson,[a] David W. Gray,[a] Alan H. Fairlamb,[a] Elizabeth A. Winzeler,[d] David Waterson,[f] Simon F. Campbell,[f] Paul A. Willis,[f] Kevin D. Read,*[a] and Ian H. Gilbert*[a]

cmdc_201900329_sm_miscellaneous_information.pdf

SUPPORTING INFORMATION

Experimental

General. Reactions using microwave irradiation were carried out in a Biotage Initiator microwave. Normal phase TLC was carried out on precoated silica plates (Kieselgel 60 F254, BDH) with visualization via UV light (UV 254/365 nm) and/or ninhydrin solution. Flash chromatography was performed using Combiflash Companion Rf (Teledyne ISCO) and prepacked silica gel columns purchased from Grace Davison Discovery Science or SiliCycle. Mass-directed preparative HPLC separations were performed using a Waters HPLC (2545 binary gradient pumps, 515 HPLC make-up pump, 2767 sample manager) connected to a Waters 2998 photodiode array and a Waters 3100 mass detector. Preparative HPLC separations were performed with a Gilson HPLC (321 pumps, 819 injection module, 215 liquid handler/injector) connected to a Gilson 155 UV/vis detector. On both instruments, HPLC chromatographic separations were conducted using Waters XBridge C18 columns, 19 mm × 100 mm, 5 µm particle size, using 0.1% ammonia in water (solvent A) and acetonitrile (solvent B) as mobile phase. 1H NMR, DEPTQ NMR and 19F NMR spectra were recorded on a Bruker Avance DPX 500 spectrometer (1 H at 500.1 MHz, 13C at 125 MHz, 19F at 470.5 MHz) or a Bruker Avance DPX 400 (1 H at 400 MHz). Chemical shifts (δ) are expressed in ppm recorded using the residual solvent as the internal reference in all cases. Signal splitting patterns are described as singlet (s), doublet (d), triplet (t), quartet (q), multiplet (m), broad (br), or a combination thereof. Coupling constants (J) are quoted to the nearest 0.5 Hz. Low resolution electrospray (ES) mass spectra were recorded on a Bruker MicroTof mass spectrometer, run in positive mode. High resolution mass spectrometry (HRMS) was performed using a Bruker MicrOTOF mass spectrometer. LCMS analysis and chromatographic separation were conducted with a Bruker MicrOTOF mass spectrometer or an Agilent Technologies 1200 series HPLC connected to an Agilent Technologies 6130 quadrupole LC/MS, where both instruments were connected to an Agilent diode array detector. The column used was a Waters XBridge column (50 mm × 2.1 mm, 3.5 µm particle size) and the compounds were eluted with a gradient of 5−95% acetonitrile/ water + 0.1% ammonia. All compounds for in vitro and in vivo experiments displayed >95% purity by LCMS. Unless otherwise stated herein reactions have not been optimized. Solvents and reagents were purchased from commercial suppliers and used without further purification. Dry solvents were purchased in Sure/Seal bottles stored over molecular sieves.

Biology and Pharmacology Methodology

Plasmodium falciparum screening, mammalian cell growth inhibition assay, kinetic aqueous solubility and mouse microsomal clearance were all performed as previously described, [1, 2] except growth inhibition curves, which were fitted by non-linear regression to the following 4-parameter equation:

$$y = a + \frac{b - a}{1 + \left(\frac{E}{EC_{50}}\right)^{c}}$$

Where a is the minimum value that can be obtained (i.e. what happens at zero dose), b is the maximum value that can be obtained (i.e. what happens at infinite dose), E is the concentration of effector, EC_{50} is the concentration of effector that produces 50% of maximum effect and c is the hill slope of the curve.

Chemistry

General Scheme

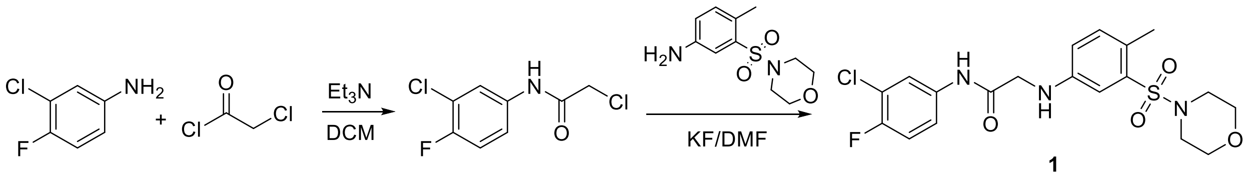

1

Compound 1 was typically prepared in two steps; 1, addition-elimination of chloroacetyl chloride with a corresponding aniline to form the chloroacetamide intermediate; 2, addition-elimination of chloroacetamide with another corresponding aniline, in the presence of potassium iodide.

Experimental

General procedure A:

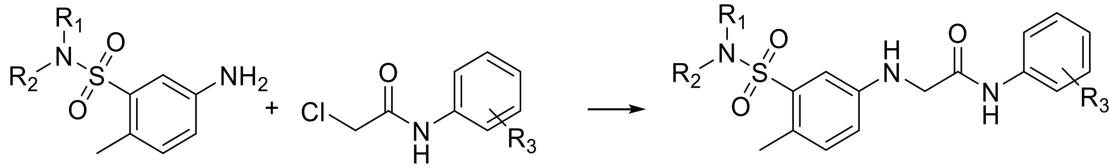

A mixture of the corresponding chloroacetamide (0.14 mmol) and potassium iodide (68 mg, 0.41 mmol) in anhydrous DMF (0.5 mL) was stirred at 45ºC for 30 mins then the corresponding aniline (0.13 mmol) was added and the reaction mixture stirred at 45ºC overnight. The reaction mixture was then diluted to 1.2 ml with DMF and purified by HPLC 0-95% MeCN, basic method to afford the title compounds.

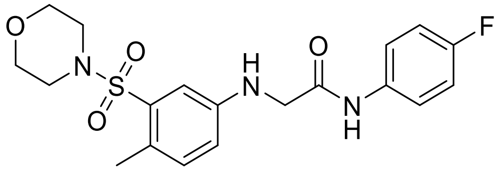

Compound 2

N-(4-fluorophenyl)-2-((4-methyl-3-(morpholinosulfonyl)phenyl)amino)acetamide Prepared using general procedure **A**. Yield, 45% (25 mg); ^{1}H NMR (500 MHz, CDCl3) δ 8.30 (s, 1H), 7.51 - 7.48 (m, 2H), 7.25 - 7.19 (m, 2H), 7.05 - 7.01 (m, 2H), 6.79 (dd, J=2.7, 8.2 Hz, 1H), 4.53 - 4.50 (m, 1H), 3.95 (d, J=5.2 Hz, 2H), 3.69 (t, J=4.7 Hz, 4H), 3.12 (t, J=4.7 Hz, 4H), 2.53 (s, 3H); ^{13}C NMR (126 MHz, DMSO-d_6) δ 19.3, 45.3, 46.9, 65.4, 65.5, 112.5, 115.3 (d, J = 22.0 Hz), 117.1, 120.9 (d, J = 7.8 Hz), 123.8, 133.4, 134.2, 135.2 (d, J = 2.6 Hz), 146.7, 158.0 (d, J = 239.9 Hz), 168.7; ^{19} F NMR (470.5 MHz; d^{6}-DMSO) δ -119.27; LRMS (ES^{+}) m/z 408 $[M+H]^{+}$.

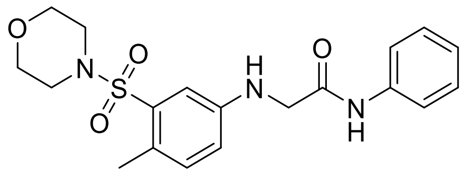

Compound 3

((4-methyl-3-(morpholinosulfonyl)phenyl)amino)-N-phenylacetamide Prepared using general procedure **A**. Yield, 47% (19 mg); ^{1}H NMR (500 MHz, CDCl3) δ 8.30 (s, 1H), 7.53 (d, J=7.6 Hz, 2H), 7.36 - 7.31 (m, 2H), 7.24 - 7.11 (m, 3H), 6.80 (dd, J=2.7, 8.4 Hz, 1H), 4.55 - 4.52 (m, 1H), 3.96 (d, J=5.2 Hz, 2H), 3.66 (t, J=4.8 Hz, 4H), 3.10 (t, J=4.7 Hz, 4H), 2.53 (s, 3H); LRMS (ES^{+}) m/z 390 [M+H]$^{+}$.

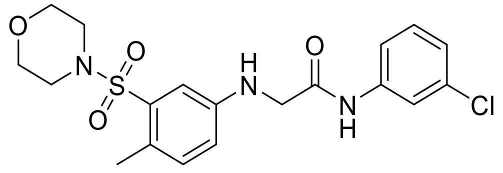

Compound 4

N-(3-chlorophenyl)-2-((4-methyl-3-(morpholinosulfonyl)phenyl)amino)acetamide Prepared using general procedure **A**. Yield, 43% (23 mg); ^{1}H NMR (500 MHz, CDCl3) δ 8.39 (s, 1H), 7.67 (dd, J=2.0, 2.0 Hz, 1H), 7.40 (dd, J=1.1, 8.2 Hz, 1H), 7.29 (s, 3H), 7.14 - 7.12 (m, 1H), 6.80 (dd, J=2.7, 8.2 Hz, 1H), 4.56 (t, J=5.4 Hz, 1H), 3.97 (d, J=5.4 Hz, 2H), 3.70 (t, J=4.7 Hz, 4H), 3.14 (t, J=4.7 Hz, 4H), 2.55 (s, 3H) ; LRMS (ES^{+}) m/z 424 [M+H]$^{+}$.

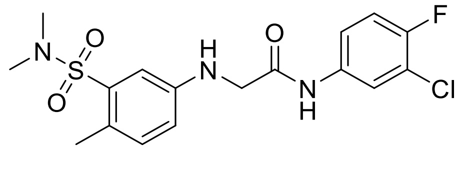

Compound 20

N-(3-chloro-4-fluorophenyl)-2-((3-(N,N-dimethylsulfamoyl)-4-methylphenyl)amino)acetamide Prepared using general procedure **A**. Yield, 45% (19 mg); ^{1}H NMR (500 MHz, CDCl3) δ 8.44 (s, 1H), 7.73 (dd, J=2.7, 6.5 Hz, 1H), 7.37 (ddd, J=2.7, 4.0, 8.9 Hz, 1H), 7.29 (s, 1H), 7.20 (d, J=8.2 Hz, 1H), 7.10 (dd, J=8.7, 8.7 Hz, 1H), 6.76 (dd, J=2.7, 8.2

Hz, 1H), 4.56 (t, J=5.4 Hz, 1H), 3.96 (d, J=5.5 Hz, 2H), 2.82 (s, 6H), 2.53 (s, 3H) ; LRMS (ES^+) m/z 799 $[2M+H]^+$.

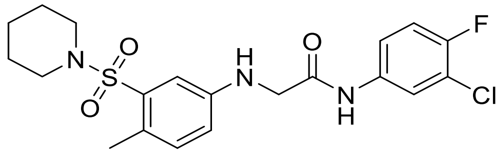

Compound 18

N-(3-chloro-4-fluorophenyl)-2-((4-methyl-3-(piperidin-1-ylsulfonyl)phenyl)amino)acetamide Prepared using general procedure **A**. Yield, 41% (25 mg); ^{1}H NMR (500 MHz, CDCl3) δ 8.43 (s, 1H), 7.73 (dd, J=2.5, 6.5 Hz, 1H), 7.38 (ddd, J=2.7, 4.0, 8.9 Hz, 1H), 7.29 (s, 2H), 7.10 (dd, J=8.7, 8.7 Hz, 1H), 6.76 (dd, J=2.7, 8.2 Hz, 1H), 4.53 (t, J=5.5 Hz, 1H), 3.96 (d, J=5.5 Hz, 2H), 3.15 (t, J=5.4 Hz, 4H), 2.53 (s, 3H), 1.63 - 1.57 (m, 6H) ; LRMS (ES^+) m/z 440 $[M+H]^+$.

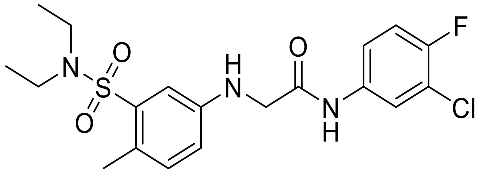

Compound 19

N-(3-chloro-4-fluorophenyl)-2-((3-(N,N-diethylsulfamoyl)-4-methylphenyl)amino)acetamide Prepared using general procedure **A**. Yield, 53% (31 mg); ^{1}H NMR (500 MHz, CDCl3) δ 8.45 (s, 1H), 7.73 (dd, J=2.6, 6.5 Hz, 1H), 7.37 (ddd, J=2.7, 4.1, 8.9 Hz, 1H), 7.29 (s, 1H), 7.17 (d, J=8.2 Hz, 1H), 7.10 (t, J=8.7 Hz, 1H), 6.74 (dd, J=2.7, 8.2 Hz, 1H), 4.54 (t, J=5.5 Hz, 1H), 3.95 (d, J=5.5 Hz, 2H), 3.31 (q, J=7.1 Hz, 4H), 2.50 (s, 3H), 1.14 (t, J=7.1 Hz, 6H) ; LRMS (ES^+) m/z 428 $[M+H]^+$.

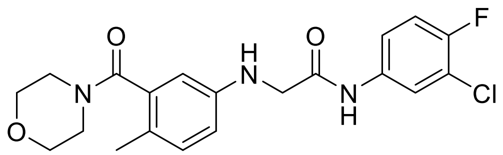

Compound 21

N-(3-chloro-4-fluorophenyl)-2-((4-methyl-3-(morpholine-4-carbonyl)phenyl)amino)acetamide

A mixture of 2-chloro-N-(3-chloro-4-fluoro-phenyl)acetamide (30 mg, 0.14 mmol), potassium iodide (68 mg, 0.41 mmol) in anhydrous DMF (0.5 mL) was heated at 45ºC for 30 mins then (5-amino-2-methyl-phenyl)-morpholino-methanone (30 mg, 0.14 mmol) was added and the reaction mixture stirred at 45ºC overnight. The cooled reaction mixture was purified by HPLC 5-95% MeCN, basic method. The product obtained was freeze-dried to afford N-(3-chloro-4-fluorophenyl)-2-((4-methyl-3-(morpholine-4-carbonyl)phenyl)amino)acetamide (16 mg, 0.038 mmol, 28%) as a white solid.

^{1}H NMR (500 MHz, Acetone) δ 9.47 (s, 1H), 8.00 (dd, J=2.5, 6.8 Hz, 1H), 7.58 - 7.54 (m, 1H), 7.22 (dd, J=9.1, 9.1 Hz, 1H), 7.03 (d, J=8.4 Hz, 1H), 6.62 (dd, J=2.5, 8.4 Hz, 1H), 6.46 (d, J=2.5 Hz, 1H), 5.51 (dd, J=5.6, 5.6 Hz, 1H), 3.94 - 3.88 (m, 2H), 3.66 (s, 4H), 3.48 (s, 2H), 3.20 - 3.19 (m, 2H), 2.12 (s, 3H); ^{13}C NMR (126 MHz, DMSO-d_6) δ 17.5, 41.2, 46.7, 47.3, 66.1, 66.2, 109.3, 113.0, 116.9 (d, *J* = 21.7 Hz), 119.0 (d, *J* = 18.2 Hz), 119.5 (d, *J* = 6.8 Hz), 120.6, 120.9, 130.7, 136.0 (d, *J* = 3.0 Hz), 136.2, 146.2, 153.1 (d, *J* = 242.6 Hz), 169.1, 169.6; ^{19} F NMR (470.5 MHz; d^6-DMSO) δ -122.80; LRMS (ES$^+$) m/z 406 [M+H]$^+$.

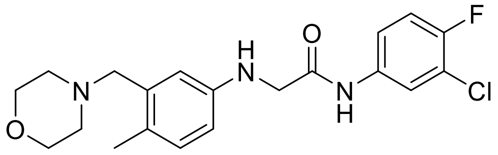

Compound 22

N-(3-chloro-4-fluorophenyl)-2-((4-methyl-3-(morpholinomethyl)phenyl)amino)acetamide

A mixture of 2-chloro-N-(3-chloro-4-fluoro-phenyl)acetamide (108 mg,0.49 mmol) in DMF was prepared at rt and iodopotassium (241 mg,1.45 mmol) added and the mixture heated to 45-48°C for 1h. The mixture was then cooled to rt and 4-methyl-3-(morpholinomethyl)aniline (100 mg, 0.49 mmol) added in one portion and the mixture heated again to 45-48°C in a sealed tube for 17 h. The mixture was then diluted with DCM (20 mL) and filtered through a SCX-2 column and washed with DCM (2 x 10 mL), 10% MeOH/DCM (2 x 10 mL) and finally, flushed with 7M ammonia methanol (20 mL) and the filtrate concentrated under reduced pressure and purified by

column chromatography (0-5% methanol/DCM) to afford the desired product (121 mg, 0.31 mmol, 64 %) as a colourless oil.

^{1}H NMR (500 MHz, CDCl3) d 8.63 (s, 1H), 7.68 (dd, J=2.7, 6.5 Hz, 1H), 7.38 - 7.34 (m, 1H), 7.09 - 7.01 (m, 2H), 6.70 (d, J=2.2 Hz, 1H), 6.50 (dd, J=2.7, 8.0 Hz, 1H), 4.19 (t, J=5.4 Hz, 1H), 3.89 (d, J=5.5 Hz, 2H), 3.65 (t, J=4.6 Hz, 4H), 3.39 (s, 2H), 2.44 - 2.41 (m, 4H), 2.26 (s, 3H); LRMS (ES^{+}) m/z 392 [M+H]$^{+}$.

General Procedure B

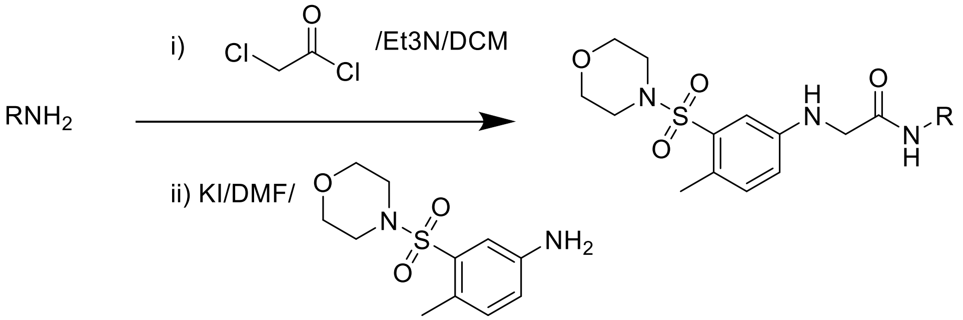

A mixture of the corresponding amine (0.14 mmol) in anhydrous DCM (1 ml) was treated with triethylamine (16 mg, 0.16 mmol) and then 2-chloroacetyl chloride (15 mg, 0.14 mmol). The tube was then stoppered and stirred at room temperature overnight. The reaction mixture was treated with water (1 ml) and the layers separated with a hydrophobic frit, the aqueous was extracted with further DCM (1 ml). The solvent extract was evaporated. The residue was dissolved in anhydrous DMF (0.5 ml) and potassium iodide (68 mg, 0.41 mmol) was added. The mixture was heated at 45ºC for 45 mins. Then 4-methyl-3-morpholinosulfonyl-aniline (35 mg, 0.14 mmol) was added and the mixture stirred at 45ºC overnight. The reaction mixture was then purified by HPLC 0-95% MeCN, basic method to afford the title compounds.

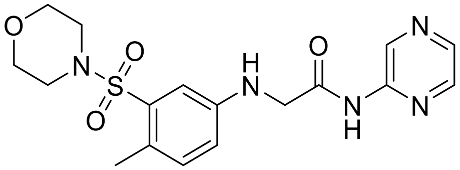

Compound 5

2-((4-methyl-3-(morpholinosulfonyl)phenyl)amino)-N-(pyrazin-2-yl)acetamide Prepared using general procedure **B**. Yield, 20% (11 mg); ^{1}H NMR (500 MHz, Acetone) δ 9.46 (d, J=1.4 Hz, 1H), 8.34 (d, J=2.5 Hz, 1H), 8.30 - 8.29 (m, 1H), 7.22 - 7.18 (m, 2H), 6.94 (dd, J=2.6, 8.3

Hz, 1H), 4.17 (d, J=6.3 Hz, 2H), 3.61 - 3.59 (m, 4H), 3.04 - 3.01 (m, 4H), 2.47 (s, 3H) ; LRMS (ES^+) m/z 392 $[M+H]^+$.

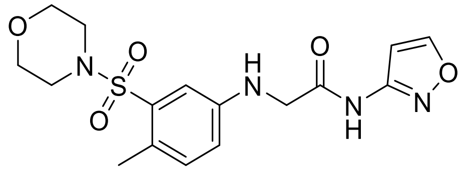

Compound 7

N-(isoxazol-3-yl)-2-((4-methyl-3-(morpholinosulfonyl)phenyl)amino)acetamide Prepared using general procedure **B**. Yield, 26% (14 mg); ^{1}H NMR (500 MHz, Acetone) δ 8.60 (d, J=1.7 Hz, 1H), 7.19 (d, J=8.4 Hz, 1H), 7.14 (d, J=2.7 Hz, 1H), 7.02 (d, J=1.4 Hz, 1H), 6.89 (dd, J=2.6, 8.3 Hz, 1H), 5.84 (dd, J=5.7, 5.7 Hz, 1H), 4.13 (d, J=6.0 Hz, 2H), 3.63 - 3.60 (m, 4H), 3.03 - 3.01 (m, 4H), 2.47 (s, 3H); ^{13}C NMR (126 MHz, DMSO-d_6) δ 19.3, 45.3, 46.3, 65.4, 98.9, 112.5, 116.9, 123.8, 133.5, 134.3, 146.5, 157.3, 160.2, 169.4. ; LRMS (ES^+) m/z 381 $[M+H]^+$.

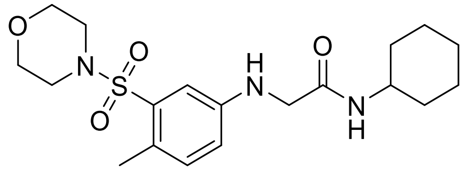

Compound 9

N-cyclohexyl-2-((4-methyl-3-(morpholinosulfonyl)phenyl)amino)acetamide Prepared using general procedure **B**. Yield, 52% (28 mg); ^{1}H NMR (500 MHz, Acetone) δ 7.17 (d, J=8.2 Hz, 1H), 7.09 (d, J=2.7 Hz, 2H), 6.81 (dd, J=2.6, 8.3 Hz, 1H), 3.73-3.65 (m, 7H), 3.08 - 3.05 (m, 4H), 2.47 (s, 3H), 1.85 - 1.78 (m, 2H), 1.70 - 1.64 (m, 2H), 1.59 - 1.53 (m, 1H), 1.37 - 1.27 (m, 2H), 1.23 - 1.11 (m, 3H); ^{13}C NMR (126 MHz, DMSO-d_6) δ 19.3, 24.4, 25.1, 32.3, 45.2, 46.5, 47.4, 65.5, 112.8, 116.8, 123.6, 133.3, 134.4, 146.7, 168.4; LRMS (ES^+) m/z 791 $[2M+H]^+$.

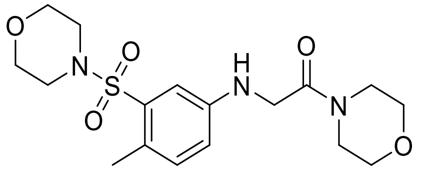

Compound 8

2-((4-methyl-3-(morpholinosulfonyl)phenyl)amino)-1-morpholinoethan-1-one Prepared using general procedure **B**. Yield, 31% (17 mg); ^{1}H NMR (500 MHz, Acetone) δ 7.17 - 7.15 (m,

2H), 6.91 (dd, J=2.6, 8.3 Hz, 1H), 4.03 (d, J=4.1 Hz, 2H), 3.69 - 3.55 (m, 12H), 3.08 - 3.05 (m, 4H), 2.47 (s, 3H) ; ^{13}C NMR (126 MHz, DMSO-d_6) δ 19.3, 41.7, 44.3, 44.6, 45.3, 65.5, 66.0, 66.0, 113.1, 116.6, 123.2, 133.3, 134.5, 146.6, 167.7; LRMS (ES$^+$) m/z 384 [M+H]$^+$.

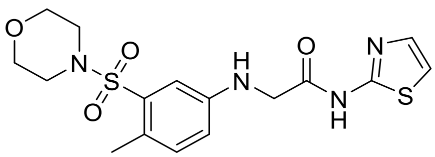

Compound 6

2-((4-methyl-3-(morpholinosulfonyl)phenyl)amino)-N-(thiazol-2-yl)acetamide

A mixture of thiazol-2-amine (14 mg, 0.14 mmol) in anhydrous DCM (1 ml) was treated with triethylamine (16 mg, 0.16 mmol) and then 2-chloroacetyl chloride (15 mg, 0.14 mmol). The tube was then stoppered and stirred at room temperature overnight. The reaction mixture was treated with water (1 ml) and the layers separated with a hydrphobic frit, the aqueous was extracted with further DCM (1 ml) and then EtOAc. The solvent extracts were evaporated. The residue was dissolved in anhydrous DMF (0.5 ml) and potassium iodide (68 mg, 0.41 mmol) was added. The mixture was heated at 45ºC for 45 mins. Then 4-methyl-3-morpholinosulfonyl-aniline (35 mg, 0.14 mmol) was added and the mixture stirred at 45ºC overnight. The reaction mixture were then purified by HPLC 0-95% MeCN, basic method to afford 2-((4-methyl-3-(morpholinosulfonyl)phenyl)amino)-N-(thiazol-2-yl)acetamide (14 mg, 0.035 mmol, 26%)

^{1}H NMR (500 MHz, Acetone) δ 7.40 (d, J=3.5 Hz, 1H), 7.19 (d, J=8.4 Hz, 1H), 7.14 - 7.12 (m, 2H), 6.91 (dd, J=2.6, 8.3 Hz, 1H), 4.22 (d, J=6.1 Hz, 2H), 3.59 - 3.56 (m, 4H), 3.00 - 2.97 (m, 4H), 2.47 (s, 3H) ; LRMS (ES$^+$) m/z 397 [M+H]$^+$.

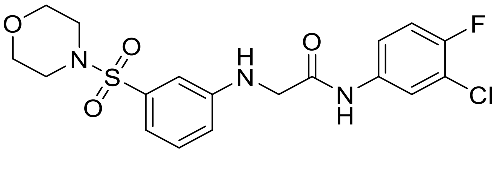

Compound 17

N-(3-chloro-4-fluorophenyl)-2-((3-(morpholinosulfonyl)phenyl)amino)acetamide

A mixture of 2-chloro-N-(3-chloro-4-fluoro-phenyl)acetamide (27 mg, 0.12 mmol), potassium iodide (62 mg, 0.37 mmol) in anhydrous DMF (0.5 mL) was heated at 45ºC for 45 mins. 3-morpholinosulfonylaniline (30 mg, 0.12 mmol) was added and the reaction mixture heated at 45ºC overnight. The reaction mixture was then purified by HPLC 5-95% MeCN, basic method to afford

N-(3-chloro-4-fluorophenyl)-2-((3-(morpholinosulfonyl)phenyl)amino)acetamide (20 mg, 0.044 mmol, 35%) as a white solid.

^{1}H NMR (500 MHz, DMSO) δ 10.33 (s, 1H), 7.93 (dd, J=2.5, 6.8 Hz, 1H), 7.51 (ddd, J=2.6, 4.3, 9.0 Hz, 1H), 7.41 - 7.34 (m, 2H), 6.97 - 6.89 (m, 2H), 6.85 (dd, J=2.0, 2.0 Hz, 1H), 6.70 (dd, J=6.1, 6.1 Hz, 1H), 3.97 (d, J=6.1 Hz, 2H), 3.53 (t, J=4.7 Hz, 4H), 2.78 (t, J=4.7 Hz, 4H); ^{13}C NMR (126 MHz, DMSO-d_6) δ 45.8, 46.7, 65.2, 109.8, 115.0, 117.0 (d, *J* = 21.8 Hz), 117.2, 119.1 (d, *J* = 18.3 Hz), 119.5 (d, *J* = 6.9 Hz), 120.6, 129.7, 134.8, 136.0 (d, *J* = 3.1 Hz), 149.0, 153.1 (d, *J* = 242.5 Hz), 169.0; ^{19}F NMR (470.5 MHz; d^6-DMSO) δ -122.65; LRMS (ES^+) m/z 428 $[M+H]^+$.

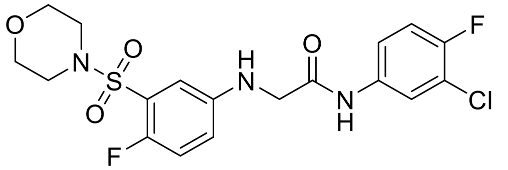

Compound 16

N-(3-chloro-4-fluorophenyl)-2-((4-fluoro-3-(morpholinosulfonyl)phenyl)amino)acetamide

A mixture of 2-chloro-N-(3-chloro-4-fluoro-phenyl)acetamide (25 mg, 0.11 mmol), potassium iodide (57 mg, 0.34 mmol) in anhydrous DMF (0.5 mL) was stirred at 45ºC for 30 mins then 4-fluoro-3-morpholinosulfonyl-aniline (30 mg, 0.11 mmol) was added and the reaction mixture stirred at 45ºC overnight. The reaction mixture was then filtered and purified by mass directed HPLC, 5-95% MeCN, basic method to afford N-(3-chloro-4-fluorophenyl)-2-((4-fluoro-3-(morpholinosulfonyl)phenyl)amino)acetamide (19 mg, 0.04 mmol, 35%) as a cream coloured solid.

^{1}H NMR (300 MHz, DMSO) δ 10.31 (s, 1H), 7.92 (dd, J=2.4, 6.8 Hz, 1H), 7.54 - 7.35 (m, 2H), 7.25 (dd, J=8.9, 10.1 Hz, 1H), 6.96 - 6.84 (m, 2H), 6.57 (t, J=6.1 Hz, 1H), 3.94 (d, J=6.2 Hz, 2H), 3.58 (m, 4H), 2.96 - 2.90 (m, 4H); ^{13}C NMR (126 MHz, DMSO-d_6) δ 45.6, 47.1, 65.3, 112.2, 116.9 (d, *J* = 21.7 Hz), 118.0 (d, *J* = 23.3 Hz), 118.5 (d, *J* = 7.2 Hz), 119.1 (d, *J* = 18.4 Hz), 119.5 (d, *J* = 6.8 Hz), 120.6, 122.7 (d, *J* = 15.3 Hz), 135.9 (d, *J* = 3.1 Hz), 145.2, 150.1 (d, *J* = 242.5 Hz), 153.1 (d, *J* = 242.9 Hz), 168.9; ^{19}F NMR (470.5 MHz; d^6-DMSO) δ -122.65, 125.65; LRMS (ES^+) m/z 446 $[M+H]^+$.

General Procedure C

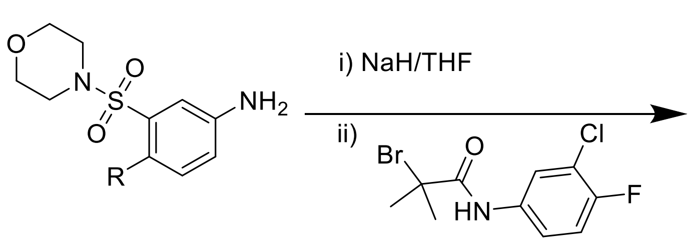

A solution of the corresponding aniline (0.12 mmol) in anhydrous THF (2 ml) was cooled to 0ºC under nitrogen and treated with sodium hydride (6 mg, 0.14 mmol) and stirred for 10 mins under nitrogen. 2-bromo-N-(3-chloro-4-fluoro-phenyl)-2-methyl-propanamide (69 mg, 0.23 mmol) was added and the reaction mixture stirred at room temperature under nitrogen overnight. The reaction mixture was quenched with water (5 ml) and extracted with EtOAc (10 ml). The EtOAc extract was evaporated to give a yellow oil. The residue was dissolved in DMF and purified by HPLC 5-95% MeCN, basic method. The residue was dissolved in methanol and purified by SCX (2 g) eluting with MeOH/ 2M NH3/MeOH to afford the title compounds.

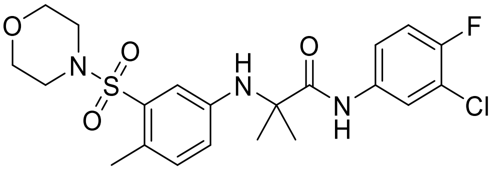

Compound 28

N-(3-chloro-4-fluorophenyl)-2-methyl-2-((4-methyl-3-(morpholinosulfonyl)phenyl)amino)propanamide Prepared using general procedure **C.** Yield 13% (8 mg); ^{1}H NMR (500 MHz, Acetone) δ 9.64 (s, 1H), 8.00 (ddd, J=1.1, 2.6, 6.8 Hz, 1H), 7.60 - 7.56 (m, 1H), 7.24 - 7.15 (m, 2H), 7.10 - 7.09 (m, 1H), 6.79 (dd, J=2.0, 8.3 Hz, 1H), 3.55 - 3.53 (m, 4H), 2.95 - 2.93 (m, 4H), 2.45 (s, 3H), 1.55 (d, J=1.3 Hz, 6H); LRMS (ES$^+$) m/z 470 [M+H]$^+$.

General procedure D:

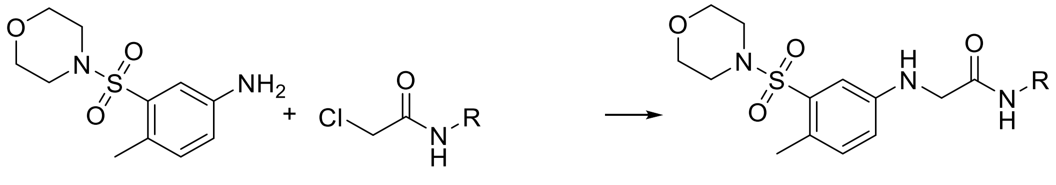

A mixture of the corresponding chloroacetamide (0.12 mmol), potassium iodide (58 mg, 0.35 mmol) in anhydrous DMF (0.5 mL) was stirred at 45ºC for 30 mins then 4-methyl-3-

morpholinosulfonyl-aniline (30 mg, 0.12 mmol) was added and the reaction mixture stirred at 45°C overnight. The reaction mixture was filtered and purified by mass directed HPLC, 5-95% MeCN, basic method, to afford the title compounds.

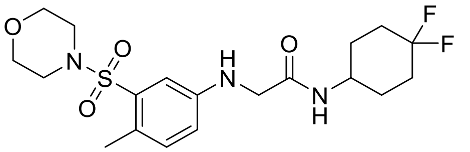

Compound 10

N-(4,4-difluorocyclohexyl)-2-((4-methyl-3-(morpholinosulfonyl)phenyl)amino)acetamide Prepared using general procedure **D**. Yield, 30% (15 mg); ^{1}H NMR (500 MHz, DMSO) δ 7.97 (d, J=7.8 Hz, 1H), 7.15 (d, J=8.5 Hz, 1H), 6.95 (d, J=2.6 Hz, 1H), 6.76 (dd, J=2.6, 8.2 Hz, 1H), 6.27 (t, J=6.0 Hz, 1H), 3.81 - 3.78 (m, 1H), 3.67 - 3.60 (m, 6H), 3.10 - 2.96 (m, 4H), 2.52 - 2.39 (m, 3H), 2.02 - 1.73 (m, 6H), 1.54 - 1.38 (m, 2H); LRMS (ES$^+$) m/z 863 [2M+H]$^+$.

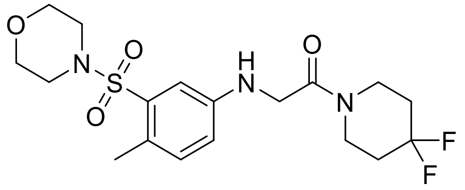

Compound 11

1-(4,4-difluoropiperidin-1-yl)-2-((4-methyl-3-(morpholinosulfonyl)phenyl)amino)ethan-1-one Prepared using general procedure **D**. Yield, 28% (13 mg); ^{1}H NMR (500 MHz, DMSO) δ 7.15 (d, J=8.5 Hz, 1H), 7.06 (d, J=2.6 Hz, 1H), 6.85 (dd, J=2.5, 8.3 Hz, 1H), 6.10 (t, J=5.3 Hz, 1H), 4.02 (d, J=5.3 Hz, 2H), 3.64 - 3.58 (m, 8H), 3.00 (t, J=4.7 Hz, 4H), 2.52 - 2.40 (m, 3H), 2.10 - 1.89 (m, 4H); ^{13}C NMR (126 MHz, DMSO-d_6) δ 19.3, 33.2 (t, J = 23.2 Hz), 33.7 (t, J = 23.2 Hz), 38.4 (t, J = unresolved Hz), 40.9 (t, J = unresolved), 44.4, 45.3, 65.5, 113.1, 116.6, 122.6 (t, J = 241.0 Hz), 123.3, 133.3, 134.5, 146.6, 167.7; ^{19} F NMR (470.5 MHz; d^6-DMSO) δ -95.55; LRMS (ES$^+$) m/z 418 [M+H]$^+$.

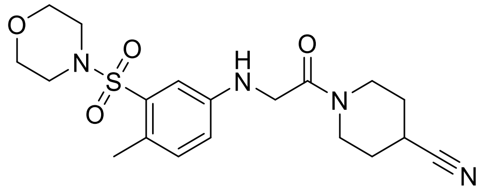

Compound 12

1-((4-methyl-3-(morpholinosulfonyl)phenyl)glycyl)piperidine-4-carbonitrile Prepared using general procedure **D**. Yield, 29% (13 mg); ^{1}H NMR (500 MHz, DMSO) δ 7.14 (d, J=8.4 Hz, 1H), 7.05 (d, J=2.4 Hz, 1H), 6.84 (dd, J=2.6, 8.4 Hz, 1H), 6.08 (dd, J=5.3, 5.3 Hz, 1H), 3.96 (d, J=5.3 Hz, 2H), 3.83 – 3.76 (m, 1H), 3.71 – 3.64 (m, IH), 3.64 - 3.61 (m, 4H), 3.39 - 3.34 (m, 1H), 3.30 - 3.21 (m, 1H), 3.16 - 3.11 (m, 1H), 3.02-2.97 (m, 4H), 2.40 (s, 3H), 1.95 - 1.92 (m, 1H), 1.87 - 1.84 (m, 1H), 1.78 - 1.71 (m, 1H), 1.65 - 1.57 (m, 1H); ^{13}C NMR (126 MHz, DMSO-d_6) δ 19.3, 25.3, 27.8, 28.3, 39.6, 42.4, 44.4, 45.3, 65.5, 113.1, 116.6, 121.9, 123.2, 133.3, 134.4, 146.6, 167.4; LRMS (ES$^+$) m/z 407 [M+H]$^+$.

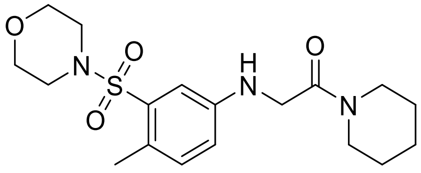

Compound 14

2-((4-methyl-3-(morpholinosulfonyl)phenyl)amino)-1-(piperidin-1-yl)ethan-1-one Prepared using general procedure **D**. Yield, 33% (16 mg); ^{1}H NMR (500 MHz, Acetone) δ 7.19 - 7.16 (m, 2H), 6.92 (dd, J=2.5, 8.4 Hz, 1H), 5.47 (s, 1H), 4.00 (d, J=4.4 Hz, 2H), 3.69 - 3.67 (m, 4H), 3.59 - 3.50 (m, 4H), 3.11 - 3.08 (m, 4H), 2.48 (s, 3H), 1.71 - 1.62 (m, 4H), 1.54 (d, J=4.9 Hz, 2H); ^{13}C NMR (126 MHz, DMSO-d_6) δ 19.3, 23.9, 25.3, 25.9, 42.3, 44.4, 45.0, 45.3, 65.5, 113.0, 116.6, 123.1, 133.3, 134.4, 146.6, 167.0; LRMS (ES$^+$) m/z 382 [M+H]$^+$.

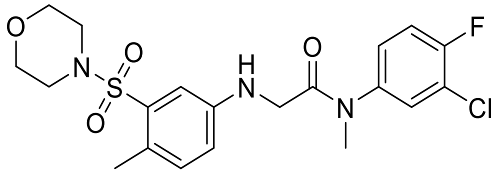

Compound 25

N-(3-chloro-4-fluorophenyl)-N-methyl-2-((4-methyl-3-(morpholinosulfonyl)phenyl)amino)acetamide Prepared using general procedure **D**. Yield,

38% (21 mg); ^{1}H NMR (500 MHz, DMSO) δ 7.83 - 7.82 (m, 1H), 7.53 (s, 2H), 7.12 (d, J=7.8 Hz, 1H), 6.85 (s, 1H), 6.70 - 6.69 (m, 1H), 6.17 (dd, J=5.8, 5.8 Hz, 1H), 3.64 - 3.60 (m, 6H), 3.18 (s, 3H), 2.99 – 2.95 (m, 4H), 2.39 (s, 3H); LRMS (ES^+) m/z 456 $[M+H]^+$.

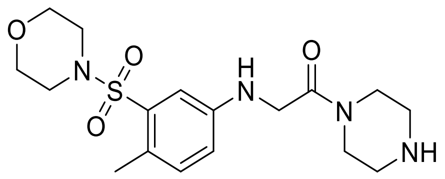

Compound 13

2-((4-methyl-3-(morpholinosulfonyl)phenyl)amino)-1-(piperazin-1-yl)ethan-1-one

A mixture of tert-butyl 4-(2-chloroacetyl)piperazine-1-carboxylate (31 mg, 0.12 mmol), potassium iodide (58 mg, 0.35 mmol) in anhydrous DMF (0.5 mL) was stirred at 45ºC for 30 mins then 4-methyl-3-morpholinosulfonyl-aniline (30 mg, 0.12 mmol) was added and the reaction mixture was stirred at 45ºC overnight. The reaction mixture was filtered and purified by mass directed HPLC, 5-95% MeCN, basic method. The residue was then dissolved in DCM and 2M HCl in dioxane (0.2 ml) was added and the reaction mixture stirred at room temperature overnight and evaporated in vacuo. The residue was dissolved in methanol and purified by SCX (2 g) eluted with MeOH/ 2M NH3/MeOH. The product obtained was freeze-dried to afford 2-((4-methyl-3-(morpholinosulfonyl)phenyl)amino)-1-(piperazin-1-yl)ethan-1-one (15 mg, 0.043 mmol, 36%)

^{1}H NMR (500 MHz, CDCl3) δ 7.15 - 7.11 (m, 2H), 6.80 - 6.77 (m, 1H), 5.14 - 5.09 (m, 1H), 3.90 (d, J=2.3 Hz, 2H), 3.76 - 3.70 (m, 6H), 3.50 (s, 2H), 3.17 (t, J=4.7 Hz, 4H), 2.98 - 2.93 (m, 4H), 2.52 (s, 3H), 1.48 - 1.28 (m, 1H); LRMS (ES^+) m/z 383 $[M+H]^+$.

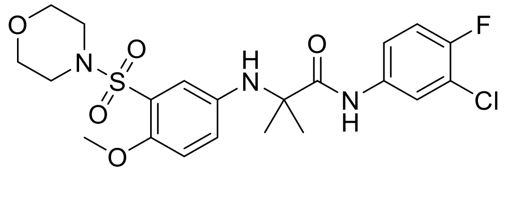

Compound 30

N-(3-chloro-4-fluorophenyl)-2-((4-methoxy-3-(morpholinosulfonyl)phenyl)amino)-2-methylpropanamide

A mixture of 4-methoxy-3-morpholinosulfonyl-aniline (70 mg, 0.26 mmol) in anhydrous THF (4 ml) was cooled to 0ºC then sodium hydride (12 mg, 0.30 mmol) was added. The reaction was stirred at 0ºC for 10mins then 2-bromo-N-(3-chloro-4-fluoro-phenyl)-2-methyl-propanamide (102

mg, 0.35 mmol) was added. The reaction mixture was stirred at rt under nitrogen for 3.5 days. The reaction mixture was partitioned between water (2 ml) and EtOAc (5 ml) and the EtOAc extract evaporated. The residue was dissolved in methanol/acetonitrile 10% water and purified by HPLC 5-95% MeCN, basic method. The product obtained was freeze-dried to afford N-(3-chloro-4-fluorophenyl)-2-((4-methoxy-3-(morpholinosulfonyl)phenyl)amino)-2-methylpropanamide (31mg, 0.06 mmol, 12 %) as a white solid.

^{1}H NMR (500 MHz, Acetone) δ 9.67 (s, 1H), 8.03 (dd, J=2.5, 6.8 Hz, 1H), 7.63 - 7.59 (m, 1H), 7.24 (dd, J=9.1, 9.1 Hz, 1H), 7.16 (d, J=3.0 Hz, 1H), 7.08 (d, J=8.8 Hz, 1H), 6.85 (dd, J=3.0, 9.0 Hz, 1H), 5.38 (s, 1H), 3.83 (s, 3H), 3.59 - 3.56 (m, 4H), 3.06 - 3.03 (m, 4H), 1.54 (s, 6H); ^{13}C NMR (126 MHz, DMSO-d_6) δ 25.0, 45.7, 56.4, 57.7, 65.7, 114.8, 116.3, 116.5 (d, J = 21.6 Hz), 118.8 (d, J = 18.2 Hz), 120.3 (d, J = 6.8 Hz), 120.5, 121.4, 124.7, 136.2 (d, J = 3.1 Hz), 139.6, 148.5, 153.1 (d, J = 242.9 Hz), 174.8; ^{19} F NMR (470.5 MHz; d^6-DMSO) δ -122.90; LRMS (ES^+) m/z 486 $[M+H]^+$.

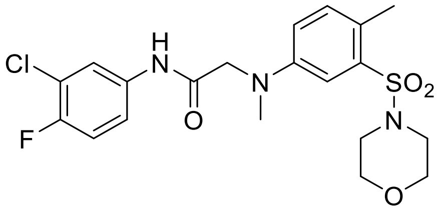

Compound 26

N-(3-chloro-4-fluorophenyl)-2-(methyl(4-methyl-3 (morpholinosulfonyl)phenyl)amino) acetamide

A mixture of N-(3-chloro-4-fluoro-phenyl)-2-(4-methyl-3-morpholinosulfonyl-anilino)acetamide (60 mg, 0.14 mmol) in chloroform chloroform (2 mL) was prepared at rt and formaldehyde (12 mg, 0.41 mmol) added and the mixture heated to 65-68ºC for 1h. The mixture was then cooled to rt and sodium triacetoxyborohydride (86 mg, 0.41 mmol) added in one portion and the mixture heated again to 65-68ºC for 16h. The mixture was then cooled to rt and diluted with DCM (10 mL) and washed with water (3 mL) and filtered through a hydrophobic fritted column. LCMS showed around 1:1 starting material to desired product so mixture was diluted in chloroform (3 mL) and paraformaldehyde (3 eq) added and the mixture heated for 14h at 65-68ºC. The mixture was then cooled to rt and sodium triacetoxyborohydride (86 mg, 0.41 mmol) added and the mixture heated again in a sealed tube to 65-68ºC for 7 hrs. The mixture was then cooled to rt, diluted with DCM (5 mL), washed with water (3 mL), concentrated under reduced pressure and purified by column chromatography (0-1% methanol/dichloromethane) to afford a colourless solid. 1HNMR in CDCl3 and LCMS indicated a mixture of starting material and product. Mixture than dissolved in methanol (2 mL) and 3 angstrom molecular sieves added followed by paraformaldehyde (3 eq) and then acetic acid (3 eq) and the mixture stirred at rt in a sealed tube for 45 mins then sodium cyanoborohydride (1M in THF) (3 eq) was added and the mixture stirred for 17 h in a sealed tube. Crude LCMS showed only desired product. Mixture diluted with 1:1 methanol/DCM (10 mL) and

filtered through a celite pad, pad washed with dcm/methanol (2 x 10 mL) and filtrate concentrated under reduced pressure. Filtrate then basified to pH 11 with 7M ammonia in methanol, concentrated under reduced pressure and purified by column (0-2% methanol/dcm) to afford the desired product (N-(3-chloro-4-fluoro-phenyl)-2-(N,4-dimethyl-3-morpholinosulfonyl-anilino)acetamide (26 mg, 0.054 mmol), 40 % yield as a colourless solid.

^{1}H NMR (500 MHz, CDCl3) δ 8.18 (s, 1H), 7.69 (dd, J=2.6, 6.4 Hz, 1H), 7.36 - 7.32 (m, 2H), 7.24 (d, J=8.5 Hz, 1H), 7.09 (t, J=9.5 Hz, 1H), 6.87 (dd, J=2.9, 8.4 Hz, 1H), 3.97 (s, 2H), 3.70 (t, J=4.7 Hz, 4H), 3.14 (t, J=4.8 Hz, 4H), 3.12 (s, 3H), 2.55 (s, 3H); ^{13}C NMR (126 MHz, DMSO-d_6) δ 19.3, 39.7, 45.3, 55.5, 65.4, 112.3, 116.6, 116.9 (d, J = 21.7 Hz), 119.1 (d, J = 18.4 Hz), 119.4 (d, J = 6.9 Hz), 120.5, 123.9, 133.6, 134.1, 135.9 (d, J = 3.0 Hz), 147.4, 153.1 (d, J = 242.8 Hz), 168.7; ^{19}F NMR (470.5 MHz; d^6-DMSO) δ -122.63; LCMS m/z 456 $(M+H)^+$

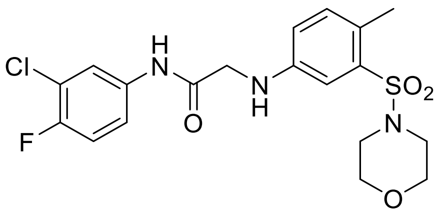

Compound 1

N-(3-chloro-4-fluorophenyl)-2-((4-methyl-(morpholinosulfonyl) phenyl) amino) acetamide

A mixture of 2-chloro-N-(3-chloro-4-fluoro-phenyl)acetamide (260 mg,1.17 mmol) in DMF (6 mL) was prepared at rt and iodopotassium (583 mg, 3.51 mmol) added and the mixture heated to 45-48ºC for 1h. The mixture was then cooled to rt and 4-methyl-3-morpholinosulfonyl-aniline (300 mg, 1.17 mmol) added in one portion and the mixture heated again to 45-48ºC in a sealed tube for 17 h. The mixture was then diluted with ethyl acetate (30 mL) and washed with 5% LiCl aq. (3 x 10 mL), brine (10 mL), dried over magnesium sulphate and the filtrate concentrated under reduced pressure and purified by column chromatography (0-5% methanol/DCM) to afford the desired product (245 mg, 0.55 mmol, 47 %) as a pale yellow solid.

^{1}H NMR (500 MHz, CDCl3) δ 8.31 (s, 1H), 7.70 (dd, J=2.7, 6.5 Hz, 1H), 7.36 - 7.32 (m, 1H), 7.25 (d, J=2.6 Hz, 1H), 7.20 (d, J=8.5 Hz, 1H), 7.09 (t, J=8.8 Hz, 1H), 6.77 (dd, J=2.7, 8.2 Hz, 1H), 4.48 (t, J=5.6 Hz, 1H), 3.94 (d, J=5.5 Hz, 2H), 3.69 (t, J=4.8 Hz, 4H), 3.13 (t, J=4.8 Hz, 4H), 2.53 (s, 3H); ^{13}C NMR (126 MHz, DMSO-d_6) δ 19.3, 45.3, 47.0, 65.4, 112.6, 116.9 (d, J = 21.7 Hz), 117.1, 119.1 (d, J = 18.3 Hz), 119.5 (d, J = 6.8 Hz), 120.6, 123.9, 133.5, 134.2, 136.0 (d, J = 2.9 Hz), 146.6, 153.1 (d, J = 242.9 Hz), 169.2; ^{19}F NMR (470.5 MHz; d^6-DMSO) δ -119.27; LCMS m/z 442 $[M+H]^+$

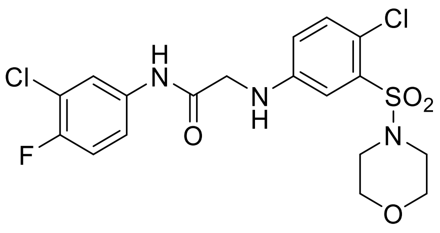

Compound 15

2-((4-chloro-3-(morpholinosulfonyl) phenyl) amino)-N-(3-chloro-4-fluorophenyl) acetamide

A mixture of 2-chloro-N-(3-chloro-4-fluoro-phenyl)acetamide (241 mg, 1.08 mmol) in DMF (6 mL) was prepared at rt and iodopotassium (540 mg, 3.25 mmol) added and the mixture heated to 45-48ºC for 1h. The mixture was then cooled to rt and 4-chloro-3-morpholinosulfonyl-aniline (300mg, 1.08 mmol) added in one portion and the mixture heated again to 45-48 ºC in a sealed tube for 17 h. The mixture was then diluted with ethyl acetate (30 mL) and washed with 5% LiCl aq. (3 x 10 mL), brine (10 mL), dried over magnesium sulphate and the filtrate concentrated under reduced pressure and purified by column chromatography (0-5% methanol/DCM) to afford the desired product N-(3-chloro-4-fluoro-phenyl)-2-(4-chloro-3-morpholinosulfonyl-anilino) acetamide (191 mg, 0.41 mmol, 38%) as a pale yellow solid.

^{1}H NMR (500 MHz, DMSO) δ 10.35 (s, 1H), 7.95 (dd, J=2.5, 6.8 Hz, 1H), 7.55 - 7.51 (m, 1H), 7.44 - 7.40 (m, 2H), 7.16 (d, J=2.8 Hz, 1H), 6.91 - 6.81 (m, 2H), 3.99 (d, J=6.1 Hz, 2H), 3.59 (t, J=4.7 Hz, 4H), 3.10 (t, J=4.7 Hz, 4H); ^{13}C NMR (126 MHz, DMSO-d_6) δ 45.6, 46.7, 65.6, 114.3, 116.4, 116.9 (d, *J* = 21.8 Hz), 117.4, 119.1 (d, *J* = 18.3 Hz), 119.5 (d, *J* = 6.8 Hz), 120.6, 132.6, 134.3, 135.9 (d, *J* = 3.1 Hz), 147.5, 153.1 (d, *J* = 242.9 Hz), 168.7; ^{19}F NMR (470.5 MHz; d^6-DMSO) δ -122.58; LCMS m/z 925 [2M+H]

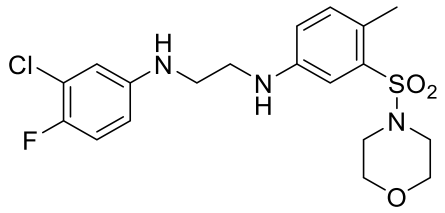

Compound 27

N1-(3-chloro-4-fluorophenyl)-N2-(4-methyl-3-morpholinosulfonyl) phenyl) ethane-1, 2-diamine

A mixture of N-(3-chloro-4-fluoro-phenyl)-2-(4-methyl-3-morpholinosulfonyl-anilino) acetamide (100 mg, 0.23 mmol) was prepared in THF (2 mL) and borane-dimethyl sulfide (2M in THF) (0.453 mL, 69 mg, 0.91 mmol) added and the mixture stirred in a sealed tube at rt for 24h. Reaction

was monitored by LCMS and completed after overnight stirring (24h). Mixture was then quenched with MeOH (5mL) and stirred for a further 24h in a sealed tube then concentrated under reduced pressure. Mixture purified by column chromatography (0-5% methanol/DCM) to afford the desired product (N'-(3-chloro-4-fluoro-phenyl)-N-(4-methyl-3-morpholinosulfonyl-phenyl) ethane-1, 2-diamine (53 mg, 0.12 mmol, 55%), as a colourless oil.

^{1}H NMR (500 MHz, CDCl3) δ 7.17 (d, J=2.6 Hz, 1H), 7.13 (d, J=8.2 Hz, 1H), 6.96 (t, J=8.8 Hz, 1H), 6.74 (dd, J=2.7, 8.2 Hz, 1H), 6.64 (dd, J=3.0, 6.0 Hz, 1H), 6.49 - 6.45 (m, 1H), 3.95 (s, 1H), 3.79 (s, 1H), 3.72 (t, J=4.7 Hz, 4H), 3.41 - 3.34 (m, 4H), 3.14 (t, J=4.8 Hz, 4H), 2.50 (s, 3H); ^{13}C NMR (126 MHz, DMSO-d_6) δ 19.2, 42.0, 42.2, 45.2, 65.5, 111.7 (d, J = 6.1 Hz), 112.1, 112.9, 116.1, 116.9 (d, J = 21.4 Hz), 119.4 (d, J = 18.2 Hz), 123.0, 133.6, 134.5, 146.2 (d, J = 1.7 Hz), 147.0, 149.0 (d, J = 233.5 Hz); ^{19} F NMR (470.5 MHz; d^6-DMSO) δ -134.06; LCMS m/z 428 $[M+H]^+$

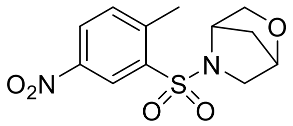

5-(2-methyl-5-nitro-phenyl)sulfonyl-2-oxa-5-azabicyclo[2.2.1]heptane

A solution of 2-methyl-5-nitro-benzenesulfonyl chloride (870 mg, 3.69 mmol) in anhydrous DCM (10 mL) was cooled in an ice bath and then triethylamine (448 mg, 4.43 mmol) and 2-oxa-5-azabicyclo[2.2.1]heptane hydrochloride (500 mg, 3.69mmol) were added. The reaction mixture was stirred in the ice bath for 30 minutes under nitrogen and then at room temperature for 3 h. The reaction was washed with water (40 mL) and the aqueous phase was extracted with DCM (40 mL). The combined organic layers were dried over $MgSO_4$ and evaporated under reduced pressure. The product was purified by column chromatography (24 g Presearch Silica cartridge) using heptane and ethyl acetate as eluents and the following gradient: 1 min hold at 100% heptane, 18 min ramp to 100% ethyl acetate, 3 min hold at 100% ethyl acetate. The fractions containing product were pooled together and solvents were removed under reduced pressure to obtain 5-(2-methyl-5-nitro-phenyl)sulfonyl-2-oxa-5-azabicyclo[2.2.1]heptane (530 mg, 1.78 mmol, 48% yield) as a white solid.

^{1}H NMR (500 MHz, $CDCl_3$) δ 8.80 (d, J = 2.4 Hz, 1H), 8.30 (dd, J=2.5, 8.4 Hz, 1H), 7.53 (d, J=8.4 Hz, 1H), 4.67 (s, 1H), 4.51 (s, 1H), 3.96 (d, J=8.0 Hz, 1H), 3.77 (dd, J=1.7, 8.0 Hz, 1H), 3.41 - 3.39 (m, 2H), 2.92 (s, 2H), 2.77 (s, 3H). LCMS m/z 299 $[M+H]^+$.

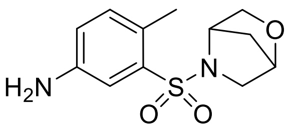

4-methyl-3-(2-oxa-5-azabicyclo[2.2.1]heptan-5-ylsulfonyl)aniline

To a solution of 5-(2-methyl-5-nitro-phenyl)sulfonyl-2-oxa-5-azabicyclo[2.2.1]heptane (530 mg, 1.78 mmol) in ethanol (20 mL), zinc chloride dihydrate (2889 mg, 12.81 mmol) was added. The reaction mixture was heated at 70 ^{o}C for 3h. Reaction was cooled down and carefully quenched with an aqueous saturated solution of $NaHCO_3$ (40 mL) and stirred for 1h at room temperature. Ethyl acetate was added (100 mL) and the mixture was filtered through Celite (10 g Celite cartridge). The organic phase was separated, dried over MgSO4 and concentrated under reduced pressure to obtain 4-methyl-3-(2-oxa-5-azabicyclo[2.2.1]heptan-5-ylsulfonyl)aniline (400 mg, 1.49 mmol, 84 % yield) as yellow solid.

^{1}H NMR (300 MHz, $CDCl_3$) δ 7.33 - 7.28 (m, 1H), 7.08 (d, J=8.1 Hz, 1H), 6.77 (dd, J=2.6, 8.1 Hz, 1H), 4.61 (s, 1H), 4.44 (d, J=1.3 Hz, 1H), 3.97 (d, J=7.7 Hz, 1H), 3.82 (s, 2H), 3.72 (dd, J=1.8, 7.8 Hz, 1H), 3.33 (s, 2H), 2.51 (s, 3H), 1.85 (s, 2H). LCMS m/z 269 $[M+H)^{+}$, 537 $[2M+H]^{+}$, 805 $[3M+H]^{+}$.

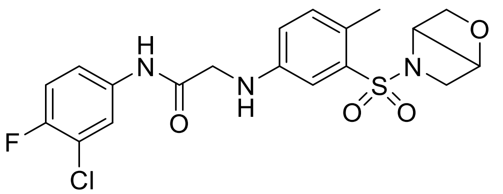

Compound 23

***N*-(3-chloro-4-fluoro-phenyl)-2-[4-methyl-3-(2-oxa-5-azabicyclo[2.2.1]heptan-5-ylsulfonyl)anilino]acetamide**

A mixture of 4-methyl-3-(2-oxa-5-azabicyclo[2.2.1]heptan-5-ylsulfonyl)aniline (85 mg, 0.32 mmol), potassium iodide (157 mg, 0.95 mmol) in anhydrous DMF (2 mL) was stirred at 45°C for 30 minutes then 2-chloro-*N*-(3-chloro-4-fluoro-phenyl)acetamide (70 mg, 0.31 mmol) was added and the reaction mixture stirred at 45°C overnight. The reaction mixtures were then filtered and purified by preparative HPLC, 5-95% MeCN, acidic conditions. The fractions containing product were pooled together and solvents were removed by centrifugation under reduced pressure (Genivac) to obtain *N*-(3-chloro-4-fluoro-phenyl)-2-[4-methyl-3-(2-oxa-5-azabicyclo[2.2.1]heptan-5-ylsulfonyl)anilino]acetamide (47 mg, 0.10 mmol, 32 % yield) as off white solid.

^{1}H NMR (500 MHz, DMSO) δ 10.26 (s, 1H), 7.92 (dd, J=2.5, 6.8 Hz, 1H), 7.51 (ddd, J=2.6, 4.3, 9.0 Hz, 1H), 7.38 (dd, J=9.1, 9.1 Hz, 1H), 7.17 - 7.11 (m, 2H), 6.79 (dd, J=2.6, 8.3 Hz, 1H), 6.41 - 6.41 (m, 1H), 4.52 (s, 1H), 4.31 (s, 1H), 3.92 (s, 2H), 3.72 (d, J=7.7 Hz, 1H), 3.57 (dd, J=1.7, 7.7 Hz, 1H), 3.17 - 3.10 (m, 2H), 2.41 (s, 3H), 1.70 - 1.67 (m, 1H), 1.54 (dd, J=2.0, 10.1 Hz, 1H); ^{13}C NMR (126 MHz, DMSO-d_6) δ 19.0, 35.8, 47.0, 55.1, 59.4, 73.0, 75.7, 112.6, 116.6, 116.9 (d, J = 21.7 Hz), 119.1 (d, J = 18.3 Hz), 119.6 (d, J = 6.8 Hz), 120.6, 123.5, 136.0 (d, J = 3.0 Hz), 136.0, 137.0, 146.6, 153.1 (d, J = 242.5 Hz), 169.2; ^{19} F NMR (470.5 MHz; d^6-DMSO) δ -122.71; LCMS m/z 454 $[M+H]^+$, 907 $[2M+H]^+$.

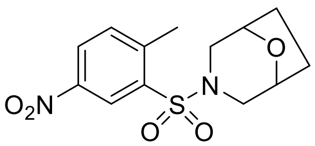

3-(2-methyl-5-nitro-phenyl)sulfonyl-8-oxa-3-azabicyclo[3.2.1]octane

A solution of 2-methyl-5-nitro-benzenesulfonyl chloride (870 mg, 3.69 mmol) in anhydrous DCM (10 mL) was cooled in an ice bath and then triethylamine (448 mg, 4.43 mmol) and 8-oxa-3-azabicyclo[3.2.1]octane (501 mg, 4.43mmol) were added. The reaction mixture was stirred in the ice bath for 30 minutes under nitrogen then at room temperature for 3 h. The reaction mixture was washed with water (40 mL) and the aqueous phase extracted with DCM (40 mL). The combined organic layers were evaporated under reduced pressure to obtain 3-(2-methyl-5-nitro-phenyl)sulfonyl-8-oxa-3-azabicyclo[3.2.1]octane (1091 mg, 3.31 mmol, 90% yield) as a pale yellow solid.

^{1}H NMR (500 MHz, $CDCl_3$) δ 8.57 (d, J=2.2 Hz, 1H), 8.22 (dd, J=2.4, 8.4 Hz, 1H), 7.46 (d, J=8.4 Hz, 1H), 4.34 (s, 2H), 3.37 (d, J=11.5 Hz, 2H), 2.96 - 2.92 (m, 2H), 2.68 (s, 3H), 1.91 (s, 4H). LCMS m/z 313 $[M+H]^+$

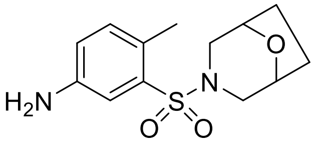

4-methyl-3-(8-oxa-3-azabicyclo[3.2.1]octan-3-ylsulfonyl)aniline

To a solution of the aromatic nitro compound (3-(2-methyl-5-nitro-phenyl)sulfonyl-8-oxa-3-azabicyclo[3.2.1]octane (1000 mg, 3.20 mmol) in ethanol (20mL), zinc chloride dihydrate (2889 mg, 12.81mmol) was added. The reaction mixture was heated at 70 °C for 3h. Reaction was cooled

down and carefully quenched with an aqueous saturated solution of $NaHCO_3$ (40 mL) and stirred for 1h at room temperature. Ethyl acetate was added (100 mL) and the mixture was filtered through Celite (10 g Celite cartridge). The organic phase was separated, dried over MgSO4 and concentrated under reduced pressure to obtain 4-methyl-3-(8-oxa-3-azabicyclo[3.2.1]octan-3-ylsulfonyl)aniline (900 mg, 3.19 mmol, 99% yield).

^{1}H NMR (300 MHz, $CDCl_3$) δ 7.07 (1H, d, J=2.4 Hz), 7.00 (1H, d, J=8.1 Hz), 6.69 (1H, dd, J=2.6, 8.1 Hz), 4.29 (2H, s), 3.70 (2H, s), 3.27 - 3.21 (2H, m), 2.87 - 2.81 (2H, m), 2.42 (3H, s), 1.98 - 1.84 (2H, m). LCMS m/z 283 $[M+H]^+$, 565 $[2M+H]^+$, 847 $[3M+H]^+$.

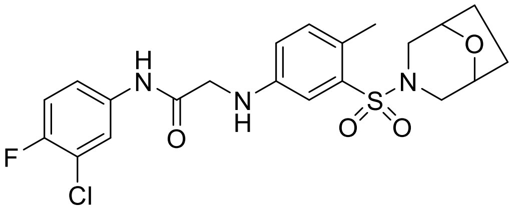

Compound 24

***N*-(3-chloro-4-fluoro-phenyl)-2-[4-methyl-3-(8-oxa-3-azabicyclo[3.2.1]octan-3-ylsulfonyl)anilino]acetamide**

A mixture of the corresponding aniline (4-methyl-3-(8-oxa-3-azabicyclo[3.2.1]octan-3-ylsulfonyl)aniline (89 mg, 0.31 mmol), potassium iodide (157 mg, 0.95 mmol) in anhydrous DMF (2 mL) was stirred at 45°C for 30 minutes then 2-chloro-*N*-(3-chloro-4-fluoro-phenyl)acetamide (70 mg, 0.32 mmol) was added and the reaction mixture stirred at 45ºC overnight. The reaction mixtures were then filtered and purified by Gilson HPLC, 5-95% MeCN, acidic conditions. The fractions containing product were pooled together and solvents were removed by centrifugation under reduced pressure (Genivac) to obtain *N*-(3-chloro-4-fluoro-phenyl)-2-[4-methyl-3-(8-oxa-3-azabicyclo[3.2.1]octan-3-ylsulfonyl)anilino]acetamide (40 mg, 0.08mmol, 27% yield) as yellow solid.

^{1}H NMR (500 MHz, DMSO) δ 10.40 (s, 1H), 8.05 (dd, J=2.5, 6.8 Hz, 1H), 7.63 (ddd, J=2.6, 4.3, 9.0 Hz, 1H), 7.50 (dd, J=9.1, 9.1 Hz, 1H), 7.27 (d, J=8.4 Hz, 1H), 7.04 (d, J=2.5 Hz, 1H), 6.92 (dd, J=2.6, 8.3 Hz, 1H), 6.57 (dd, J=6.1, 6.1 Hz, 1H), 4.33 (d, J=1.9 Hz, 2H), 4.03 (d, J=6.1 Hz, 2H), 3.26 (d, J=11.3 Hz, 2H), 2.78 (dd, J=1.9, 11.5 Hz, 2H), 2.63 - 2.61 (m, 3H), 1.91 - 1.81 (m, 4H); ^{13}C NMR (126 MHz, DMSO-d_6) δ 19.3, 27.2, 47.0, 50.4, 72.8, 112.4, 116.9, 116.9 (d, J = 21.7 Hz), 119.1 (d, J = 18.3 Hz), 119.5 (d, J = 6.7 Hz), 120.6, 123.7, 133.4, 134.8, 136.0 (d, J = 3.1 Hz), 146.6, 153.1 (d, J = 242.7 Hz), 169.2; ^{19}F NMR (470.5 MHz; d^6-DMSO) δ -122.70; LCMS m/z 468 $[M+H]^+$, 935 $[2M+H]^+$.

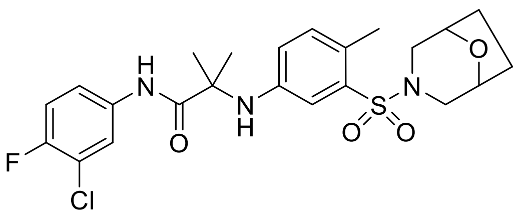

Compound 29

***N*-(3-chloro-4-fluoro-phenyl)-2-methyl-2-[4-methyl-3-(8-oxa-3-azabicyclo[3.2.1]octan-3-ylsulfonyl)anilino]propanamide**

A solution of (4-methyl-3-(8-oxa-3-azabicyclo[3.2.1]octan-3-ylsulfonyl)aniline (71.889mg,0.2546mmol) in anhydrous THF (2 mL) was added sodium hydride (12 mg, 0.29 mmol) and stirred for 10 minutes under nitrogen. 2-bromo-*N*-(3-chloro-4-fluoro-phenyl)-2-methyl-propanamide (100 mg, 0.34 mmol) was added and the reaction mixture stirred at room temperature under nitrogen overnight. The reaction mixtures were quenched with water (5 mL) and extracted with DCM (10 mL). The organic phase was separated, dried over $MgSO_4$ and the solvent was evaporated under reduced pressure. The residues were dissolved in acetonitrile and purified by preparative HPLC 5-95% MeCN, acidic. The fractions containing product were pooled together and the concentrated to dryness under reduced pressure (Genivac) to obtain *N*-(3-chloro-4-fluoro-phenyl)-2-methyl-2-[4-methyl-3-(8-oxa-3-azabicyclo[3.2.1]octan-3-ylsulfonyl)anilino]propanamide (40 mg, 0.08 mmol, 23% yield) as a white solid.

^{1}H NMR (500 MHz, DMSO) δ 10.01 (s, 1H), 7.97 (dd, J=2.6, 6.9 Hz, 1H), 7.63 (ddd, J=2.6, 4.3, 9.1 Hz, 1H), 7.34 (dd, J=9.1, 9.1 Hz, 1H), 7.14 (d, J=8.4 Hz, 1H), 6.89 (d, J=2.7 Hz, 1H), 6.74 (dd, J=2.6, 8.3 Hz, 1H), 6.27 (s, 1H), 4.14 (d, J=2.0 Hz, 2H), 3.09 (d, J=11.3 Hz, 2H), 2.59 (dd, J=1.7, 11.5 Hz, 2H), 2.36 (s, 3H), 1.78 - 1.68 (m, 4H), 1.46 (s, 6H); ^{13}C NMR (126 MHz, DMSO-d_6) δ 19.2, 25.0, 27.2, 50.4, 57.4, 72.8, 114.1, 116.5 (d, J = 21.5 Hz), 118.7, 118.9, 120.2 (d, J = 6.8 Hz), 121.4, 124.1, 133.2, 134.8, 136.2 (d, J = 3.1 Hz), 144.2, 153.1 (d, J = 242.8 Hz), 174.6; ^{19}F NMR (470.5 MHz; d^6-DMSO) δ -122.88; LCMS m/z 496 $[M+H]^+$, 991 $[2M+H]^+$

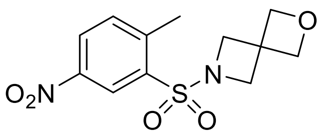

6-(2-methyl-5-nitro-phenyl)sulfonyl-2-oxa-6-azaspiro[3.3]heptane

A solution of 2-methyl-5-nitro-benzenesulfonyl chloride (556 mg, 2.36 mmol) in anhydrous DCM (10 mL) was cooled in an ice bath and then triethylamine (525 mg, 5.19 mmol) and 2-oxa-6-azaspiro[3.3]heptane oxalic acid (536 mg, 2.83 mmol) were added. The reaction mixture was stirred in the ice bath for 30 minutes under nitrogen then at room temperature for 3 h. The reaction

mixture was washed with water (40 mL) and the aqueous layer extracted with DCM (40 mL). The combined organic layers were dried over $MgSO_4$ and evaporated under reduced pressure. The product was purified by column chromatography (24 g Presearch Silica cartridge) using heptane and ethyl acetate as eluents and the following gradient: 1 min hold at 100% heptane, 18 min ramp to 100% ethyl acetate, 3 min hold at 100% ethyl acetate. The fractions containing product were pooled together and solvents were removed under reduced pressure to obtain 6-(2-methyl-5-nitro-phenyl)sulfonyl-2-oxa-6-azaspiro[3.3]heptane (285 mg, 0.94 mmol, 40% yield) as a white solid.

^{1}H NMR (500 MHz, $CDCl_3$) δ 8.79 (d, J=2.4 Hz, 1H), 8.34 (dd, J=2.5, 8.4 Hz, 1H), 7.54 (d, J=8.4 Hz, 1H), 4.80 (s, 4H), 4.16 (s, 4H), 2.75 (s, 3H). LCMS m/z 299 $[M+H]^+$.

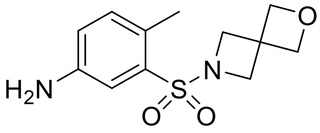

4-methyl-3-(2-oxa-6-azaspiro[3.3]heptan-6-ylsulfonyl)aniline

To a solution of 6-(2-methyl-5-nitro-phenyl)sulfonyl-2-oxa-6-azaspiro[3.3]heptane (228 mg, 0.76 mmol) in ethanol (10 mL), 10% Pd on C (45 mg, 0.76 mmol) was added. The reaction mixture was evacuated and back-filled with nitrogen three times. The reaction was then evacuated and back-filled with hydrogen using a hydrogen balloon. The reaction was stirred under hydrogen at room temperature overnight and then the mixture was filtered through Celite (10 g Celite cartridge). Solvents were removed under reduce pressure and the product was purified by column chromatography (12 g Presearch Silica cartridge) using heptane and ethyl acetate as eluents and the following gradient: 1 min hold at 100% heptane, 15 min ramp to 100% ethyl acetate, 3 min hold at 100% ethyl acetate. The fractions containing product were pooled together and solvents were removed under reduced pressure to obtain 4-methyl-3-(2-oxa-6-azaspiro[3.3]heptan-6-ylsulfonyl)aniline (75 mg, 0.27 mmol, 36% yield) as a white solid.

^{1}H NMR (500 MHz, $CDCl_3$) δ 7.29 - 7.26 (m, 1H), 7.11 (d, J=8.0 Hz, 1H), 6.80 (dd, J=2.5, 8.0 Hz, 1H), 4.74 (s, 4H), 4.03 (s, 4H), 3.78 (s, 2H), 2.50 (s, 3H). LCMS m/z 269 $[M+H]^+$, 537 $[2M+H]^+$

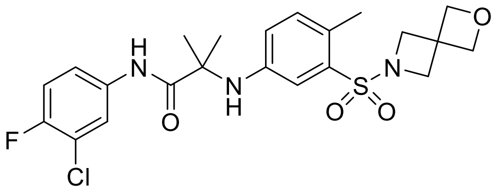

Compound 31

***N*-(3-chloro-4-fluoro-phenyl)-2-methyl-2-[4-methyl-3-(2-oxa-6-azaspiro[3.3]heptan-6-ylsulfonyl)anilino]propanamide**

A solution of 4-methyl-3-(2-oxa-6-azaspiro[3.3]heptan-6-ylsulfonyl)aniline (34 mg, 0.13 mmol) in anhydrous THF (2 mL) was added sodium hydride (6 mg,0.14 mmol) and stirred for 10 minutes under nitrogen. 2-bromo-*N*-(3-chloro-4-fluoro-phenyl)-2-methyl-propanamide (50 mg, 0.17 mmol) was added and the reaction mixture stirred at room temperature under nitrogen overnight. The reaction mixtures were quenched with water (5 mL) and extracted with DCM (10 mL). The organic phase was separated, dried over $MgSO_4$ and the solvent was remove under reduced pressure. The residues were dissolved in methanol and purified by mass directed auto-preparative HPLC 5-95% MeCN, acidic. The fractions containing product were pooled together and the concentrated to dryness under reduced pressure (Genivac) to obtain *N*-(3-chloro-4-fluoro-phenyl)-2-methyl-2-[4-methyl-3-(2-oxa-6-azaspiro[3.3]heptan-6-ylsulfonyl)anilino]propanamide (10 mg, 0.02 mmol, 12% yield) as white solid.

^{1}H NMR (500 MHz, DMSO) δ 10.08 (s, 1H), 7.95 (dd, J=2.5, 6.9 Hz, 1H), 7.63 - 7.59 (m, 1H), 7.35 (dd, J=9.1, 9.1 Hz, 1H), 7.14 (d, J=8.4 Hz, 1H), 7.04 (d, J=2.5 Hz, 1H), 6.69 (dd, J=2.6, 8.3 Hz, 1H), 6.36 (s, 1H), 4.48 (s, 4H), 3.78 (s, 4H), 2.34 (s, 3H), 1.46 (s, 6H); ^{13}C NMR (126 MHz, DMSO-d_6) δ 19.1, 25.0, 36.7, 57.4, 58.3, 78.9, 114.8, 116.5 (d, J = 21.5 Hz), 118.7, 118.9, 120.4 (d, J = 6.7 Hz), 121.5, 124.6, 133.1, 134.0, 136.2 (d, J = 3.0 Hz), 144.2, 153.2 (d, J = 243.0 Hz), 174.6; ^{19} F NMR (470.5 MHz; d^{6}-DMSO) δ -122.78; LCMS m/z 482 $[M+H]^+$, 963 $[2M+H]^+$.

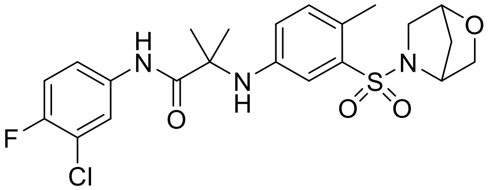

Compound 32

2-((3-((2-oxa-5-azabicyclo[2.2.1]heptan-5-yl)sulfonyl)-4-methylphenyl)amino)-N-(3-chloro-4-fluorophenyl)-2-methylpropanamide

^{1}H NMR (500 MHz, DMSO) δ 10.00 (s, 1H), 7.93 (dd, J = 2.6, 6.9 Hz, 1H), 7.60 - 7.56 (m, 1H), 7.32 (t, J = 9.1 Hz, 1H), 7.12 (d, J = 8.5 Hz, 1H), 7.09 (d, J = 2.5 Hz, 1H), 6.68 (dd, J = 2.6, 8.3 Hz, 1H), 6.26 (s, 1H), 4.43 (s, 1H), 4.19 (s, 1H), 3.68 (d, J = 7.7 Hz, 1H), 3.51 (dd, J = 1.7, 7.6 Hz, 1H), 3.04 (s, 2H), 2.37 (s, 3H), 1.62 (dd, J = 2.6, 10.0 Hz, 1H), 1.50 (dd, J = 1.9, 10.1 Hz, 1H), 1.45 (s, 6H); ^{13}C NMR (126 MHz, DMSO-d_6) δ 18.9, 25.0, 35.7, 55.0, 57.4, 59.3, 73.0, 75.6, 114.5, 116.5 (d, J = 21.6 Hz), 118.5, 118.8 (d, J = 18.3 Hz), 120.3 (d, J = 6.8 Hz), 121.5, 124.0, 133.2, 136.2 (d, J = 3.0 Hz), 136.9, 144.3, 153.1 (d, J = 242.9 Hz), 174.6; ^{19} F NMR (470.5 MHz; d^6-DMSO) δ -122.83; LCMS m/z 482 $[M+H]^+$, 963 $[2M+H]^+$.

References

1. Norcross, N. R.; Baragana, B.; Wilson, C.; Hallyburton, I.; Osuna-Cabello, M.; Norval, S.; Riley, J.; Stojanovski, L.; Simeons, F. R. C.; Porzelle, A.; Grimaldi, R.; Wittlin, S.; Duffy, S.; Avery, V. M.; Meister, S.; Sanz, L.; Jimenez-Diaz, B.; Angulo-Barturen, I.; Ferrer, S.; Martinez, M. S.; Gamo, F. J.; Frearson, J. A.; Gray, D. W.; Fairlamb, A. H.; Winzeler, E. A.; Waterson, D.; Campbell, S. F.; Willis, P.; Read, K. D.; Gilbert, I. H. Trisubstituted Pyrimidines as Efficacious and Fast-Acting Antimalarials. *J. Med. Chem.* **2016,** 59, 6101-6120.
2. Baragana, B.; Hallyburton, I.; Lee, M. C. S.; Norcross, N. R.; Grimaldi, R.; Otto, T. D.; Proto, W. R.; Blagborough, A. M.; Meister, S.; Wirjanata, G.; Ruecker, A.; Upton, L. M.; Abraham, T. S.; Almeida, M. J.; Pradhan, A.; Porzelle, A.; Martinez, M. S.; Bolscher, J. M.; Woodland, A.; Norval, S.; Zuccotto, F.; Thomas, J.; Simeons, F.; Stojanovski, L.; Osuna-Cabello, M.; Brock, P. M.; Churcher, T. S.; Sala, K. A.; Zakutansky, S. E.; Jimenez-Diaz, M. B.; Sanz, L. M.; Riley, J.; Basak, R.; Campbell, M.; Avery, V. M.; Sauerwein, R. W.; Dechering, K. J.; Noviyanti, R.; Campo, B.; Frearson, J. A.; Angulo-Barturen, I.; Ferrer-Bazaga, S.; Gamo, F. J.; Wyatt, P. G.; Leroy, D.; Siegl, P.; Delves, M. J.; Kyle, D. E.; Wittlin, S.; Marfurt, J.; Price, R. N.; Sinden, R. E.; Winzeler, E. A.; Charman, S. A.; Bebrevska, L.; Gray, D. W.; Campbell, S.; Fairlamb, A. H.; Willis, P. A.; Rayner, J. C.; Fidock, D. A.; Read, K. D.; Gilbert, I. H. A novel multiple-stage antimalarial agent that inhibits protein synthesis. *Nature* **2015,** 522, 315-320.

Compound 1

PROTON.d CDCl3 {C:\Bruker\TOPSPIN} IG500 25

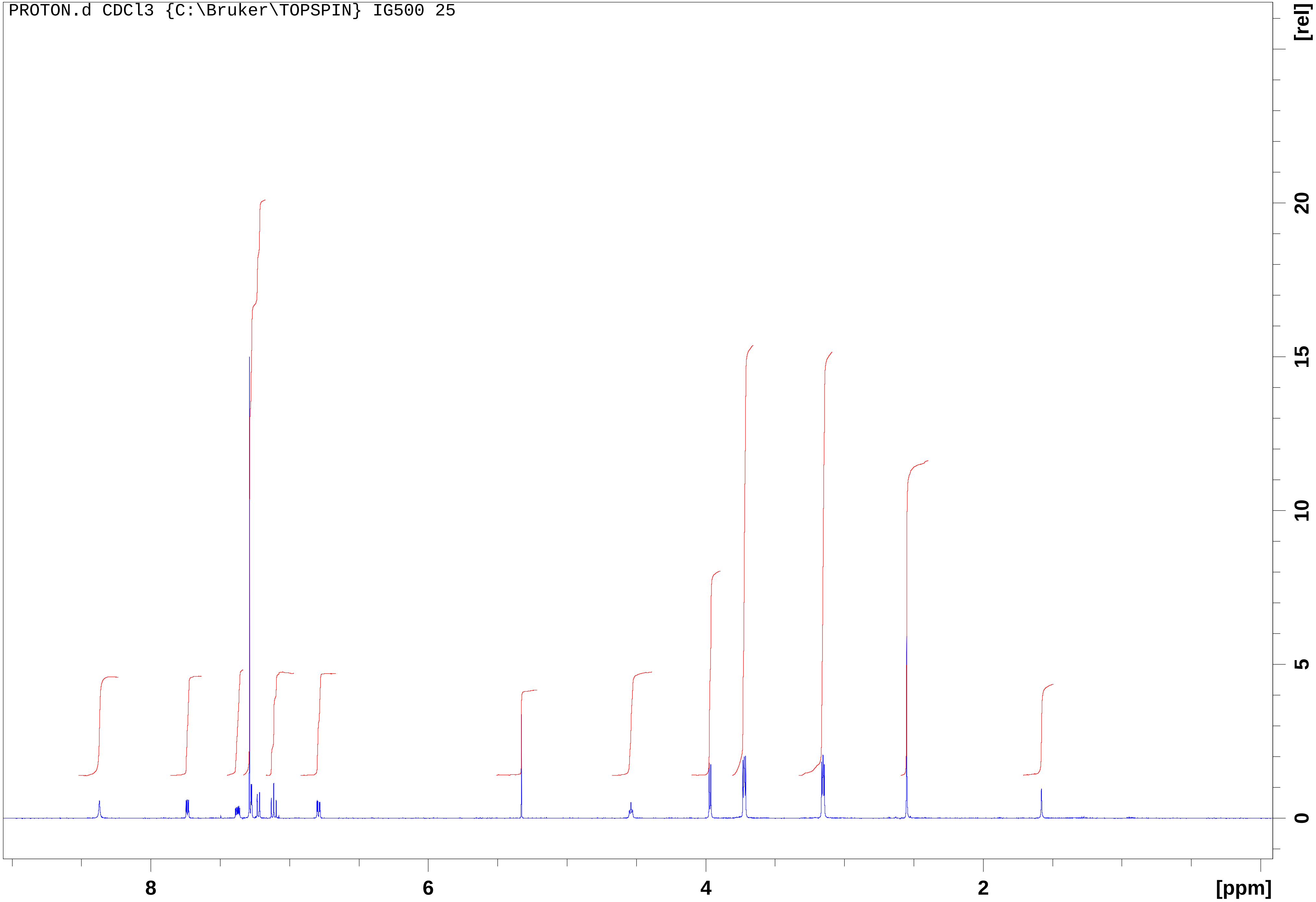

Compound 2

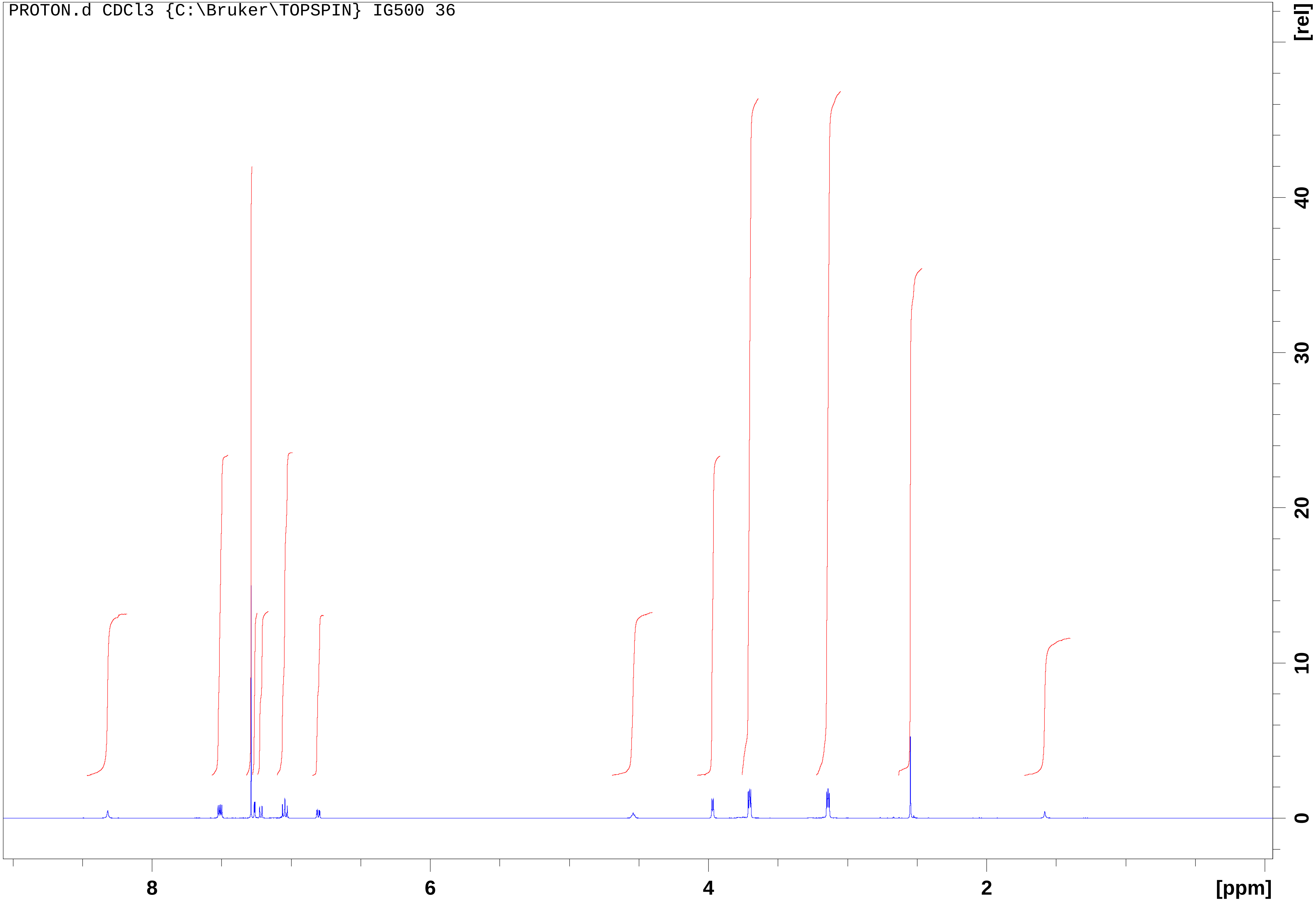
PROTON.d CDCl3 {C:\Bruker\TOPSPIN} IG500 36
[rel]
0
10
20
30
40
8
6
4
2
[ppm]

Compound 3

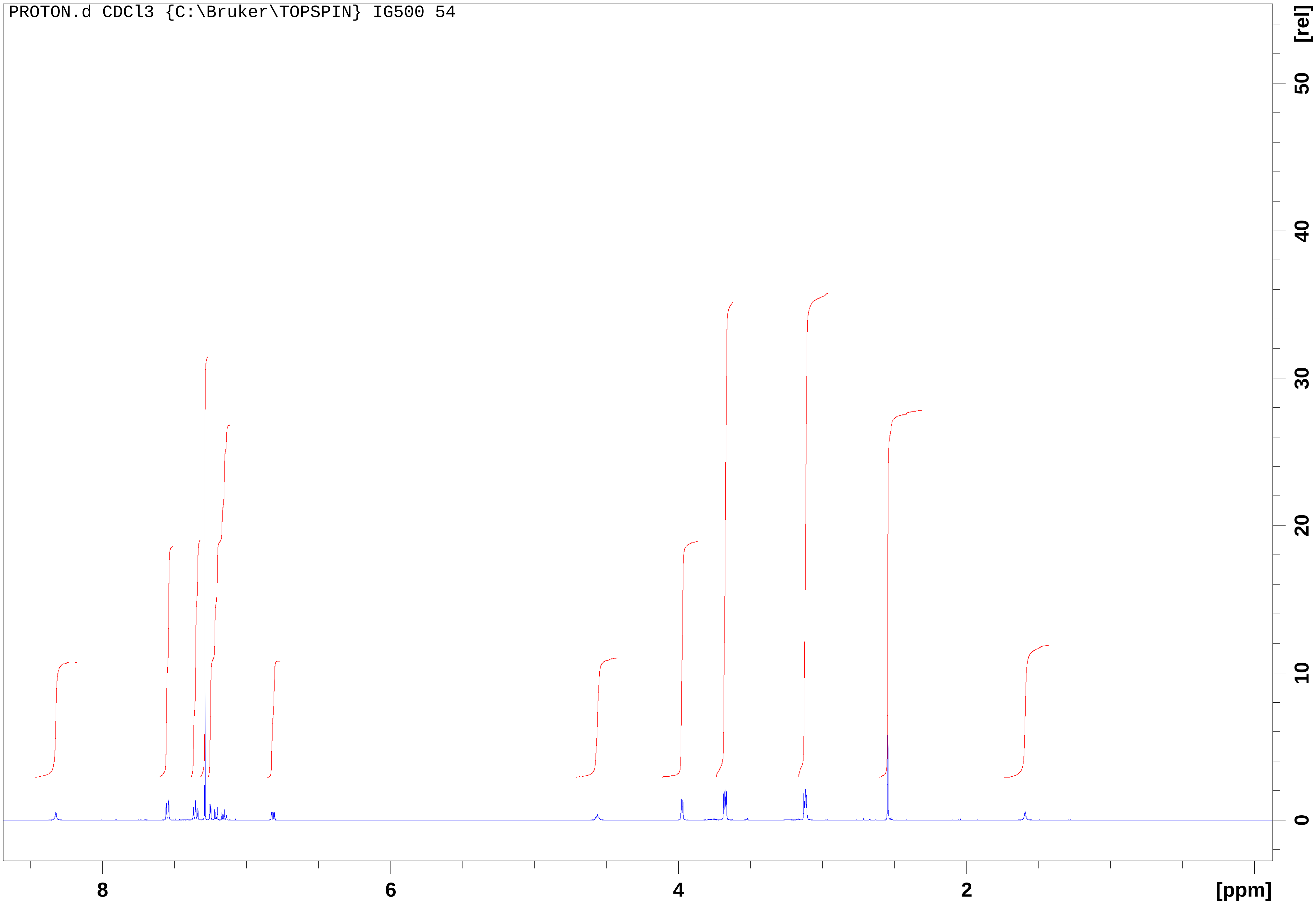
PROTON.d CDCl3 {C:\Bruker\TOPSPIN} IG500 54
[rel]
50
40
30
20
10
0
8
6
4
2
[ppm]

Compound 4

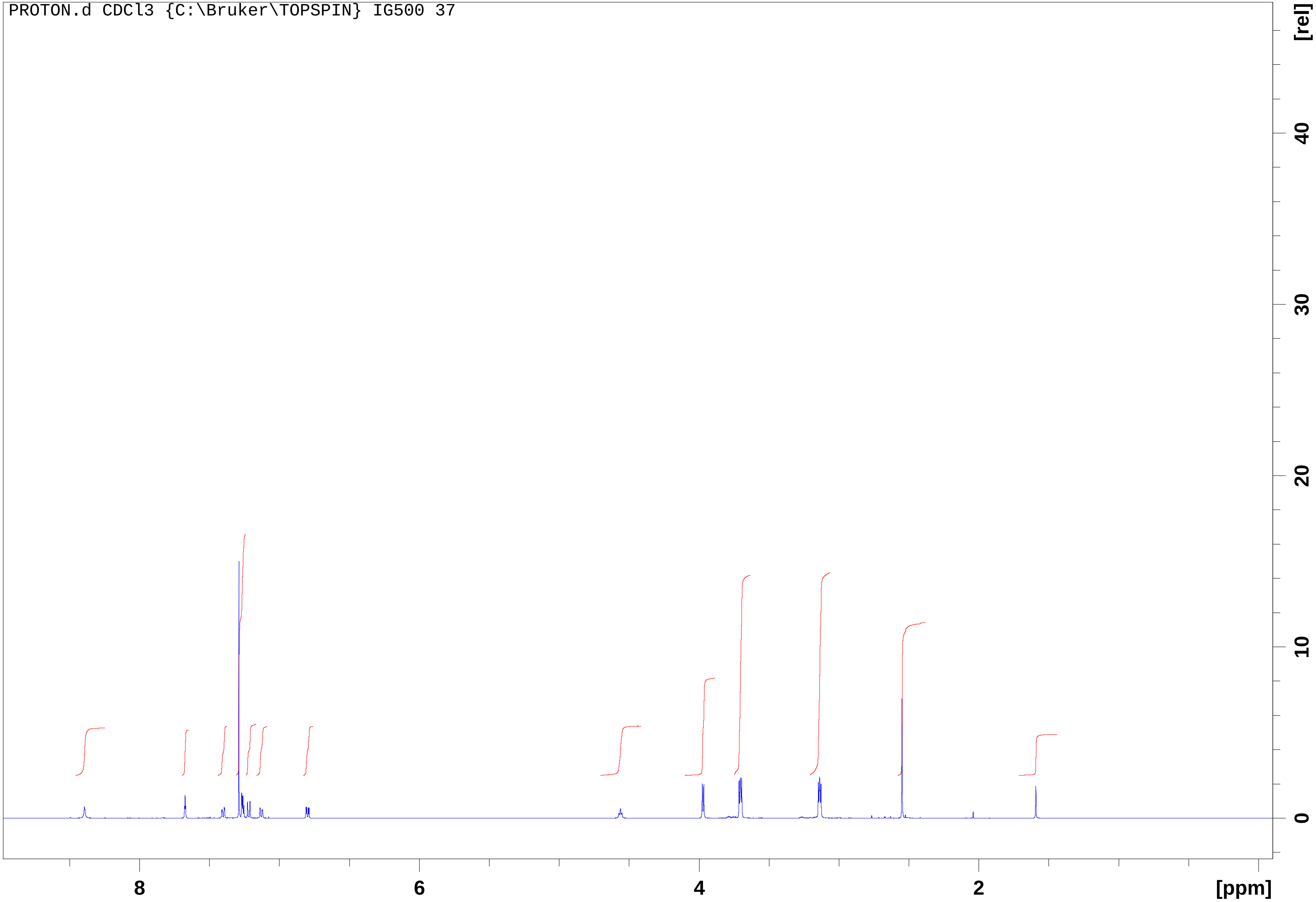

PROTON.d CDCl3 {C:\Bruker\TOPSPIN} IG500 37
[rel]
40
30
20
10
0
8
6
4
2
[ppm]

Compound 5

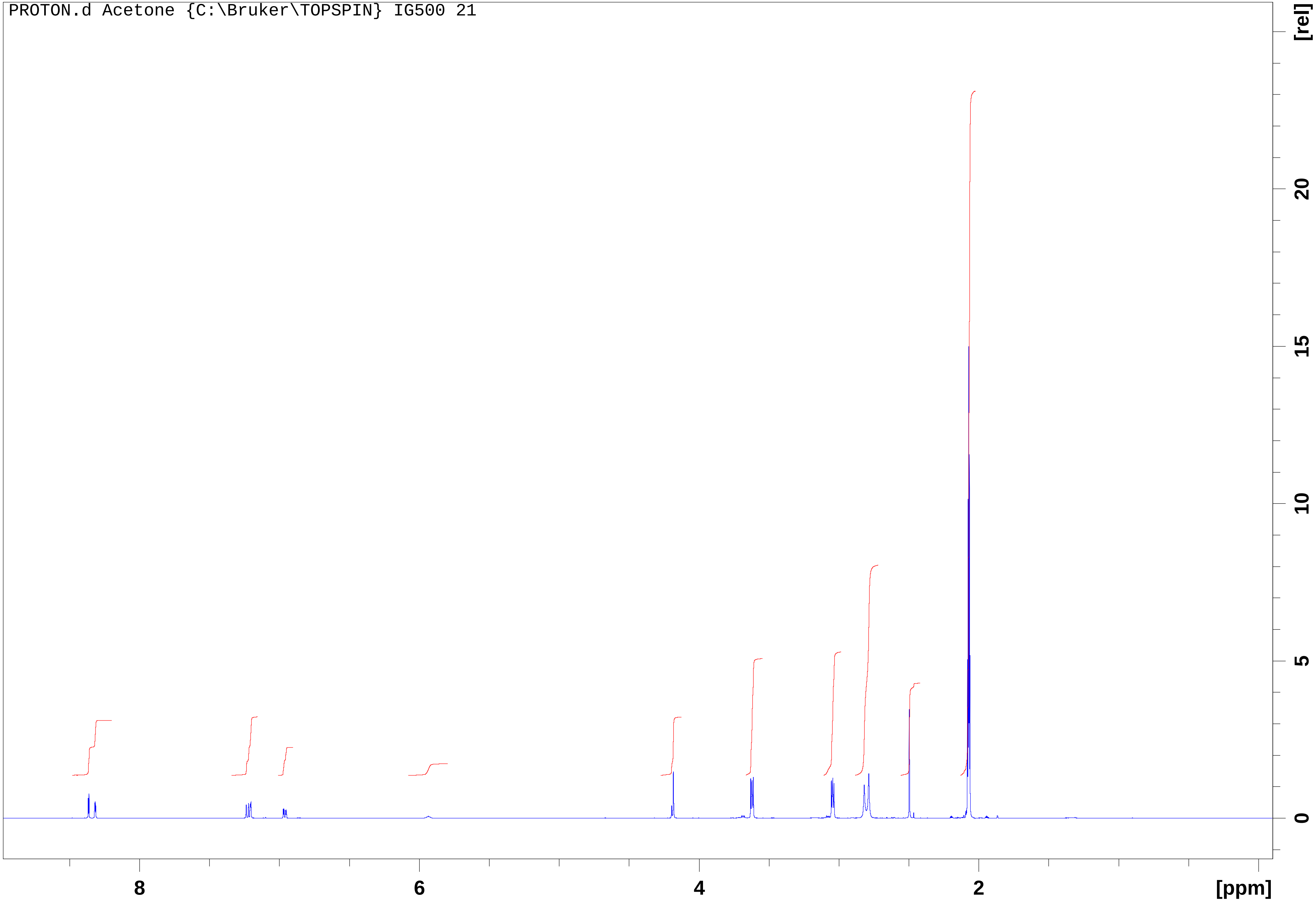
PROTON.d Acetone {C:\Bruker\TOPSPIN} IG500 21
[rel]
20
15
10
5
0
8
6
4
2
[ppm]

Compound 6

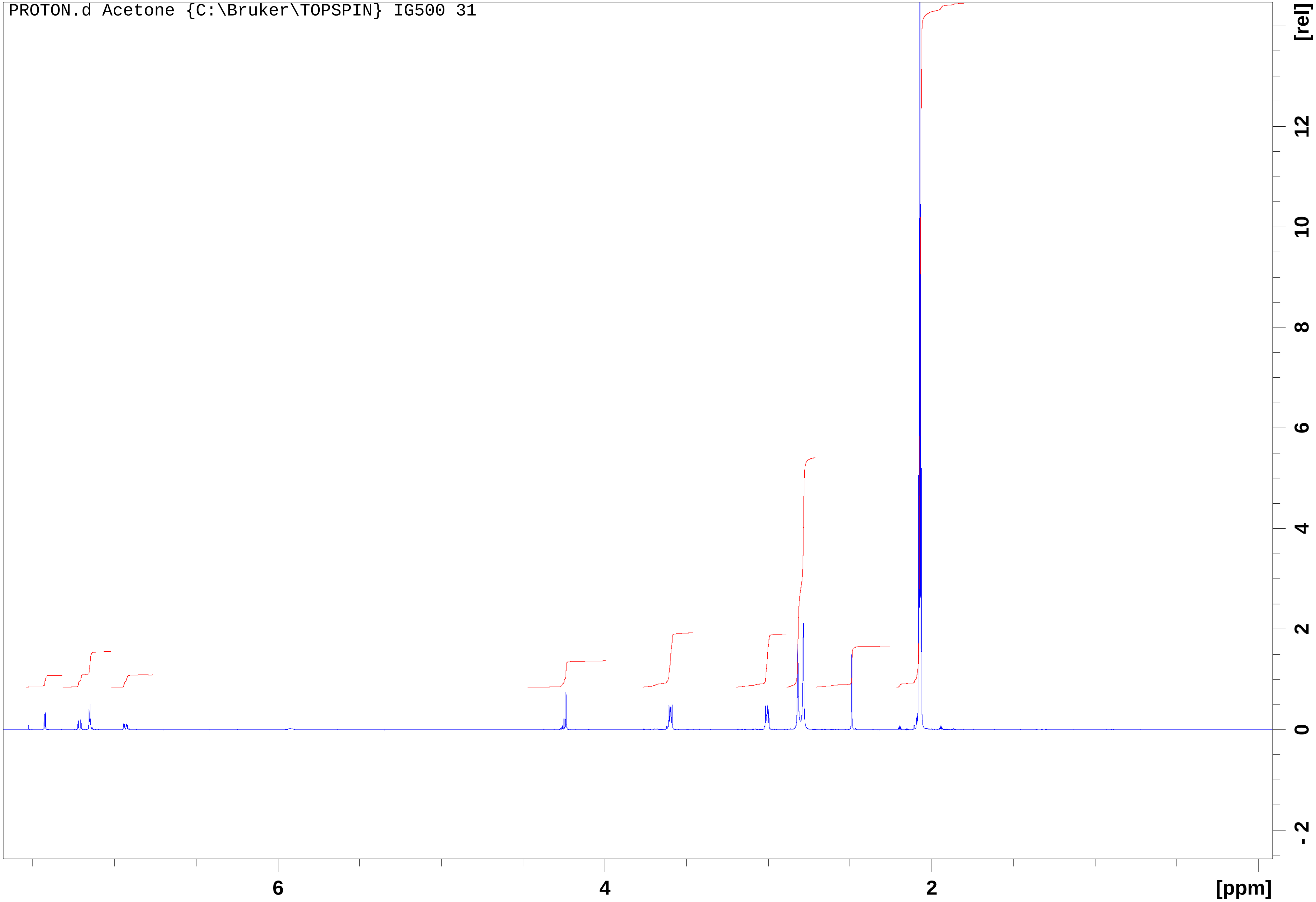

PROTON.d Acetone {C:\Bruker\TOPSPIN} IG500 31
[rel]
12
10
8
6
4
2
0
- 2
6
4
2
[ppm]

Compound 7
PROTON.d Acetone {C:\Bruker\TOPSPIN} IG500 30

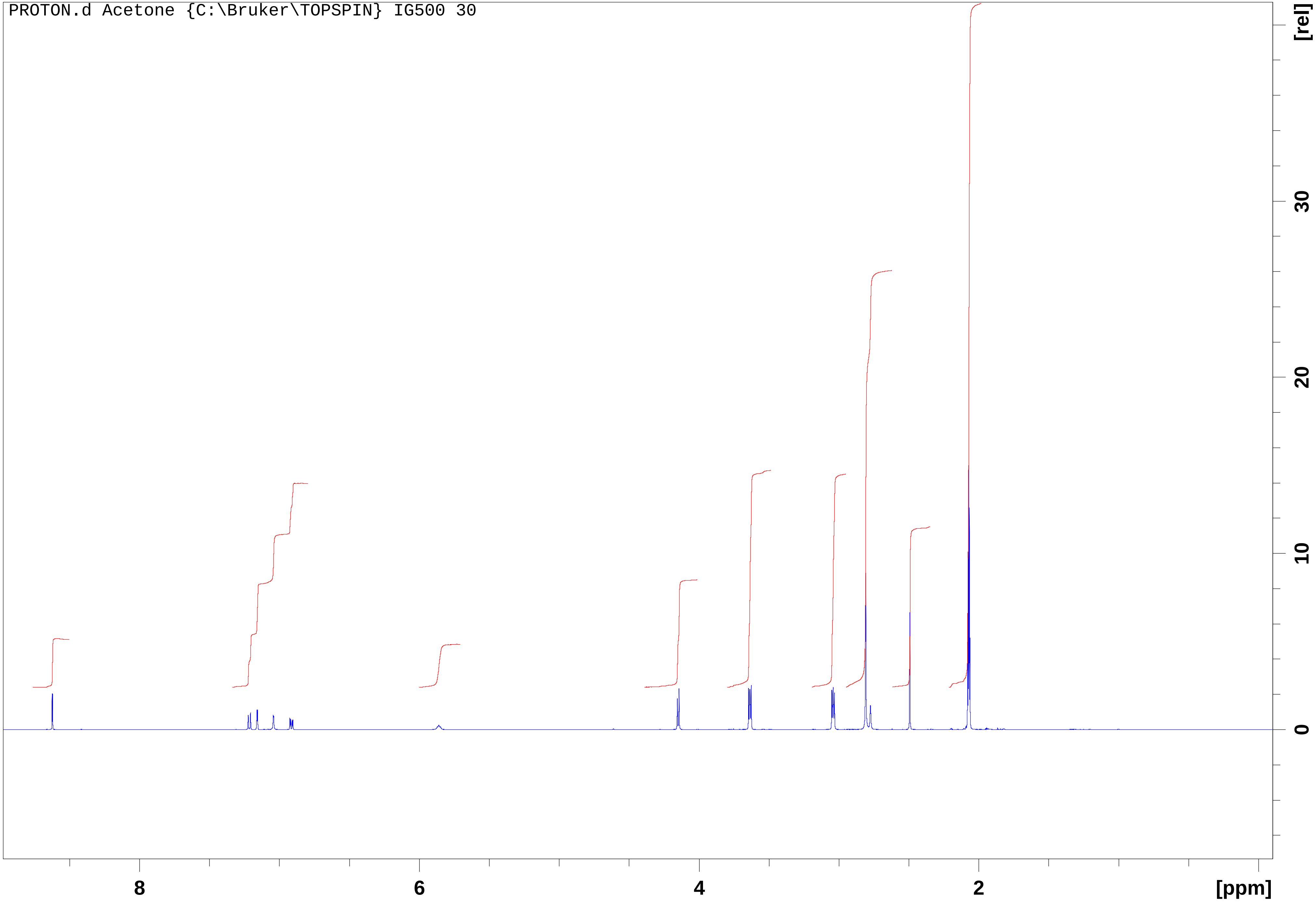

Compound 8

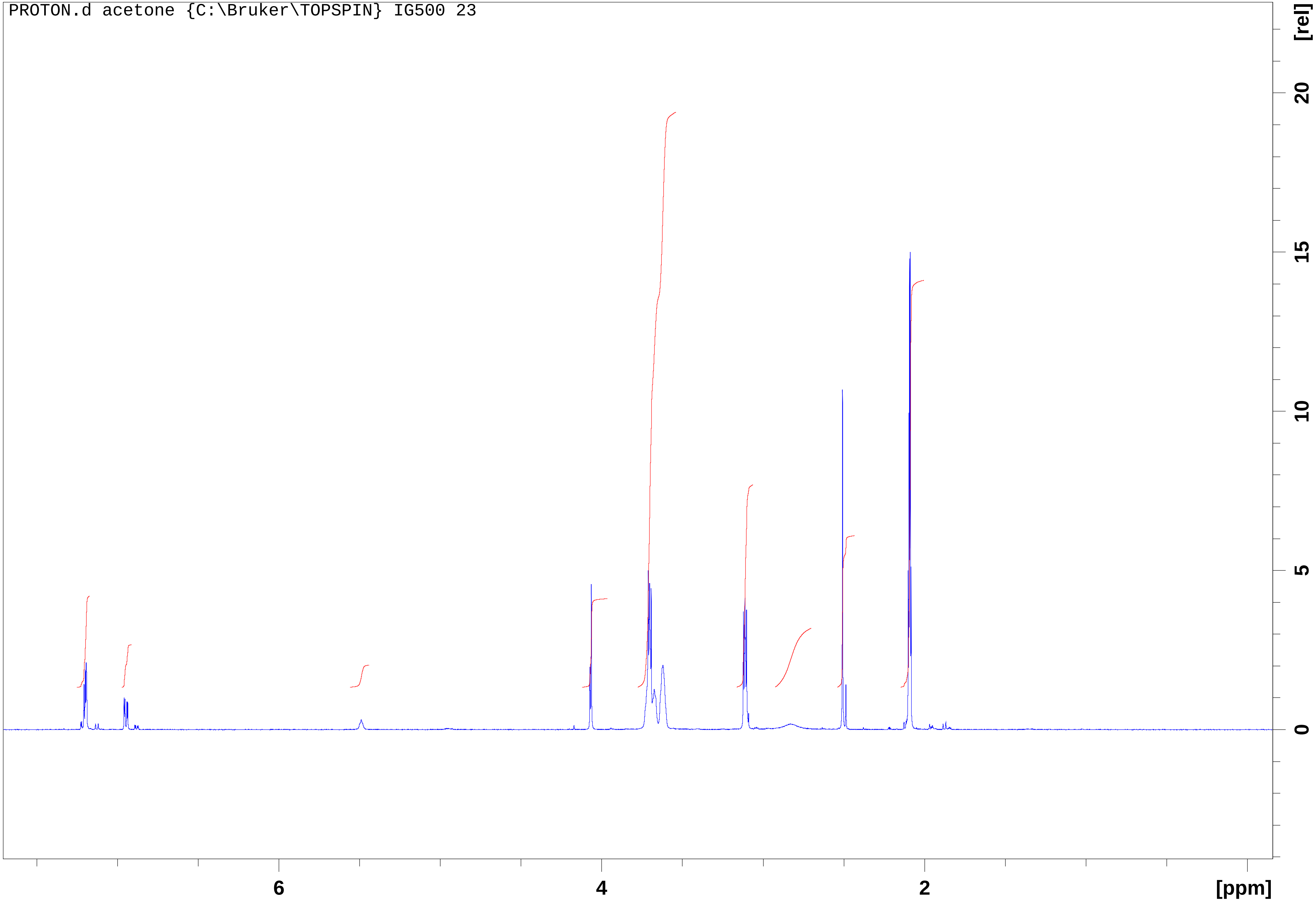

PROTON.d acetone {C:\Bruker\TOPSPIN} IG500 23
[rel]
20
15
10
5
0
6
4
2
[ppm]

Compound 9

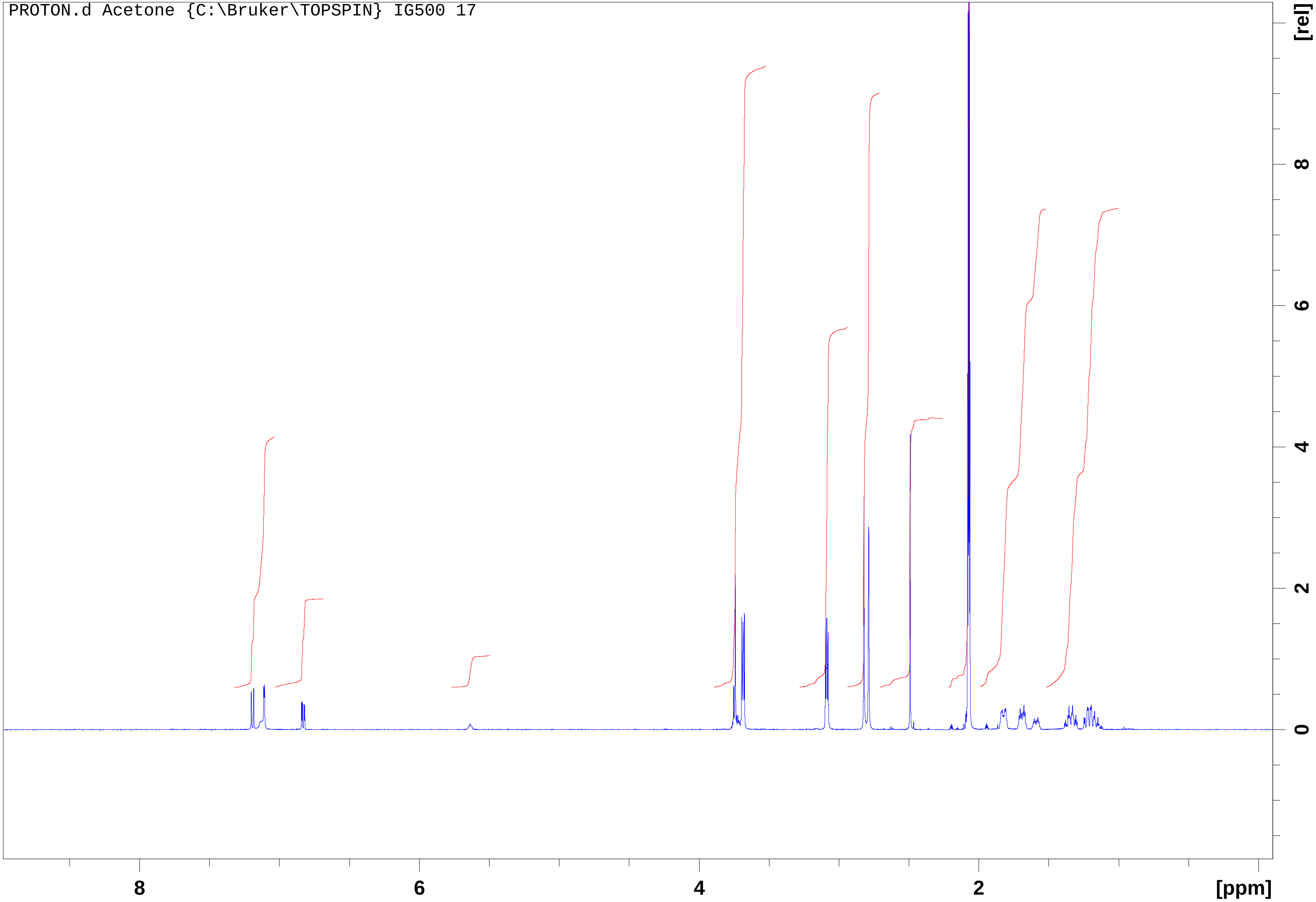

PROTON.d Acetone {C:\Bruker\TOPSPIN} IG500 17
[rel]
8
6
4
2
0
8
6
4
2
[ppm]

Compound 10 PROTON.d Acetone {C:\Bruker
\TOPSPIN} IG500 6

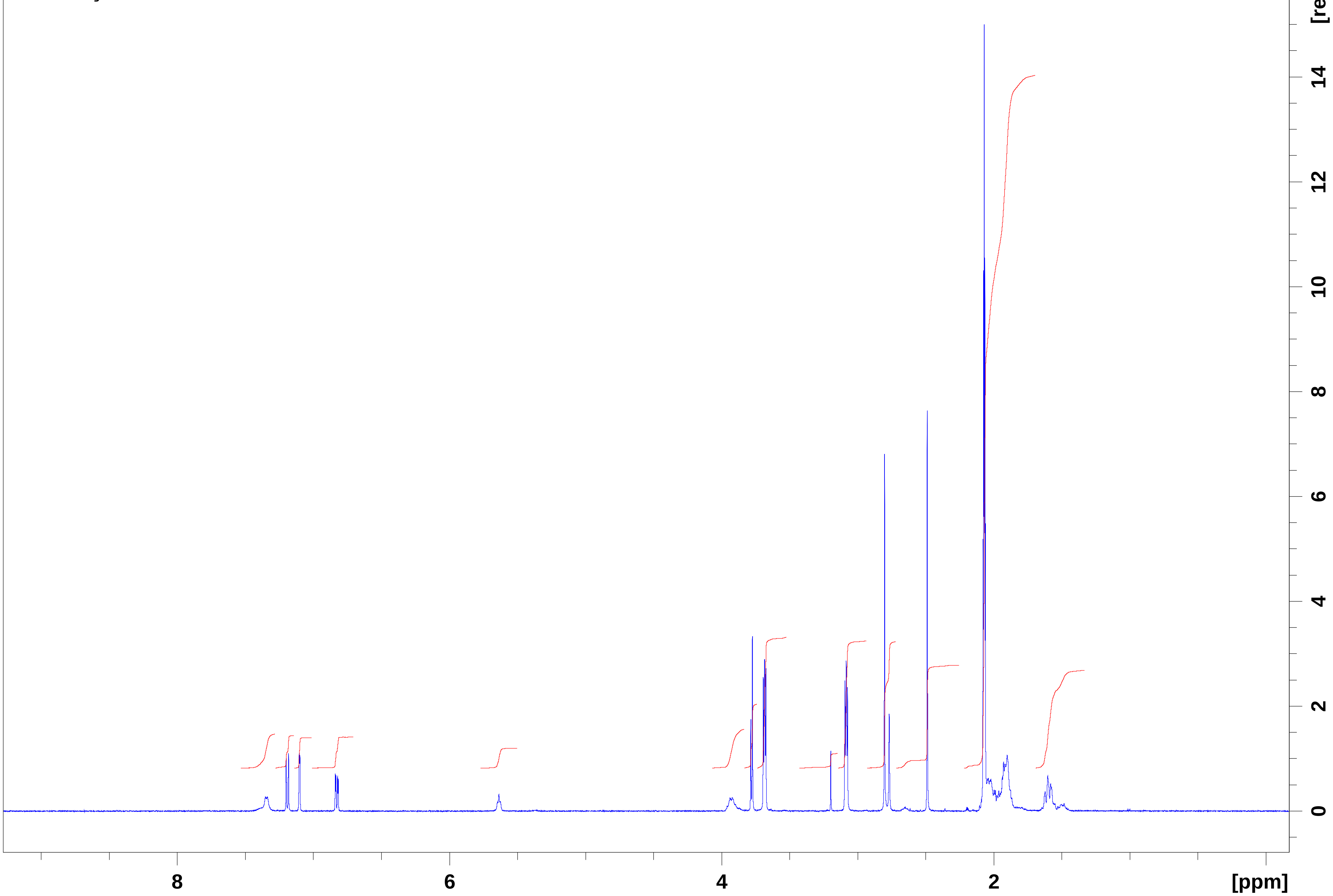

Compound 11 DMSO-d6

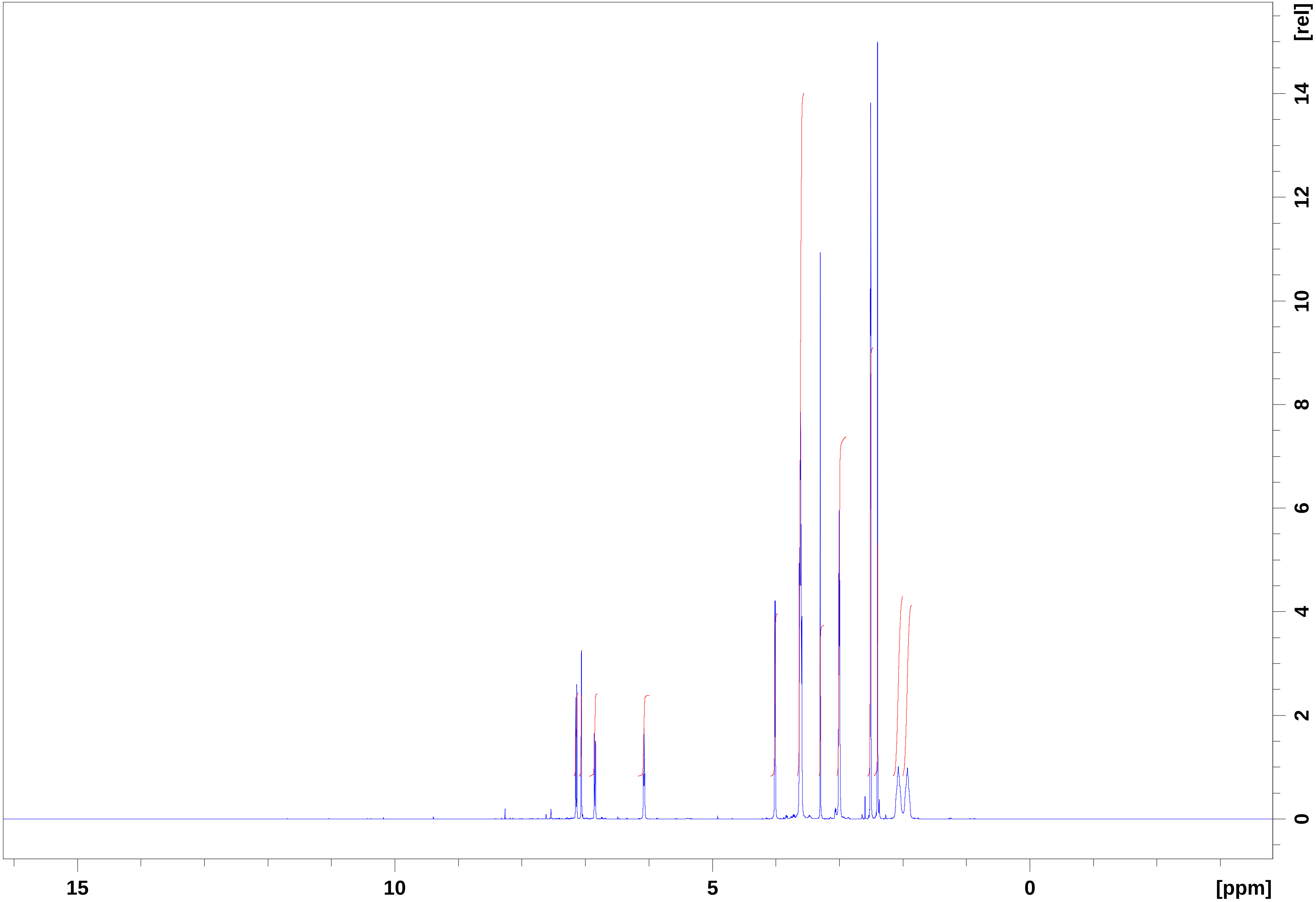

Compound 12

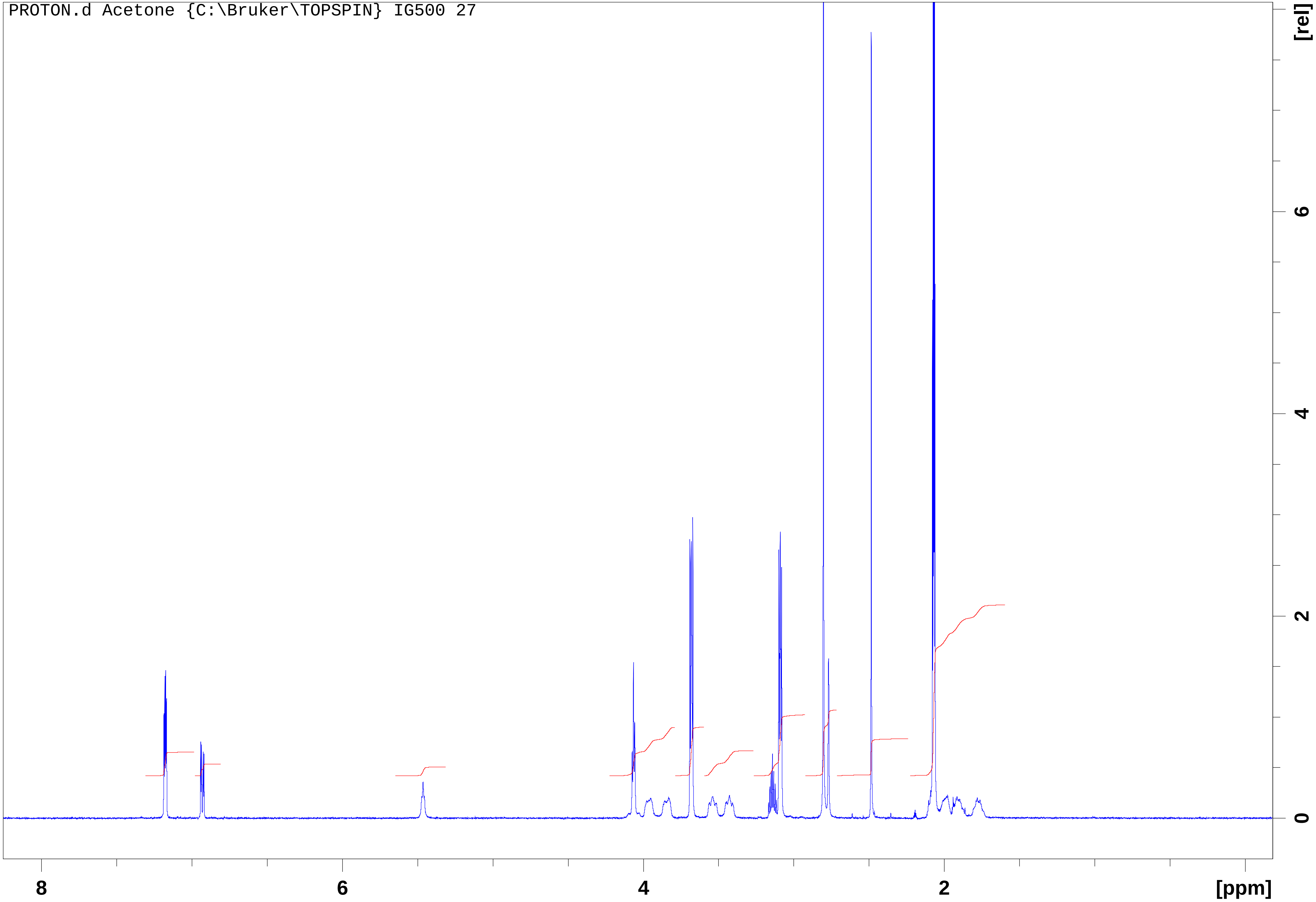

Compound 13

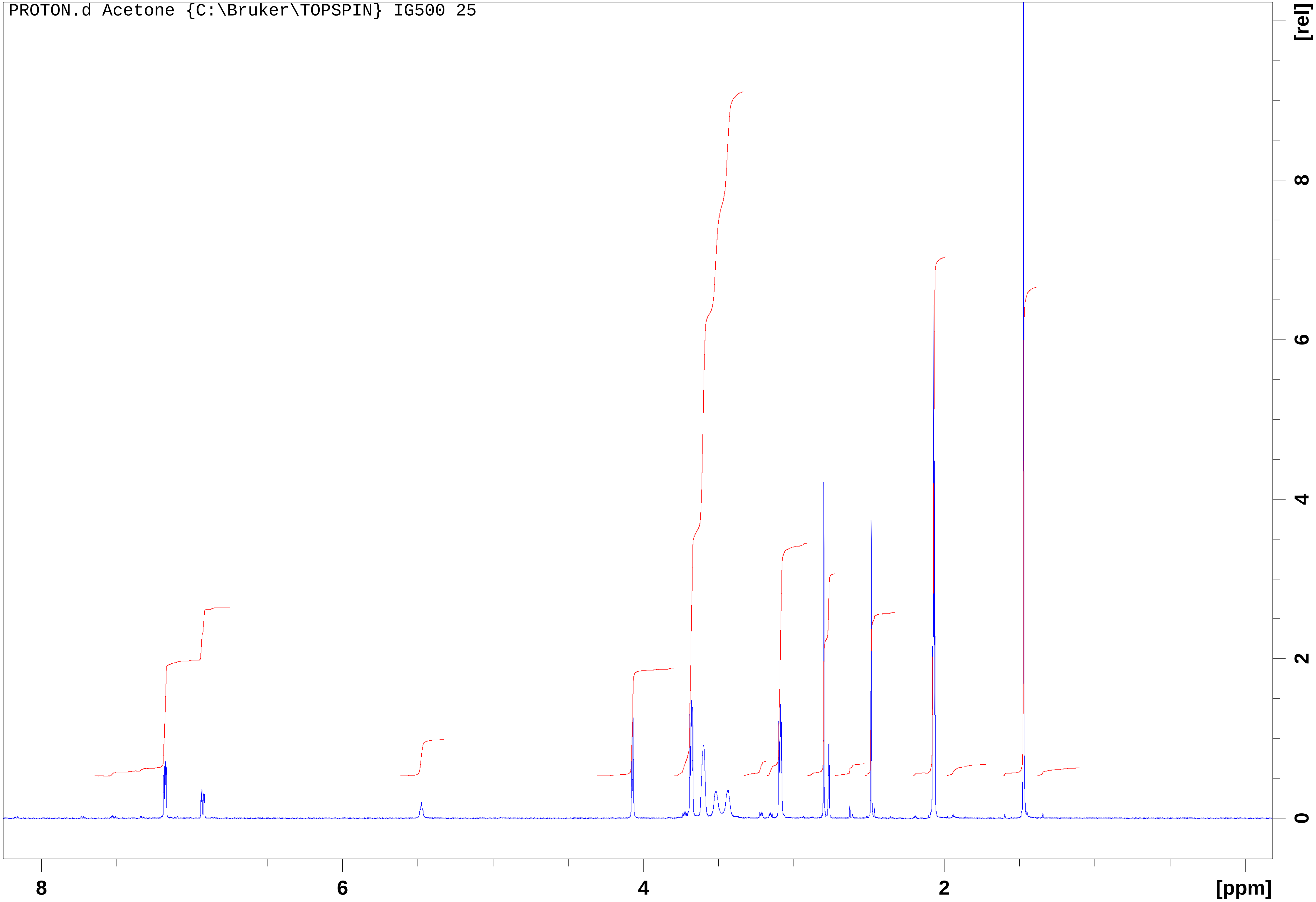
PROTON.d Acetone {C:\Bruker\TOPSPIN} IG500 25
[rel]
8
6
4
2
0
8
6
4
2
[ppm]

Compound 14

PROTON.d Acetone {C:\Bruker\TOPSPIN} IG500 24

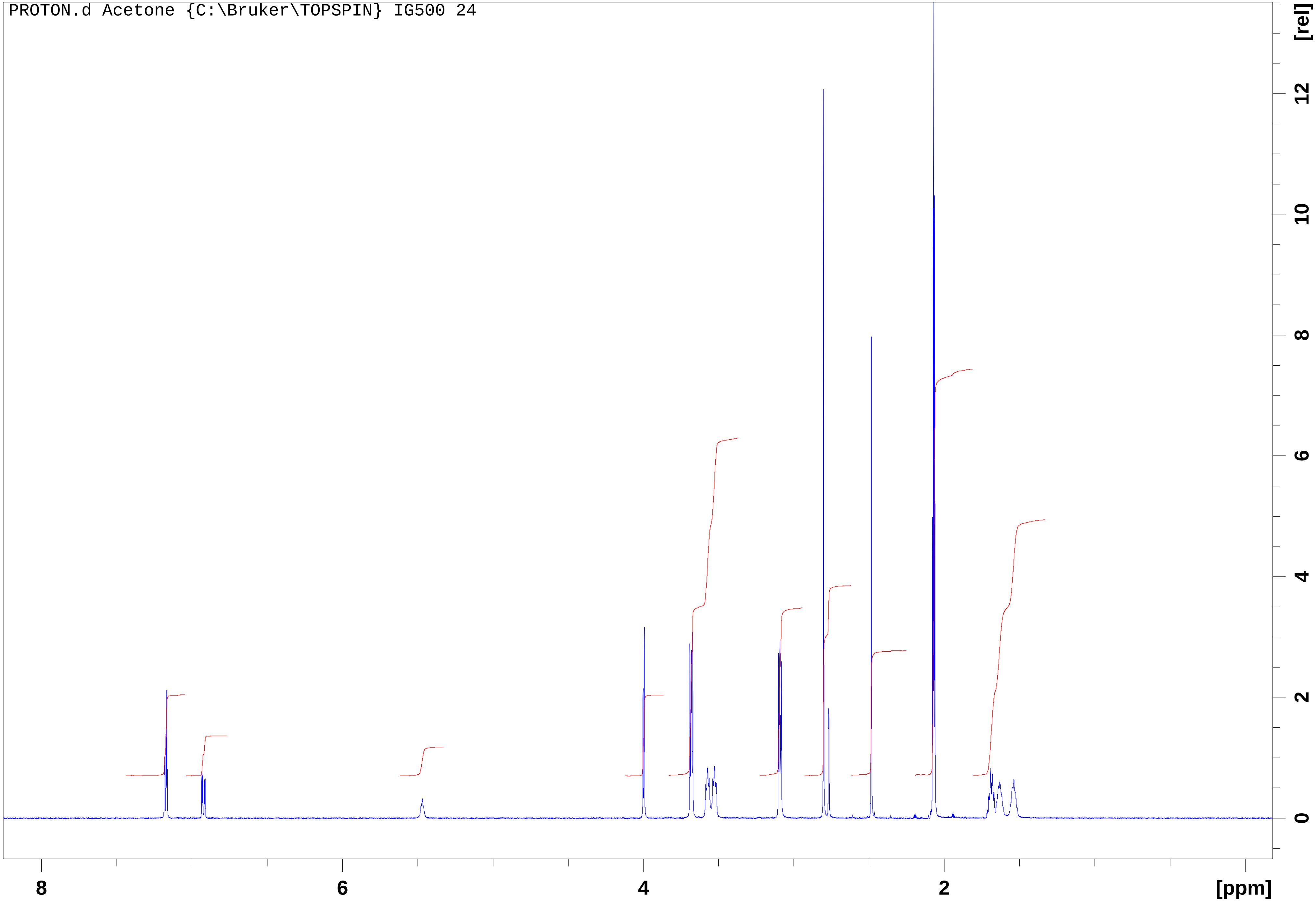

Compound 15 PROTON.d DMSO {C:\Bruker\TOPSPIN} IG500 23

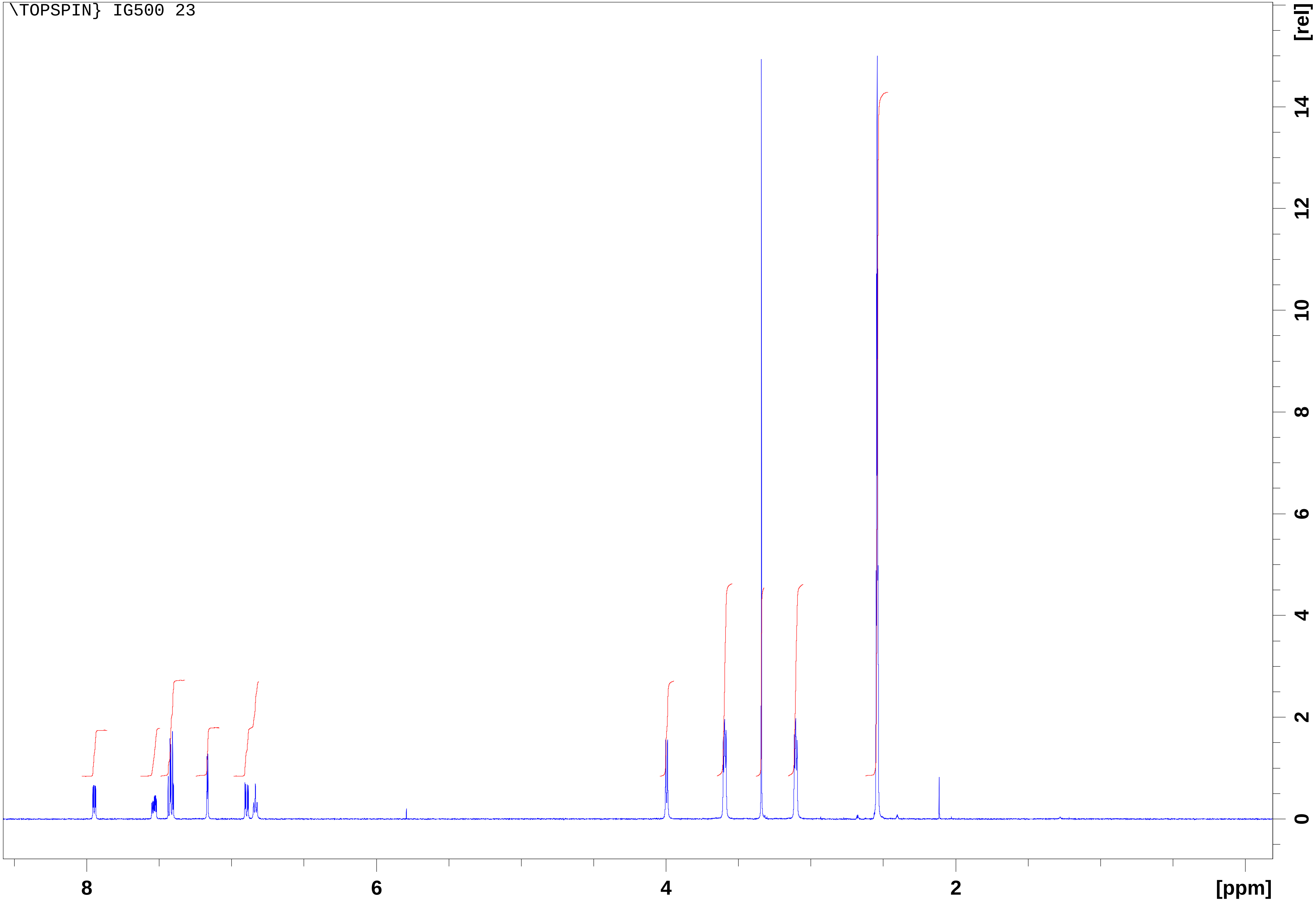

Compound 16

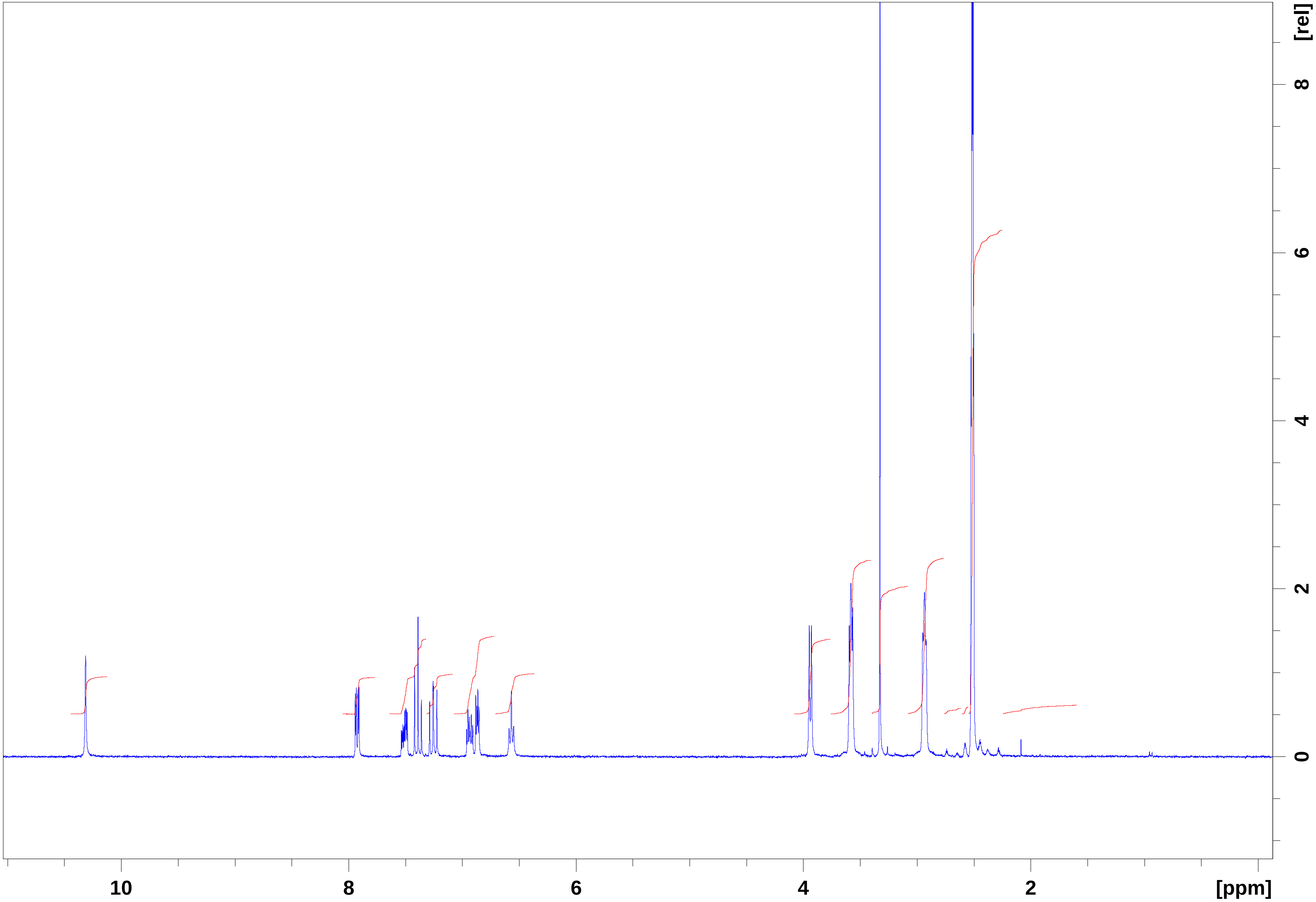

Compound 17 PROTON.d DMSO {C:\Bruker \TOPSPIN} IG500 20

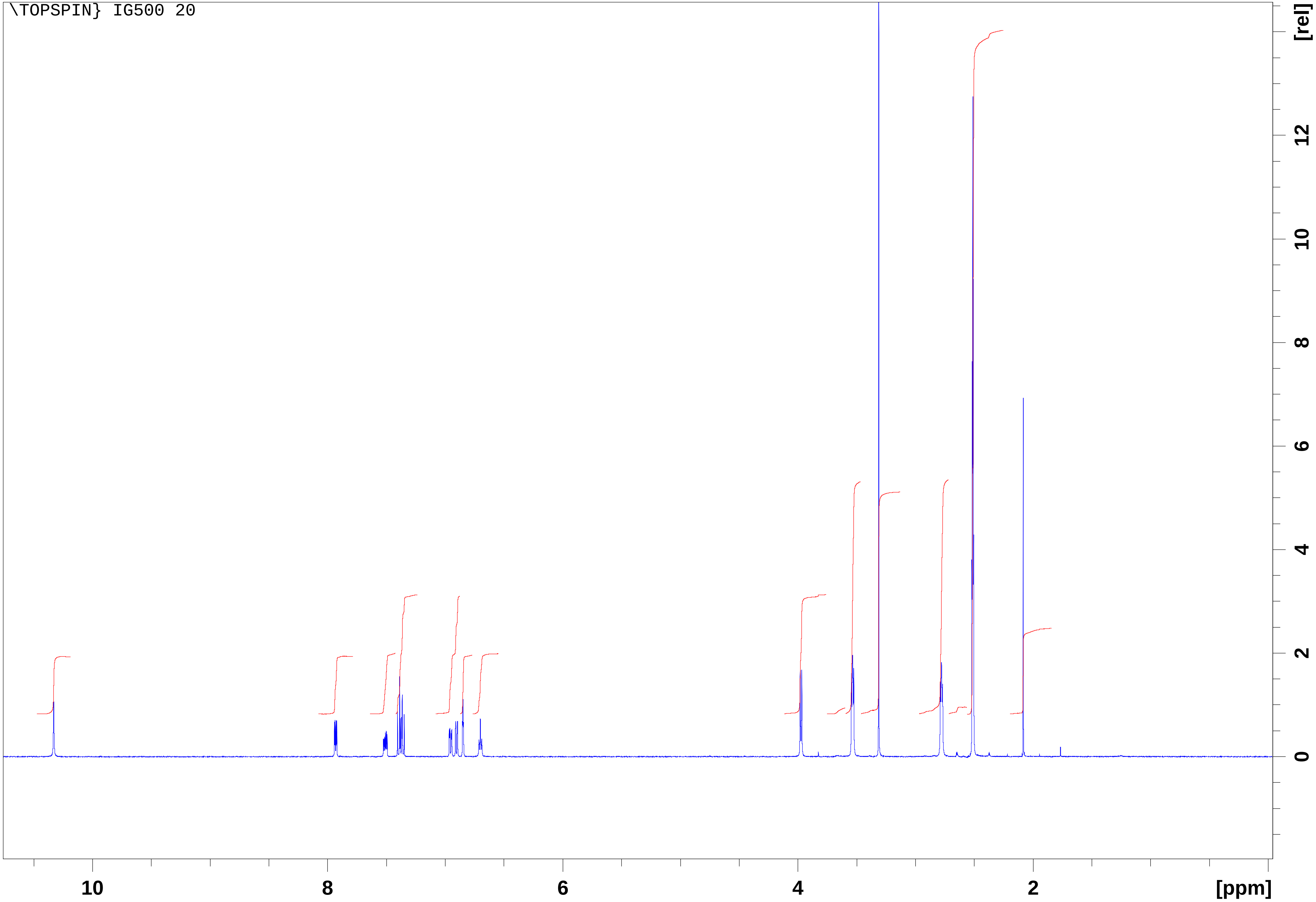

Compound 18 PROTON.d CDCl3 {C:\Bruker
\TOPSPIN} IG500 53

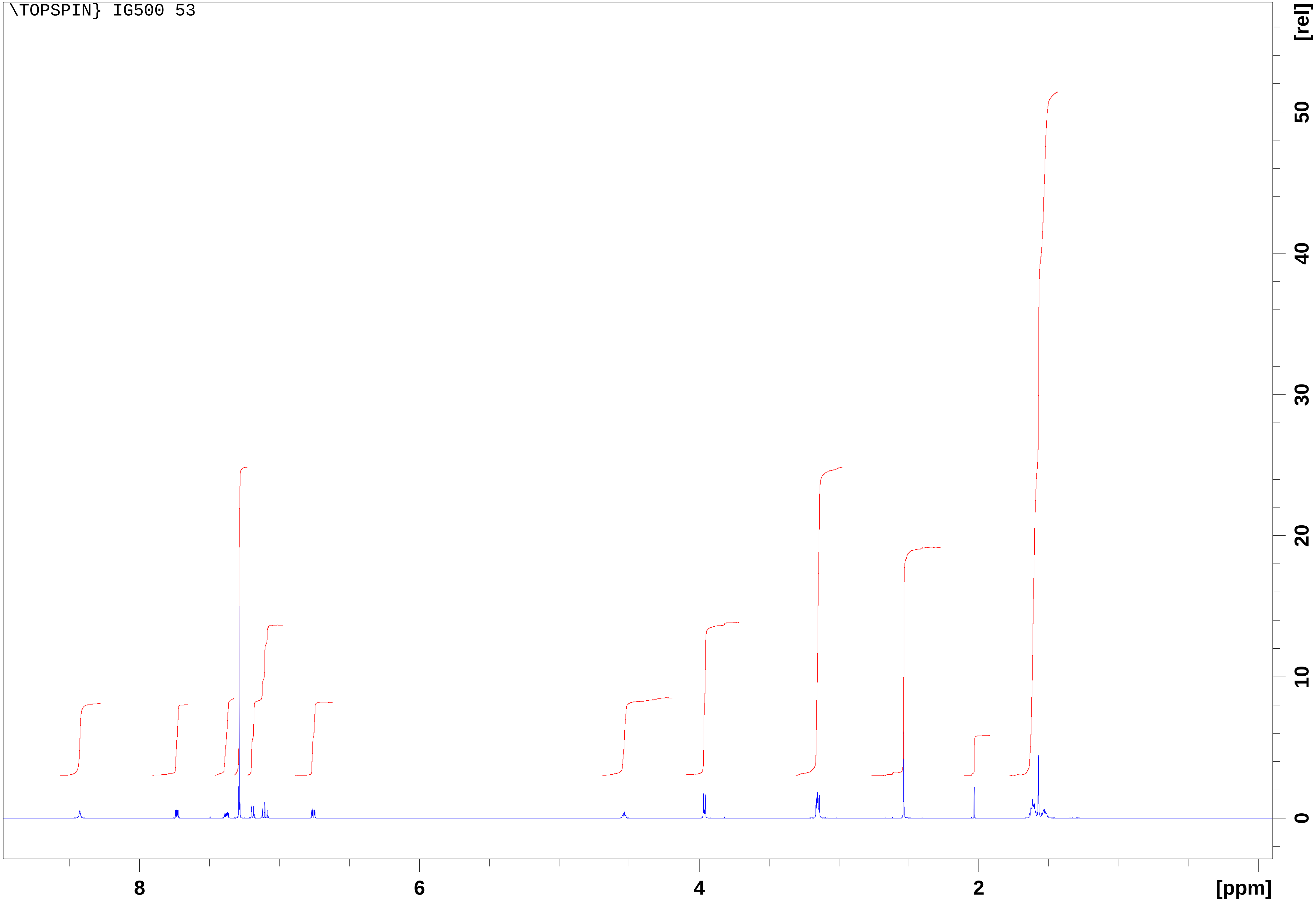

Compound 19 PROTON.d CDCl3 {C:\Bruker \TOPSPIN} IG500 56

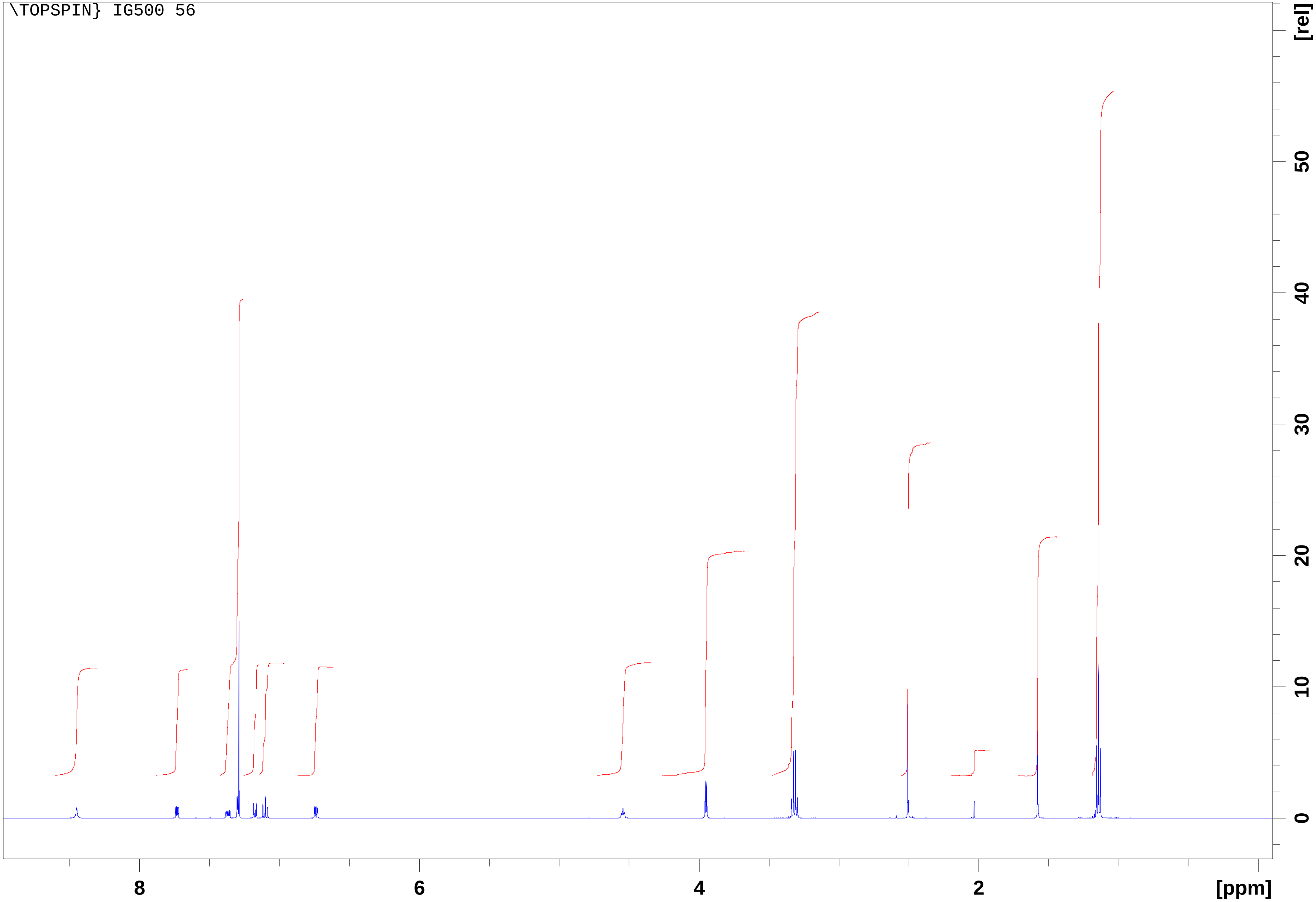

Compound 20

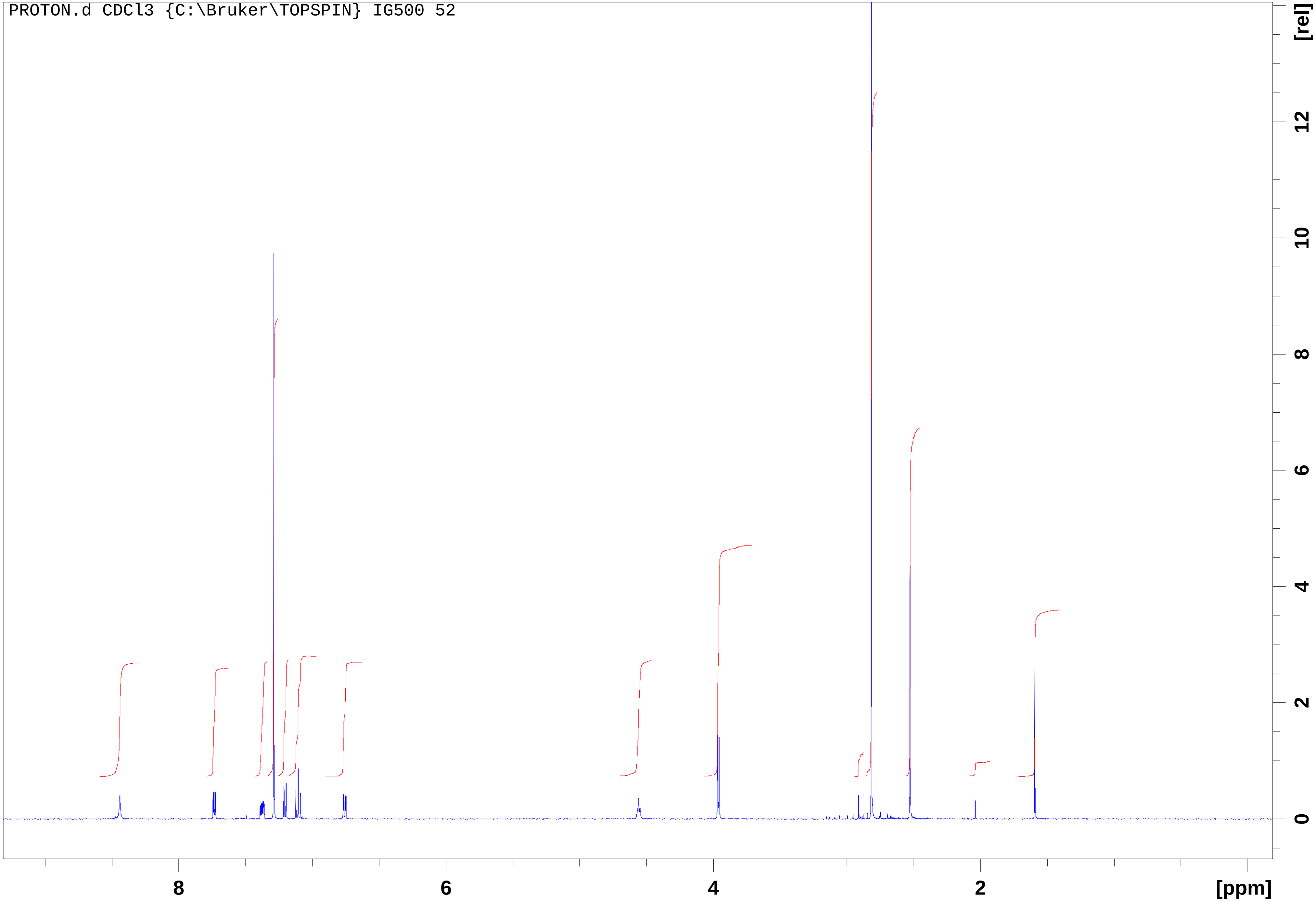

PROTON.d CDCl3 {C:\Bruker\TOPSPIN} IG500 52
[rel]
12
10
8
6
4
2
0
8
6
4
2
[ppm]

Compound 21

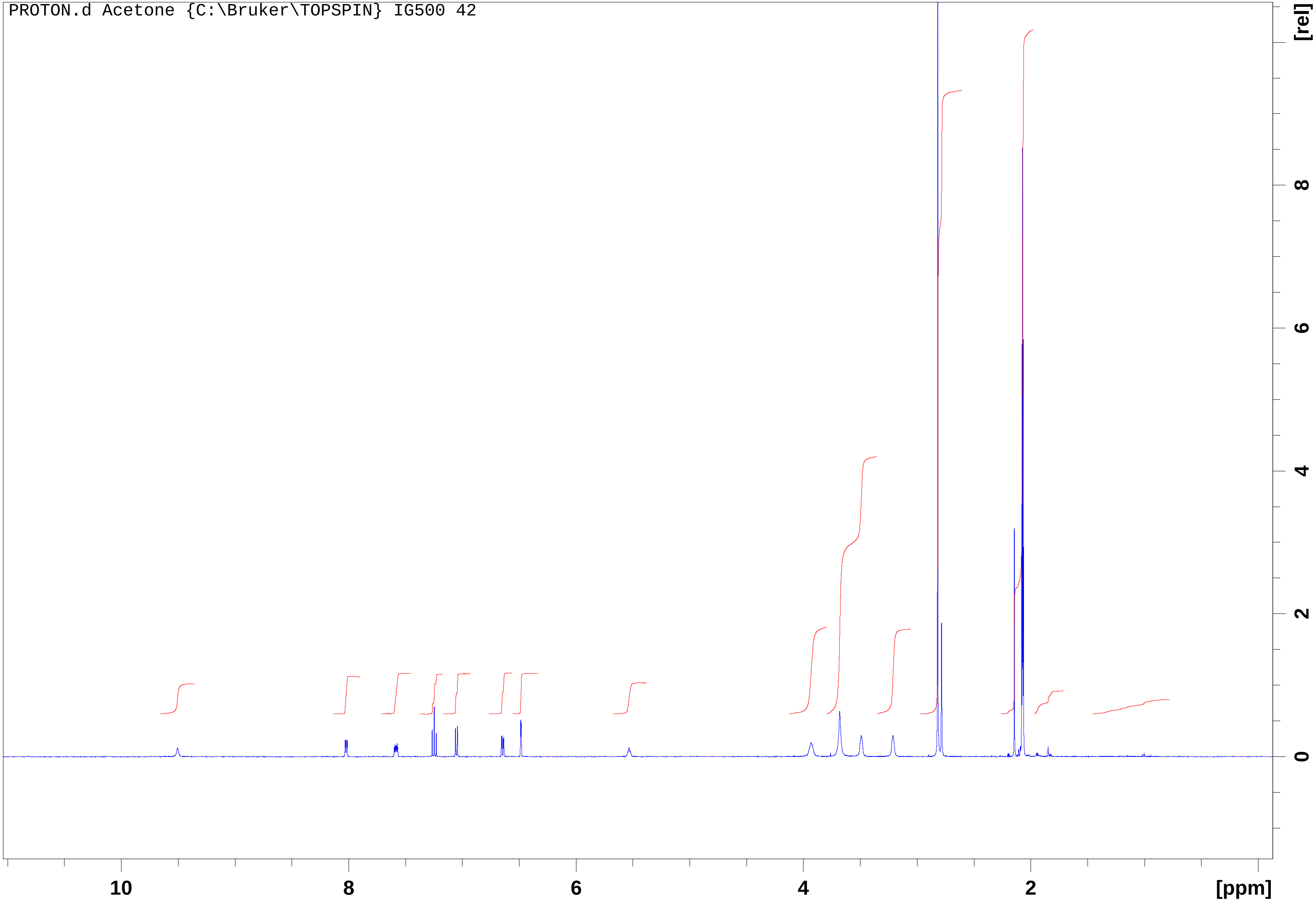
PROTON.d Acetone {C:\Bruker\TOPSPIN} IG500 42
[rel]
8
6
4
2
0
10
8
6
4
2
[ppm]

Compound 22 PROTON.d CDCl3 {C:\Bruker \TOPSPIN} IG500 23

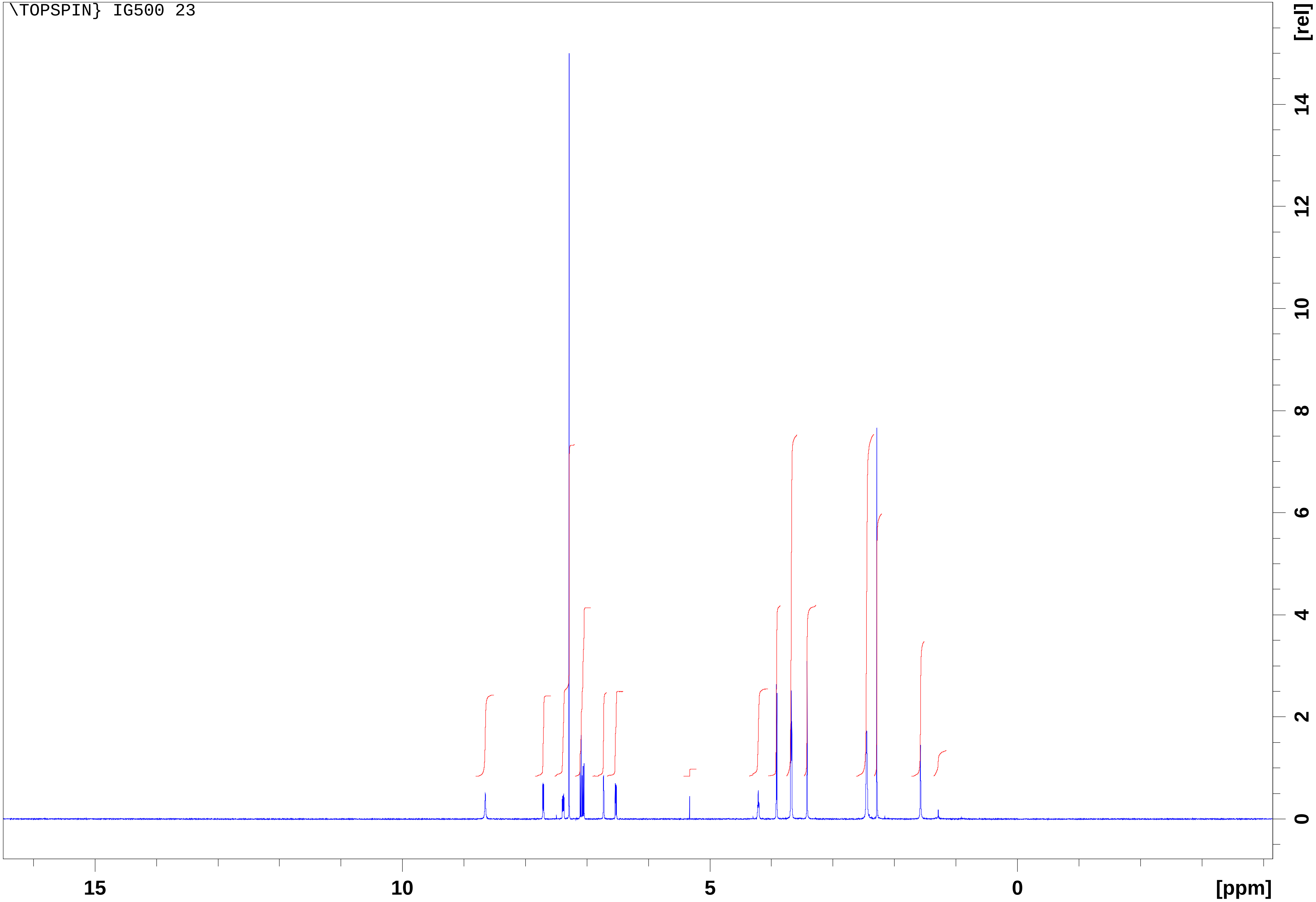

Compound 23

PROTON.d DMSO {C:\Bruker\TOPSPIN} IG500 18

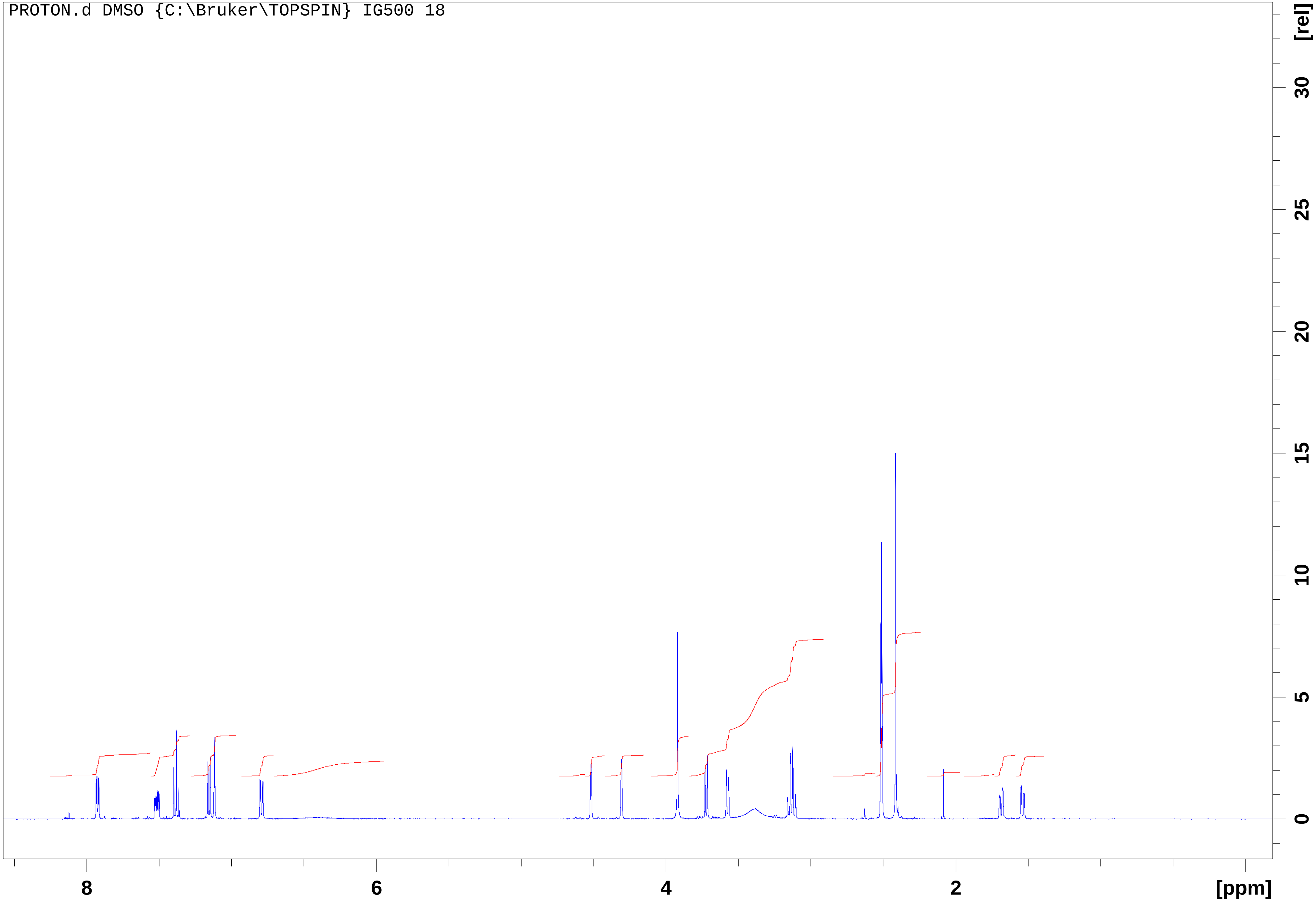

Compound 24

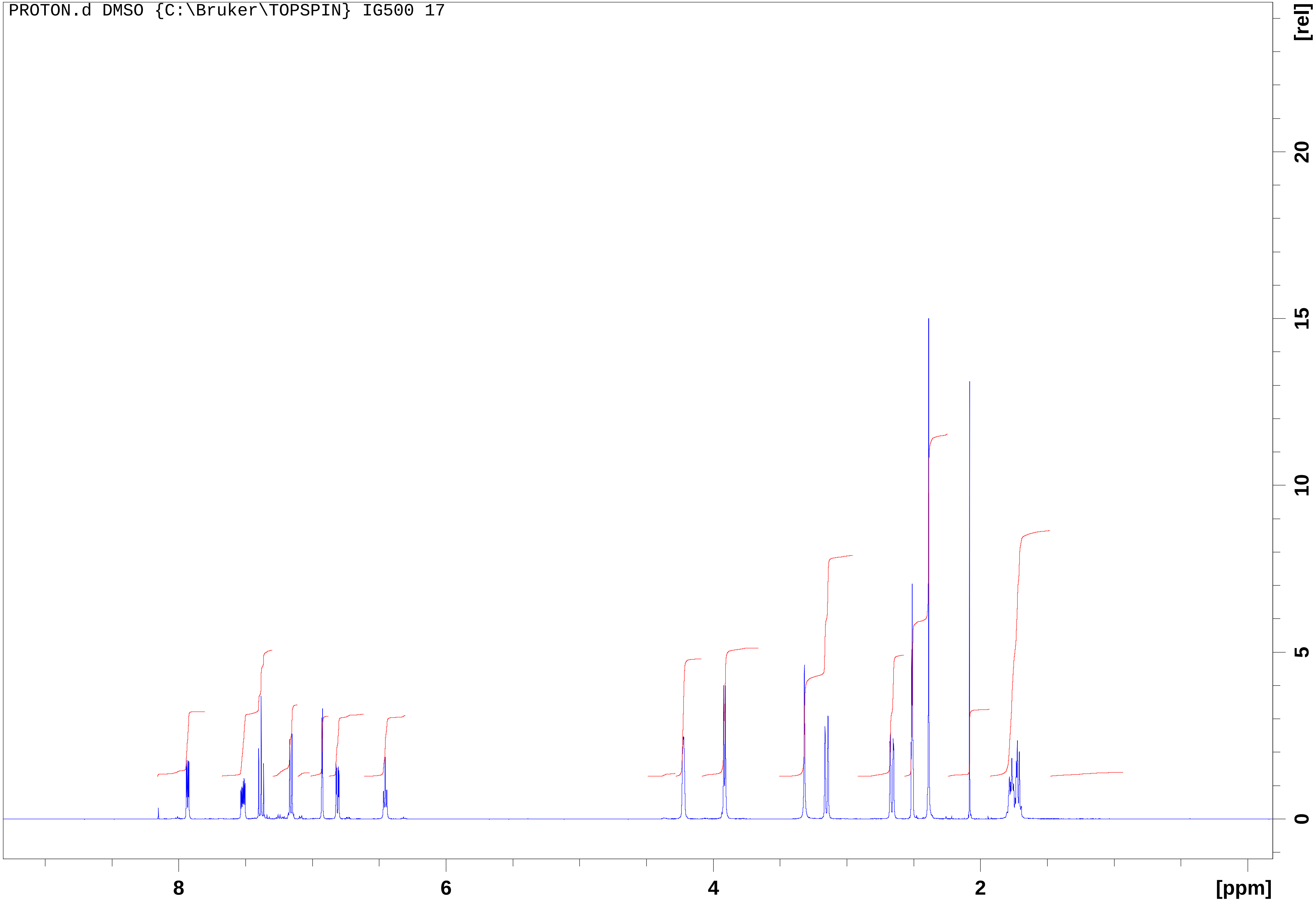
PROTON.d DMSO {C:\Bruker\TOPSPIN} IG500 17
[rel]
20
15
10
5
0
8
6
4
2
[ppm]

Compound 25

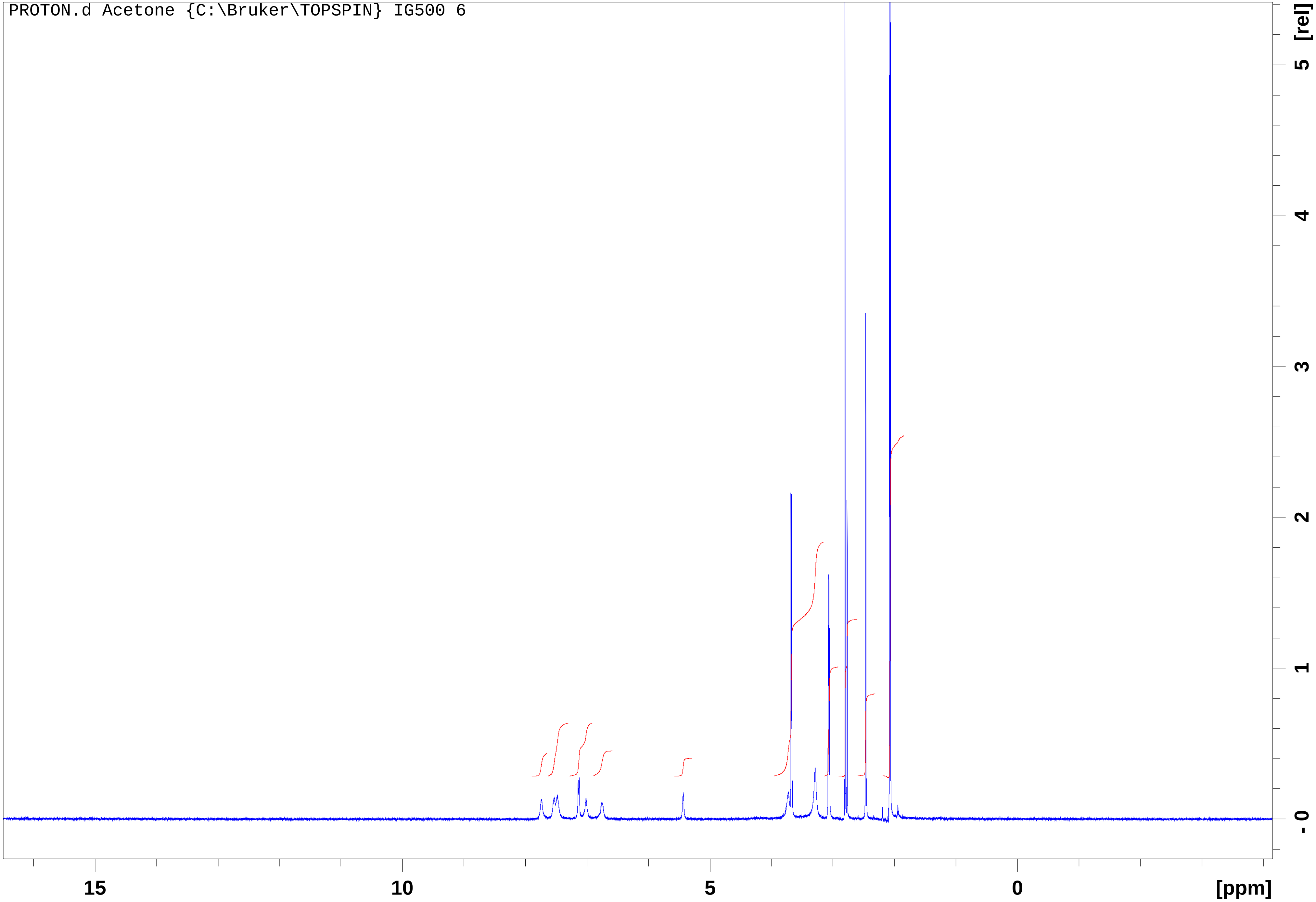

PROTON.d Acetone {C:\Bruker\TOPSPIN} IG500 6
[rel]
5
4
3
2
1
0
15
10
5
0
[ppm]

Compound 26 PROTON.d CDCl3 {C:\Bruker
\TOPSPIN} IG500 14

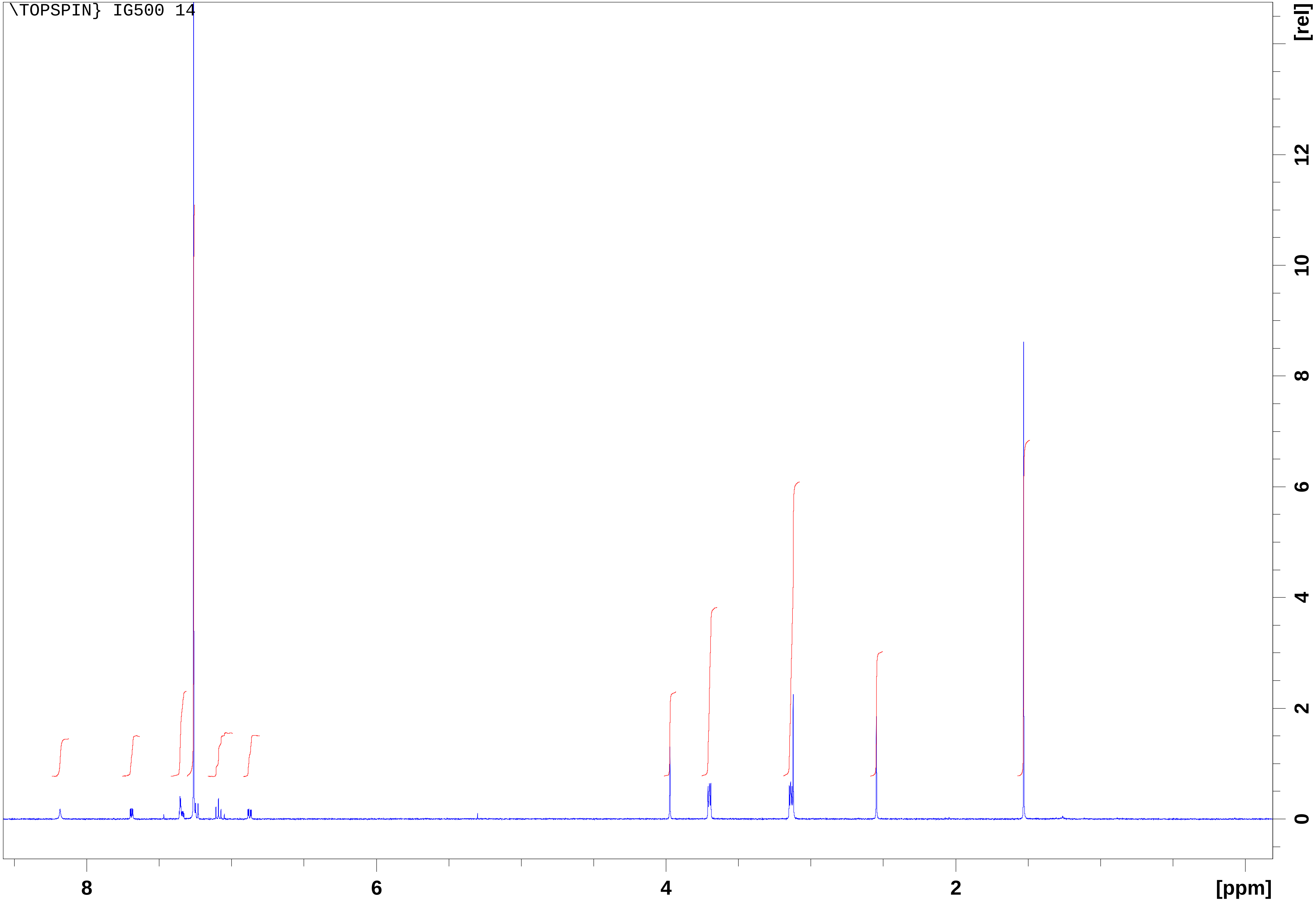

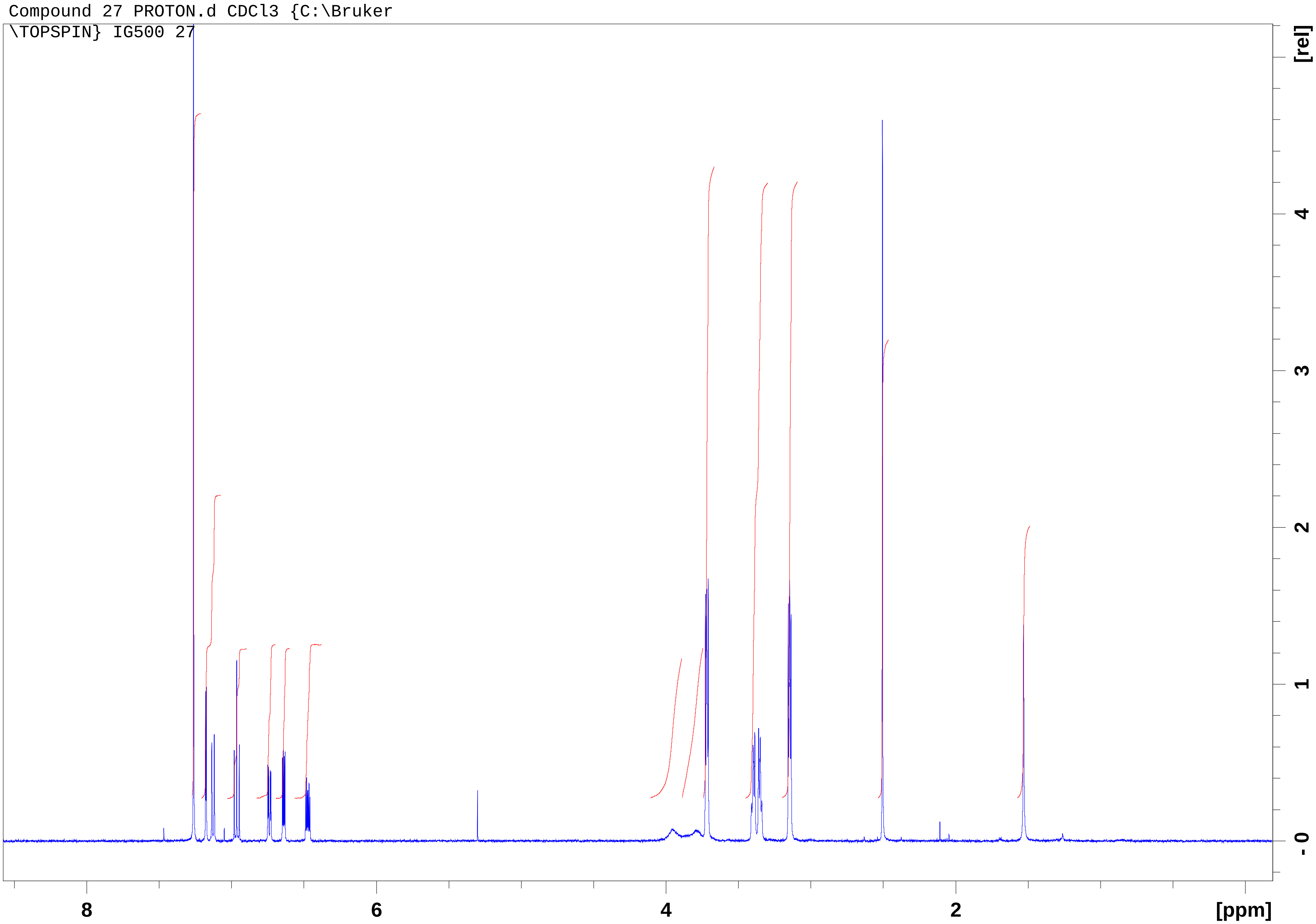
Compound 27 PROTON.d CDCl3 {C:\Bruker
\TOPSPIN} IG500 27
[rel]
4
3
2
1
0
8
6
4
2
[ppm]

Compound 28

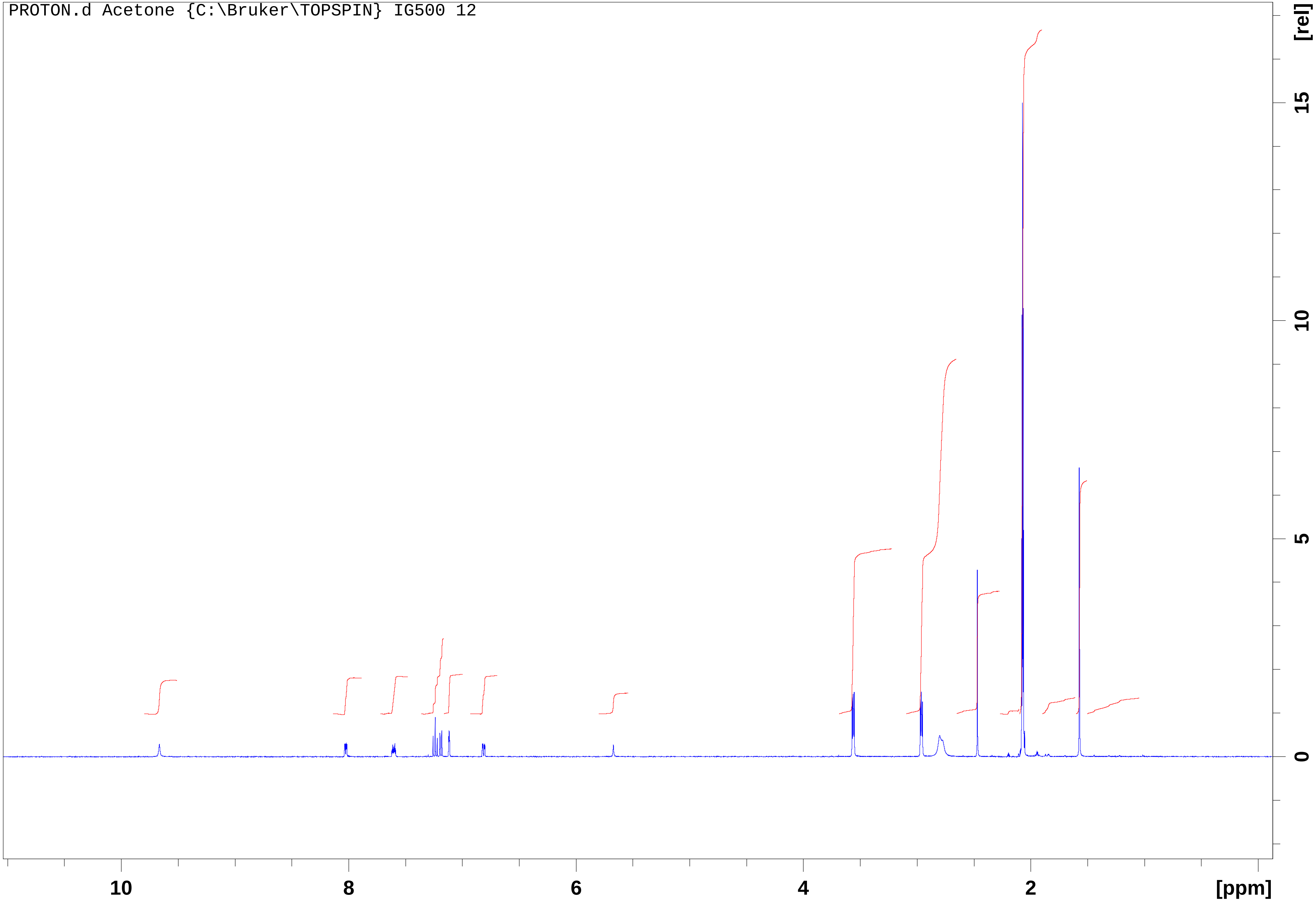

PROTON.d Acetone {C:\Bruker\TOPSPIN} IG500 12
[rel]
15
10
5
0
10
8
6
4
2
[ppm]

Compound 29

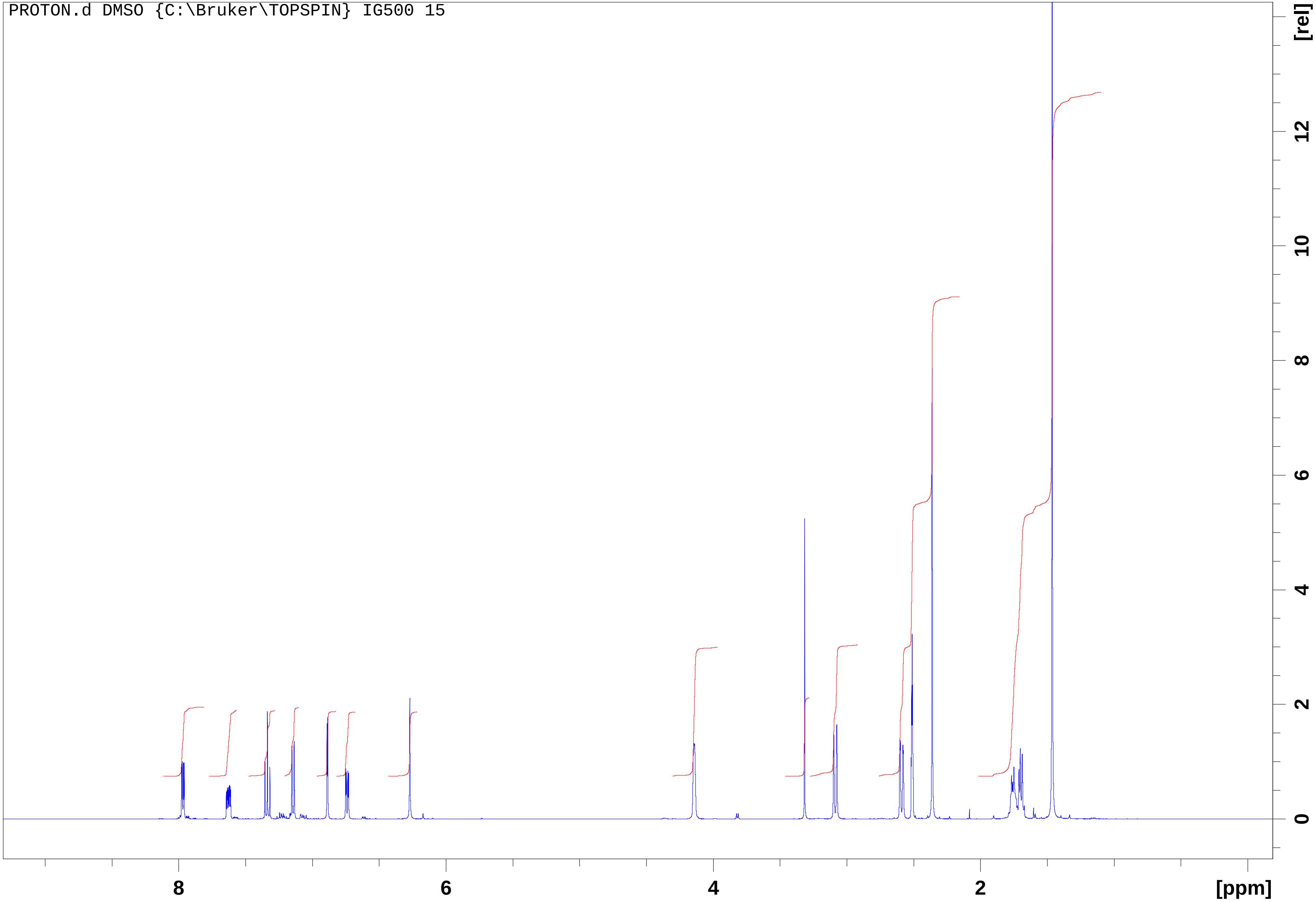

PROTON.d DMSO {C:\Bruker\TOPSPIN} IG500 15
[rel]
12
10
8
6
4
2
0
8
6
4
2
[ppm]

Compound 30 PROTON.d Acetone {C:\Bruker \TOPSPIN} IG500 8

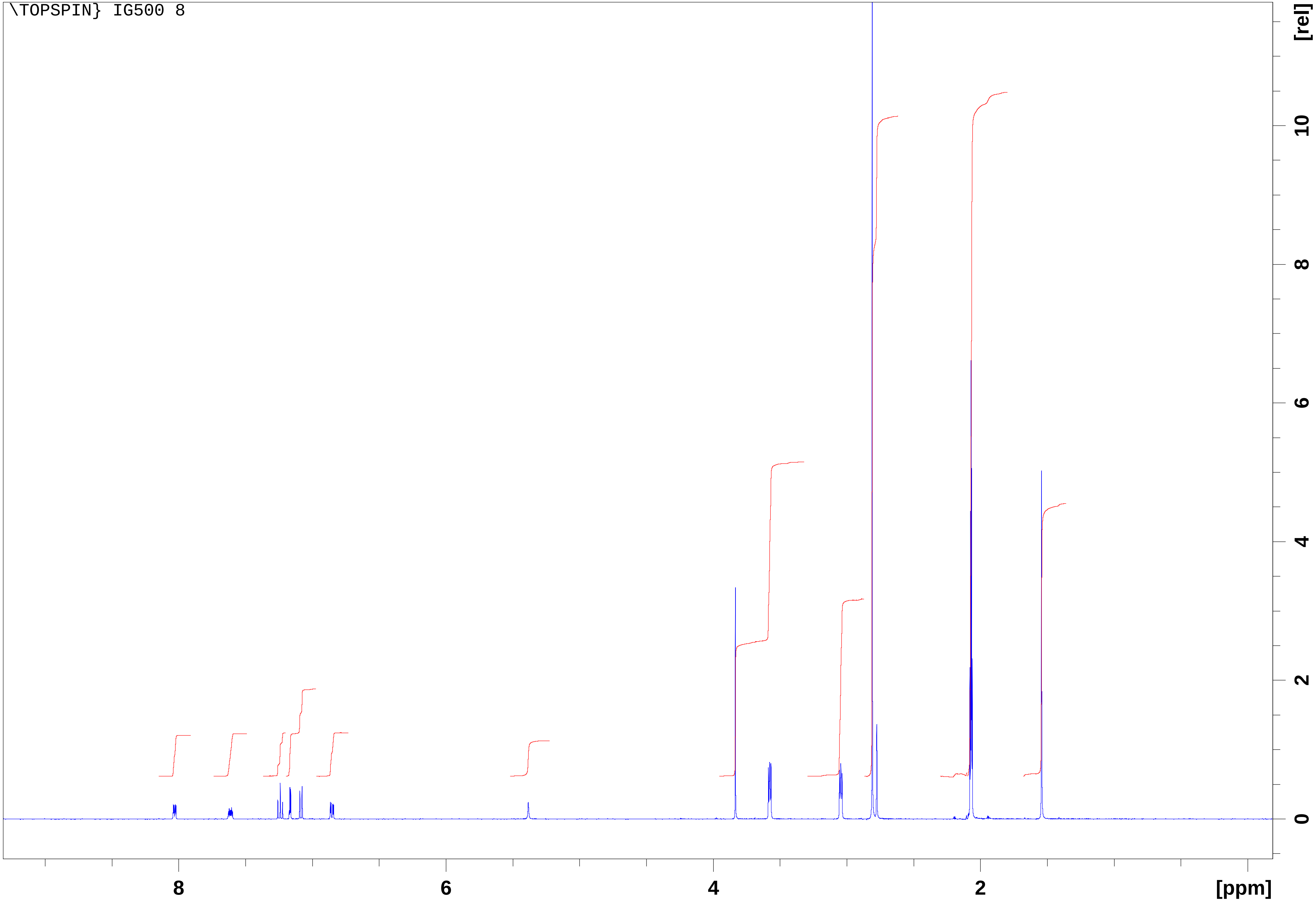

Compound 31

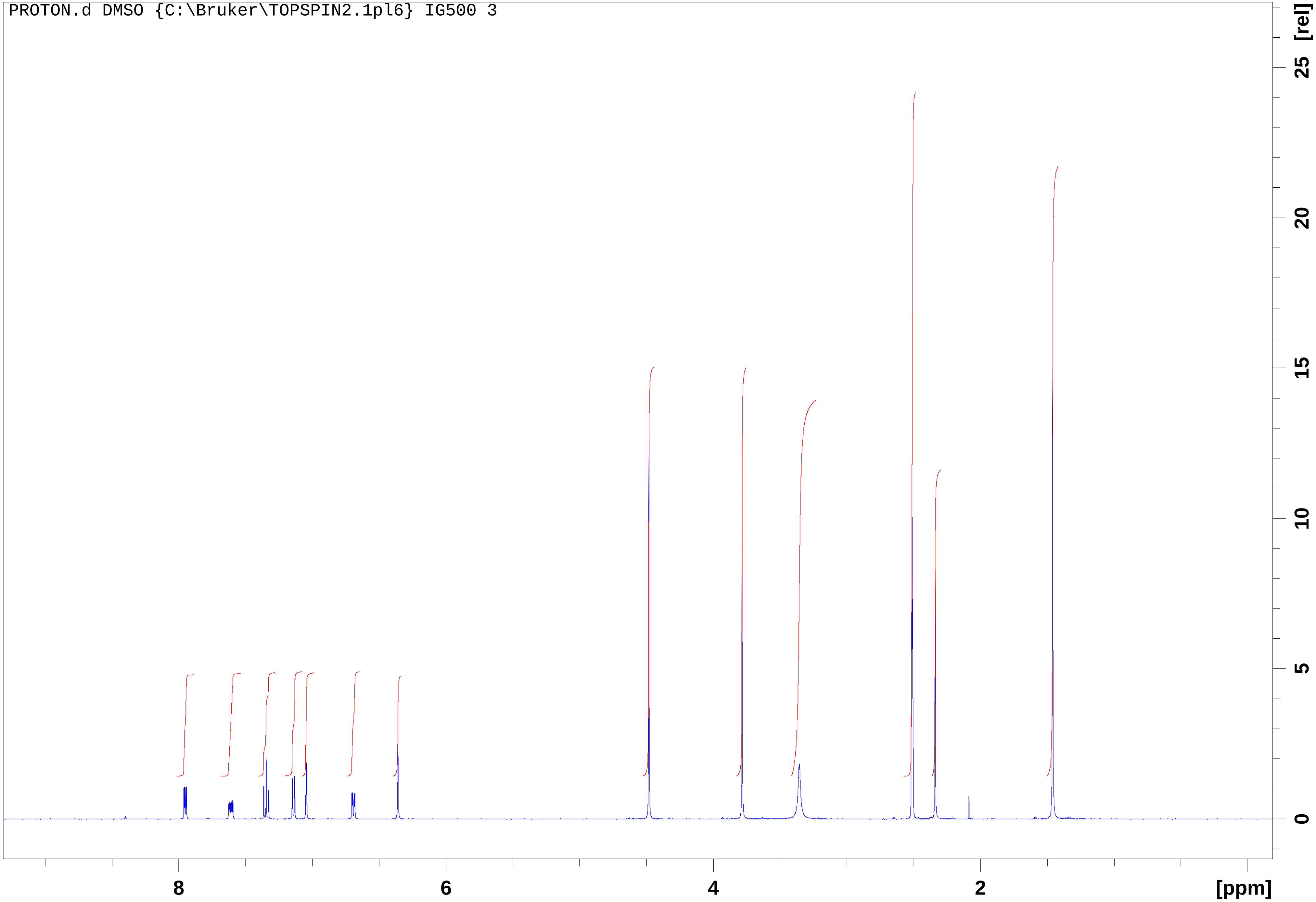
PROTON.d DMSO {C:\Bruker\TOPSPIN2.1pl6} IG500 3
[rel]
25
20
15
10
5
0
8
6
4
2
[ppm]

Compound 32

PROTON.d DMSO {C:\Bruker\TOPSPIN} IG500 16

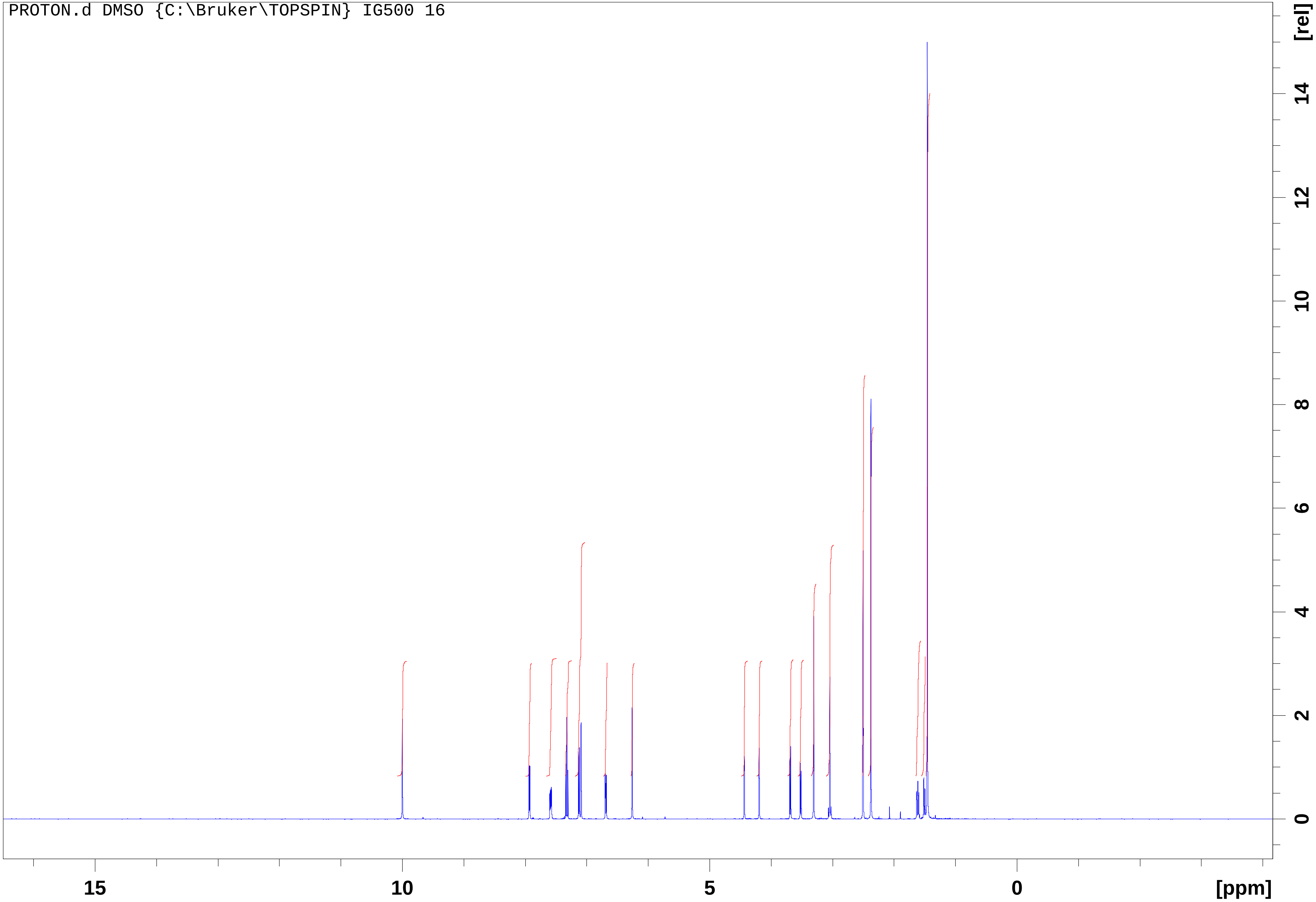

Compound 1

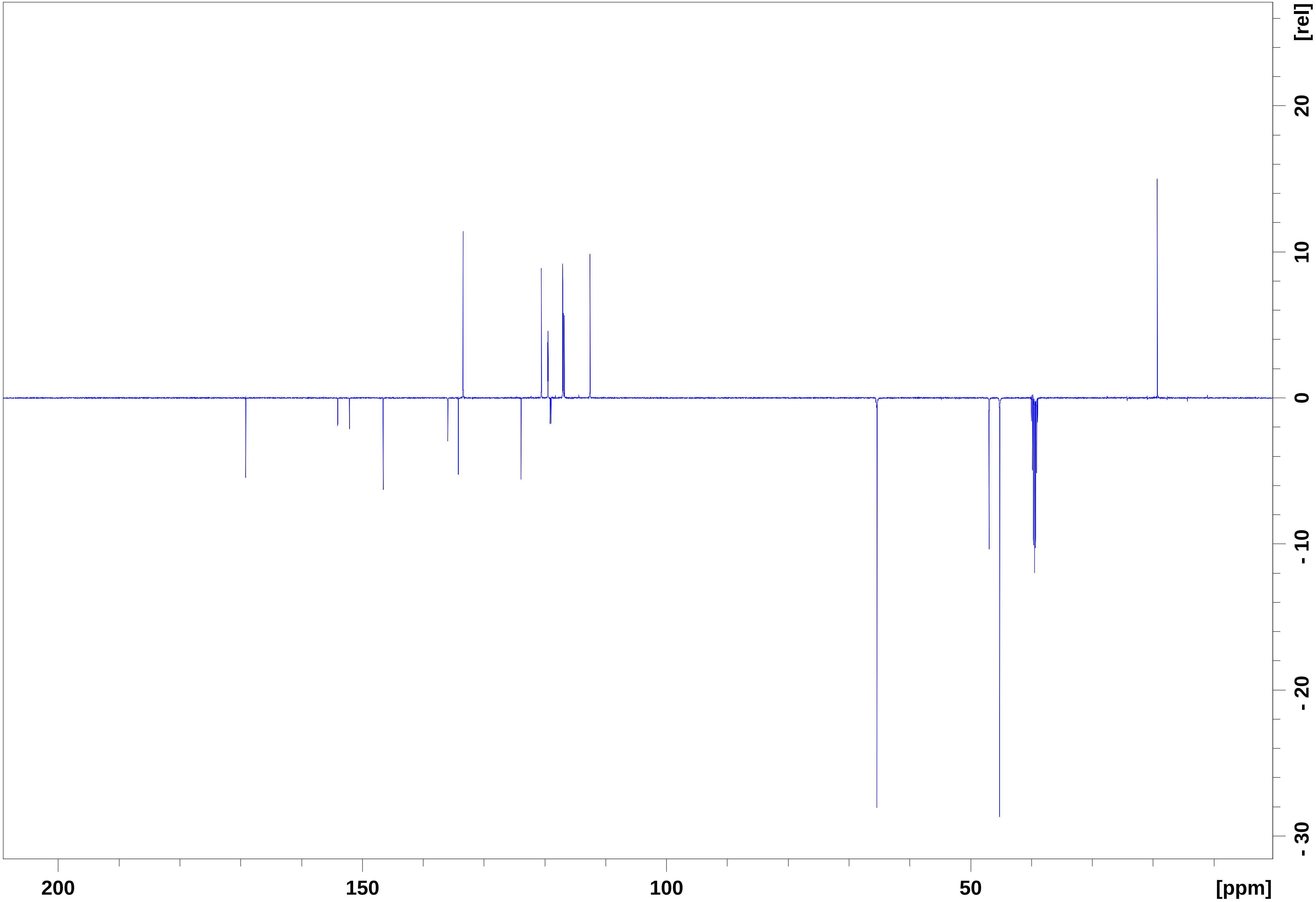

compound 2

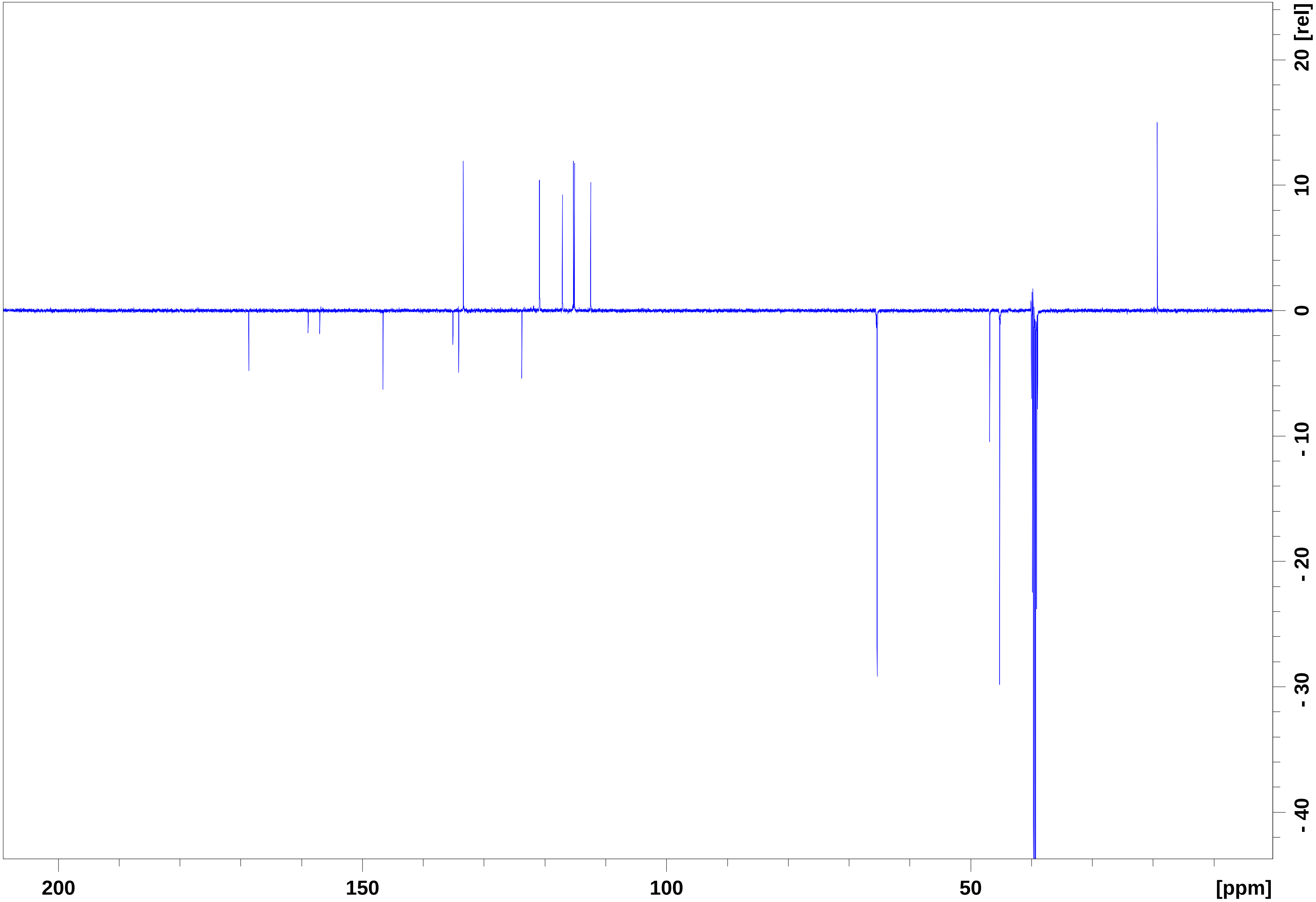

Compound 7

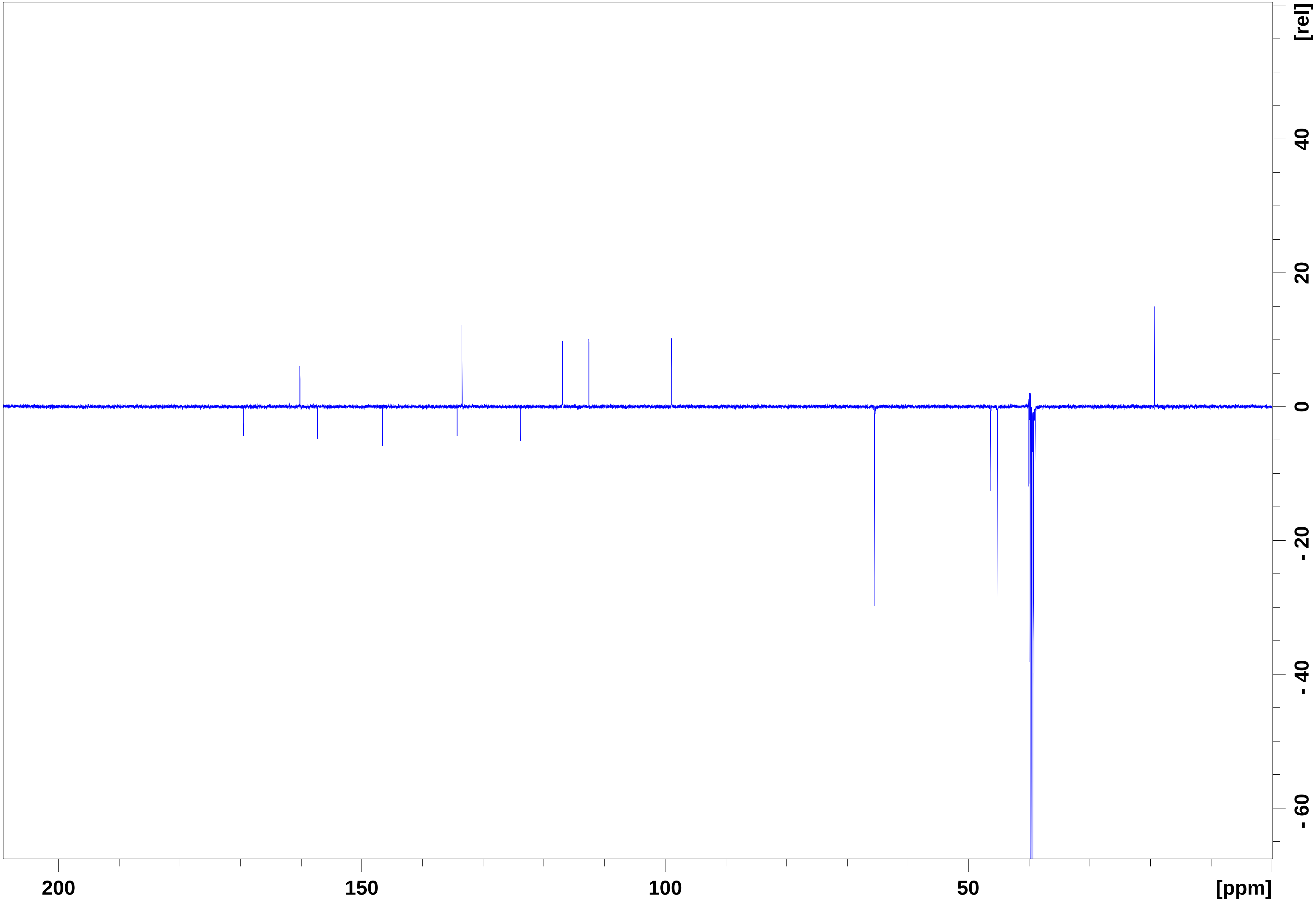

Compound 8

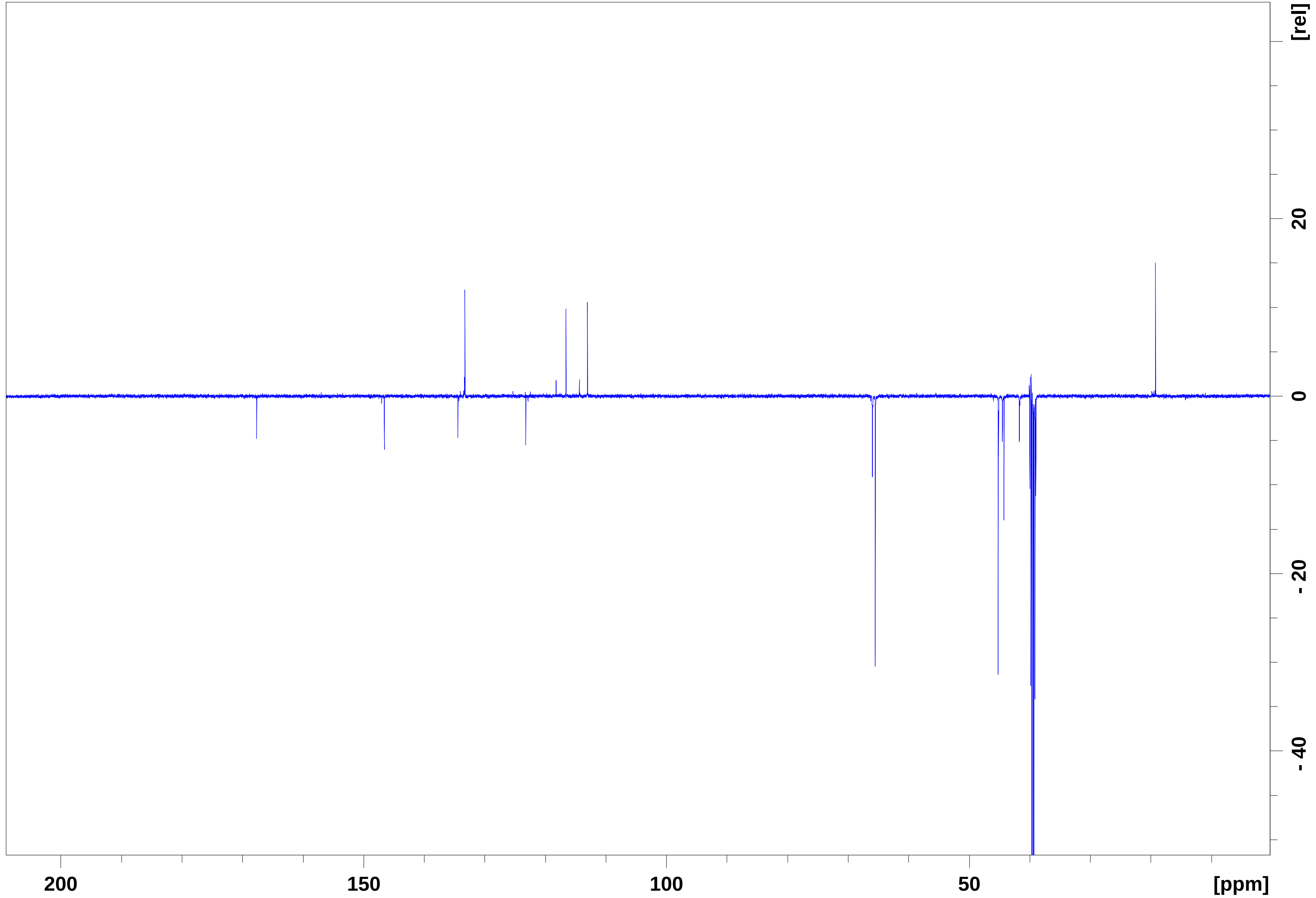

Compound 9

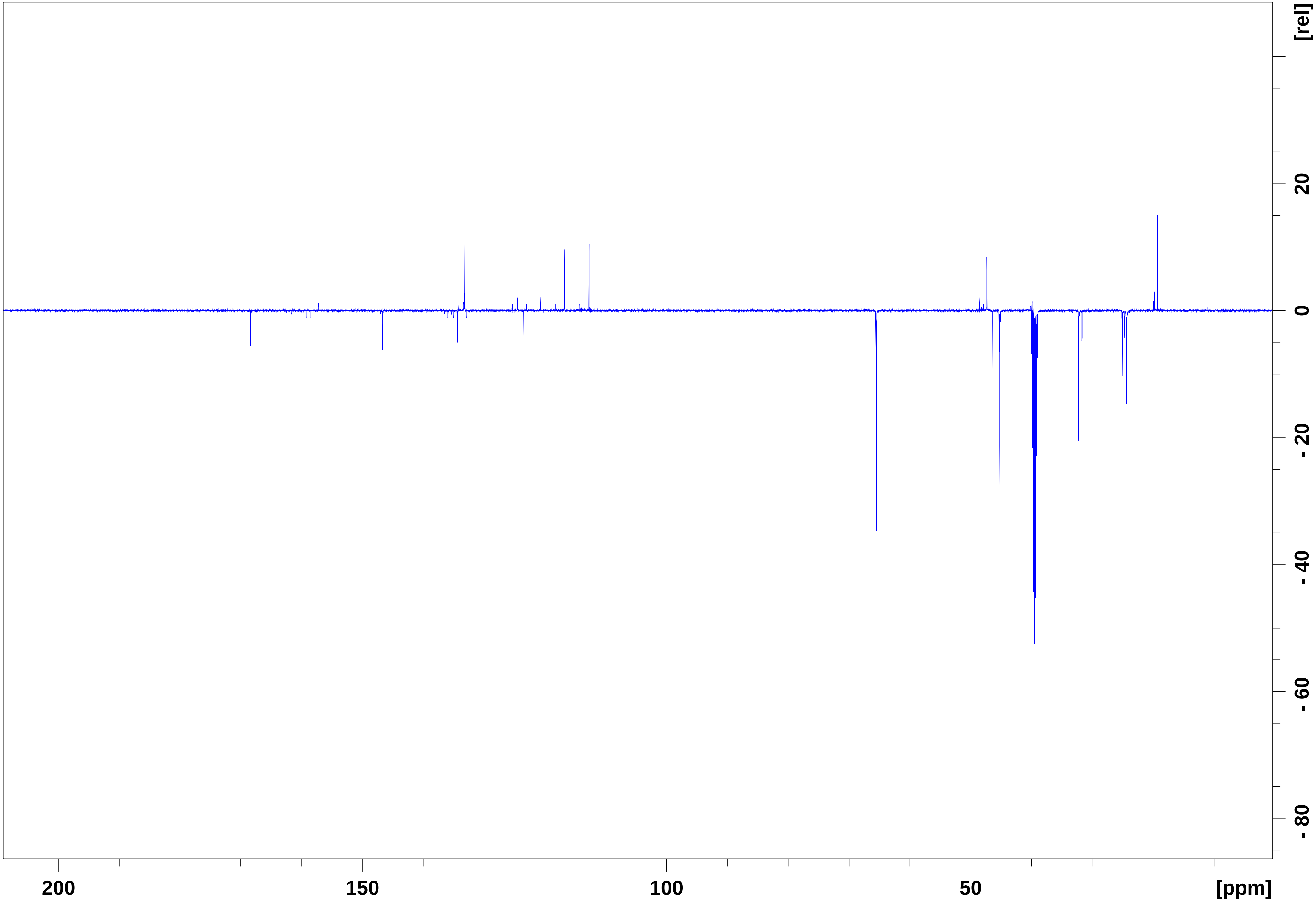

Compound 11

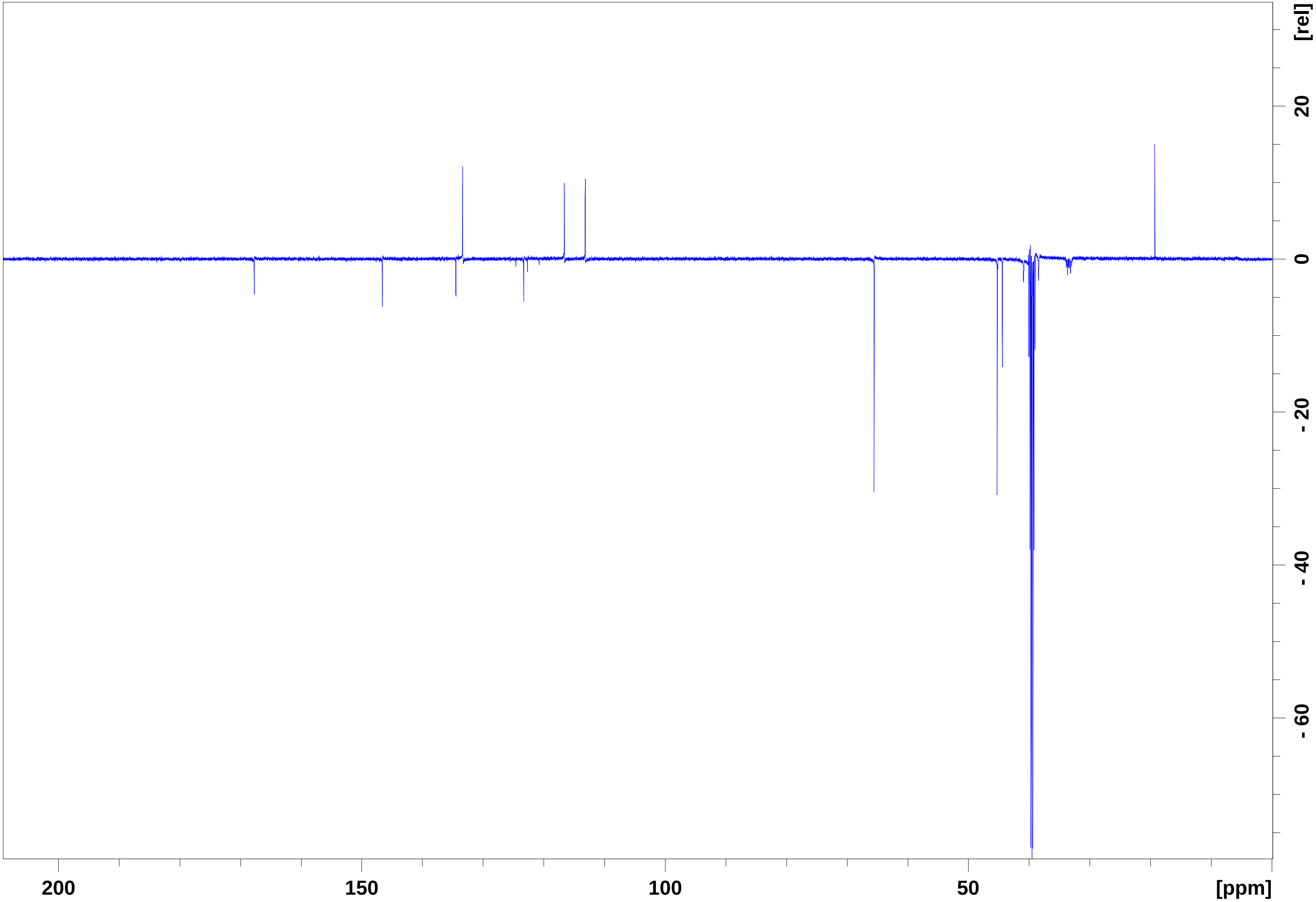

Compound 12

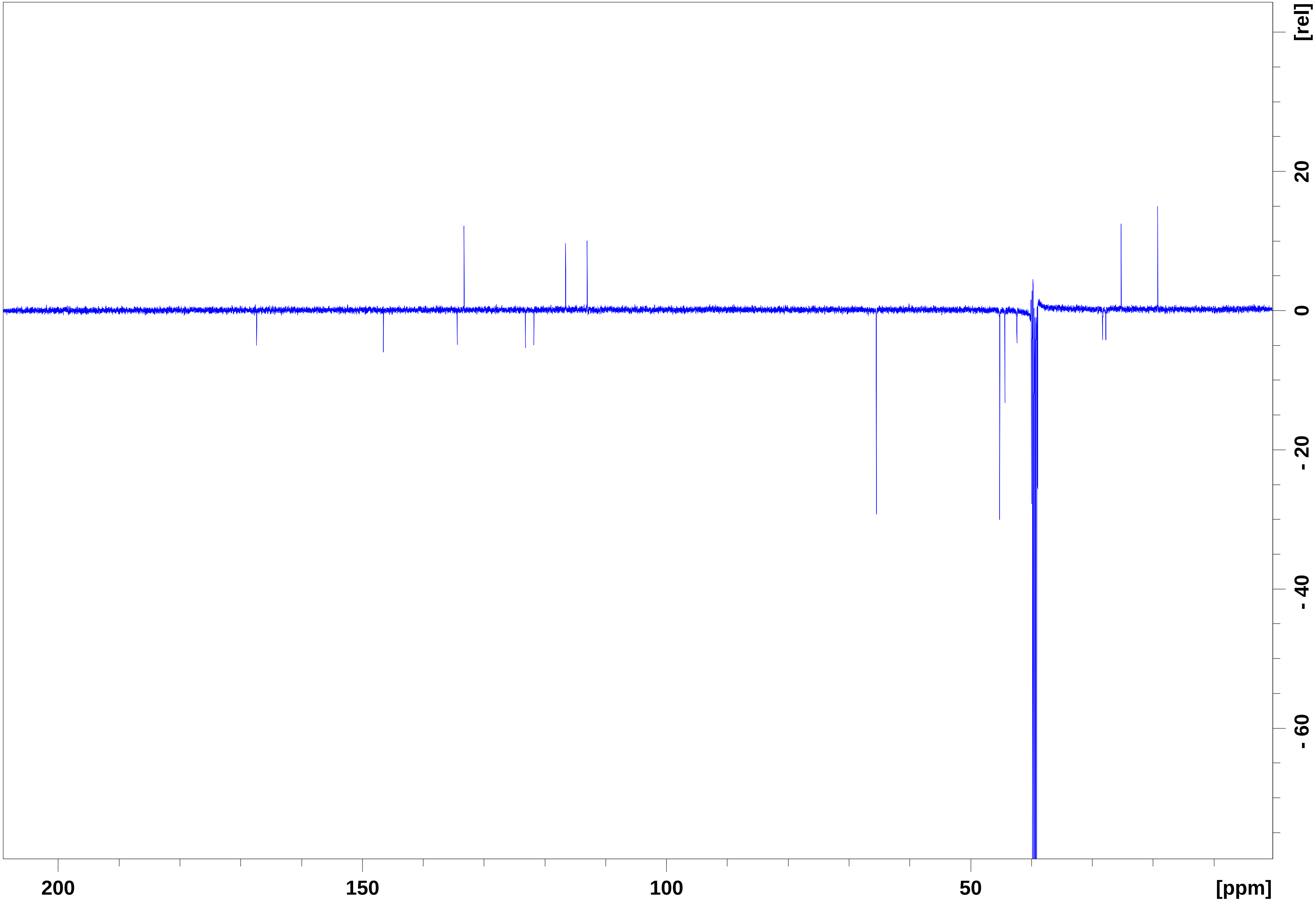

Compound 14

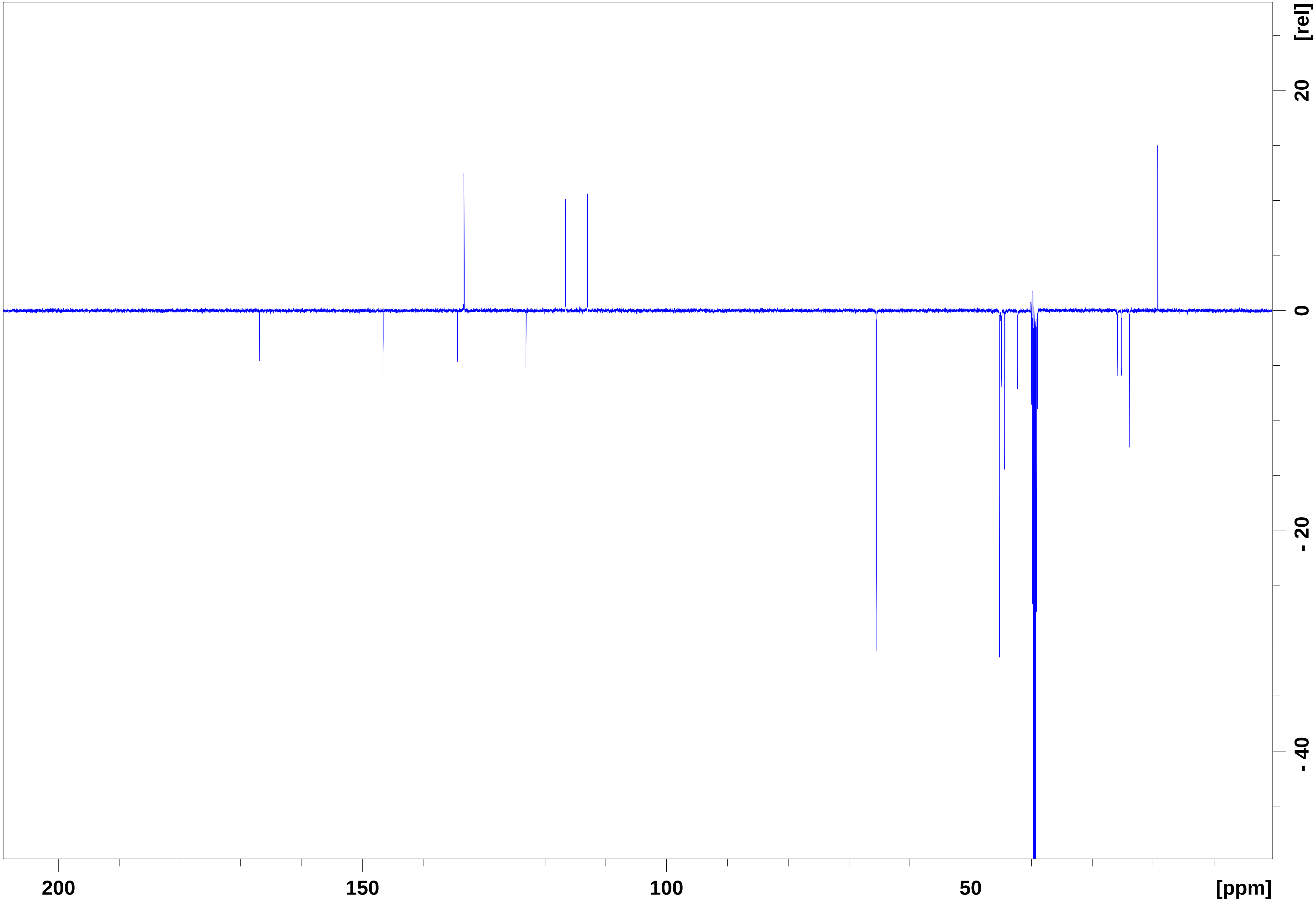

Compound 15

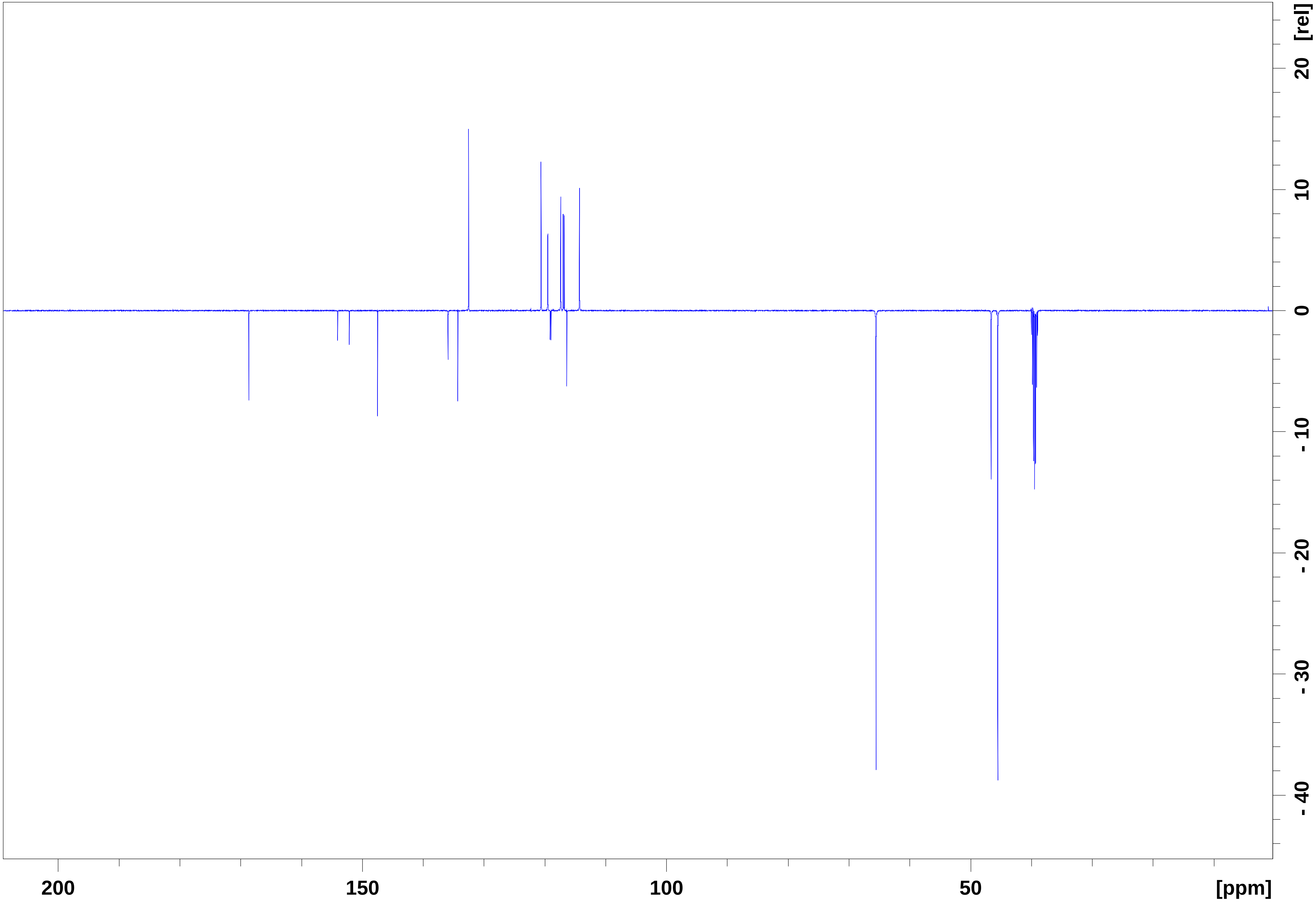

Compound 16

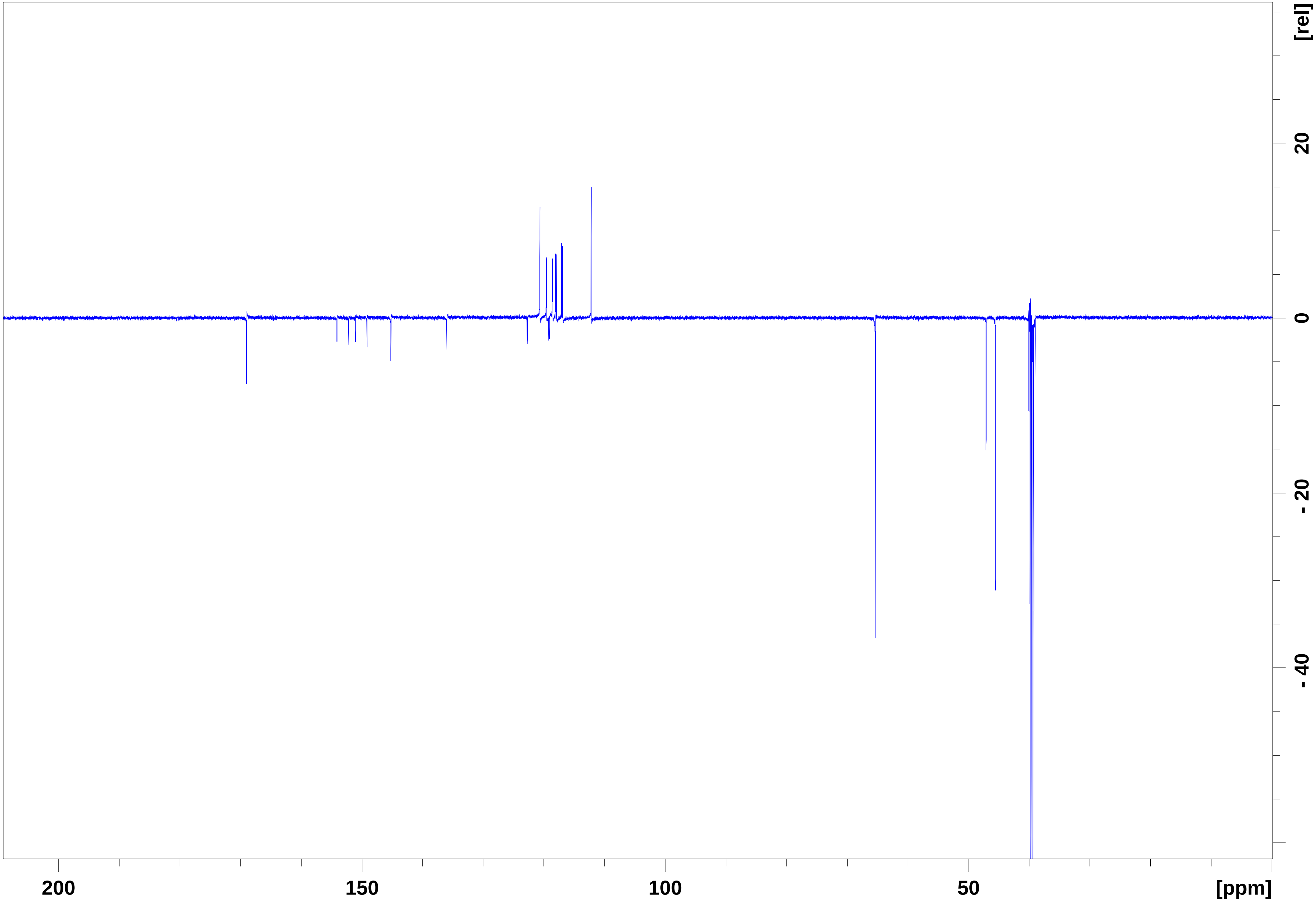

Compound 17

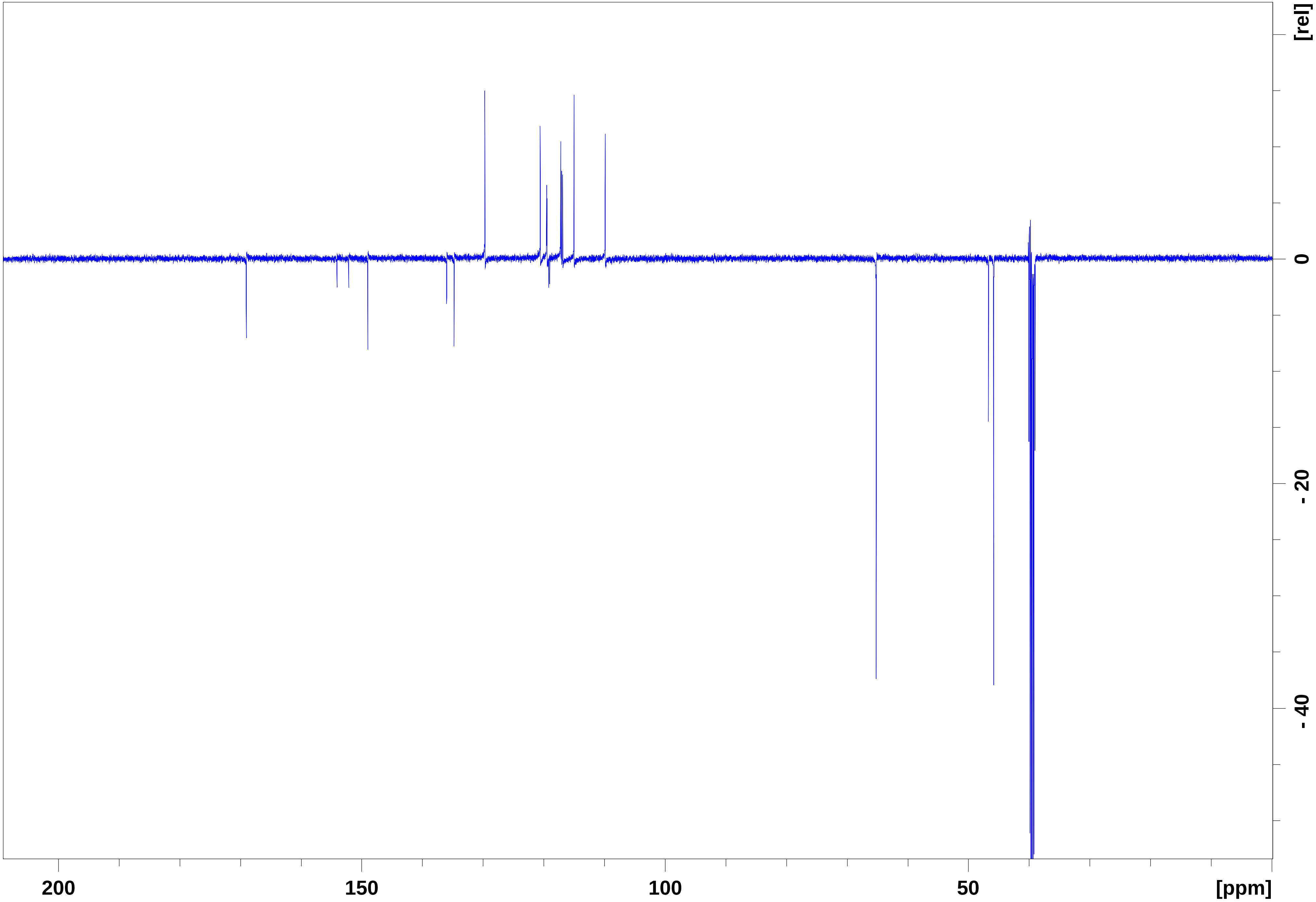

Compound 21

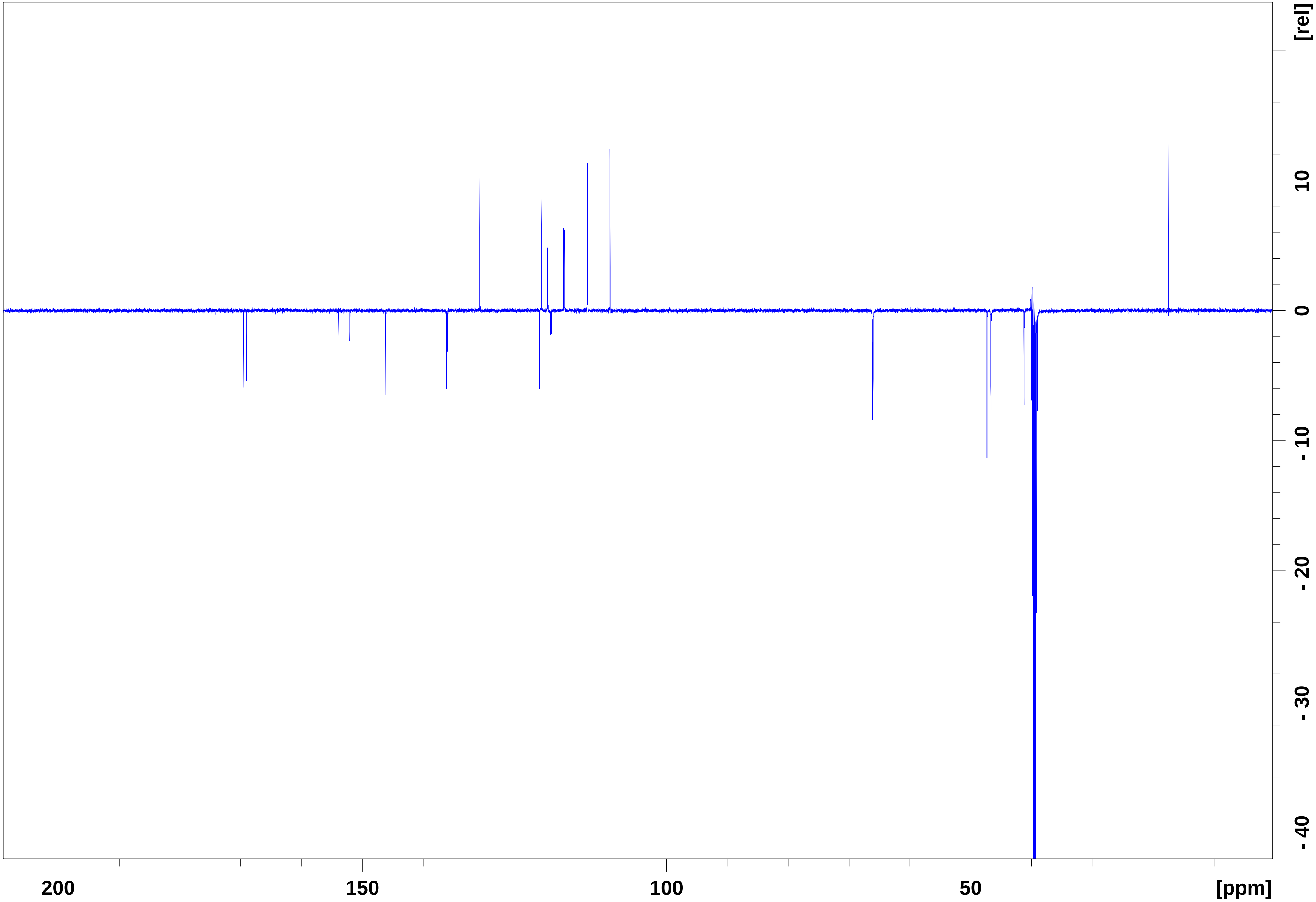

Compound 23

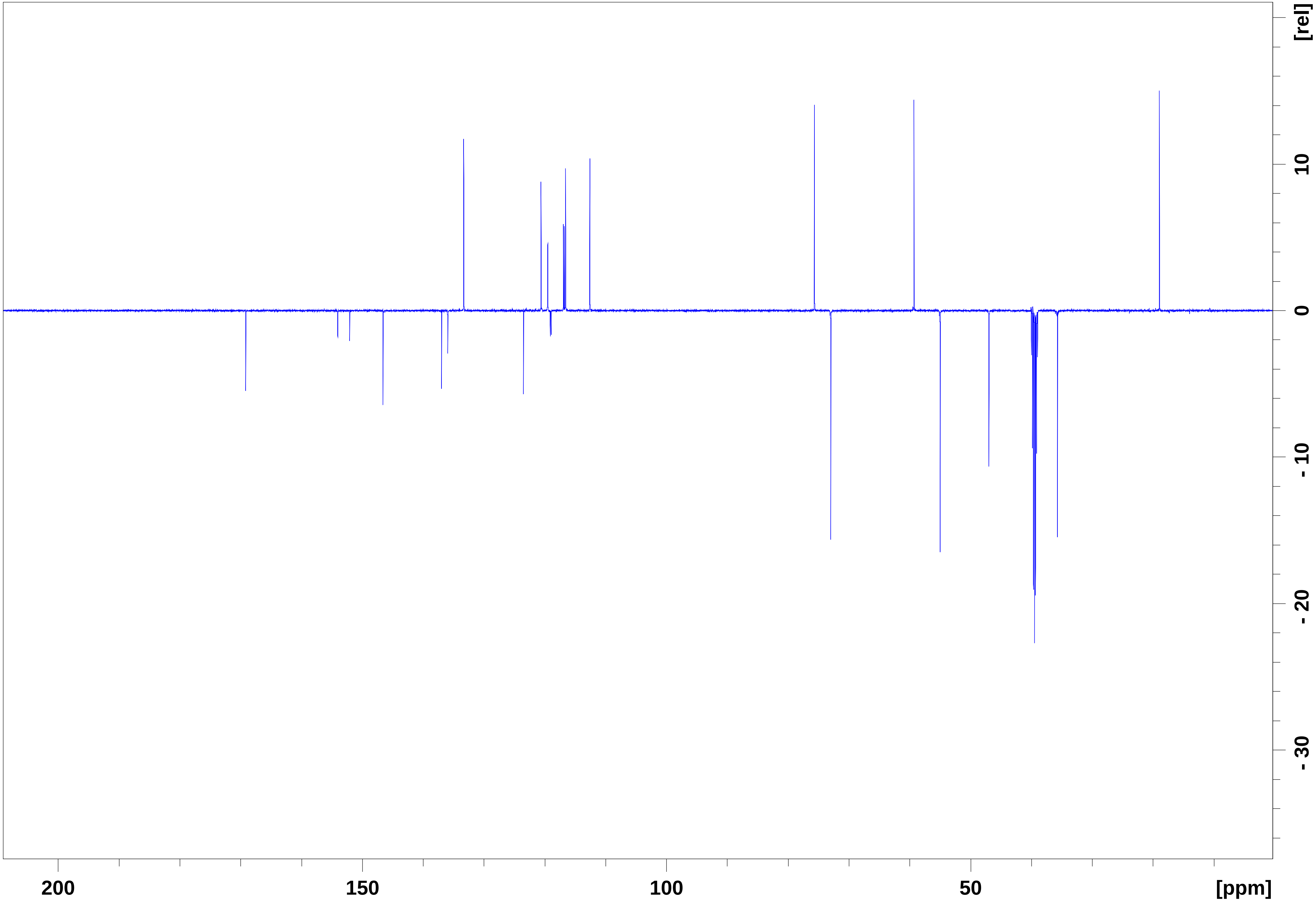

Compound 24

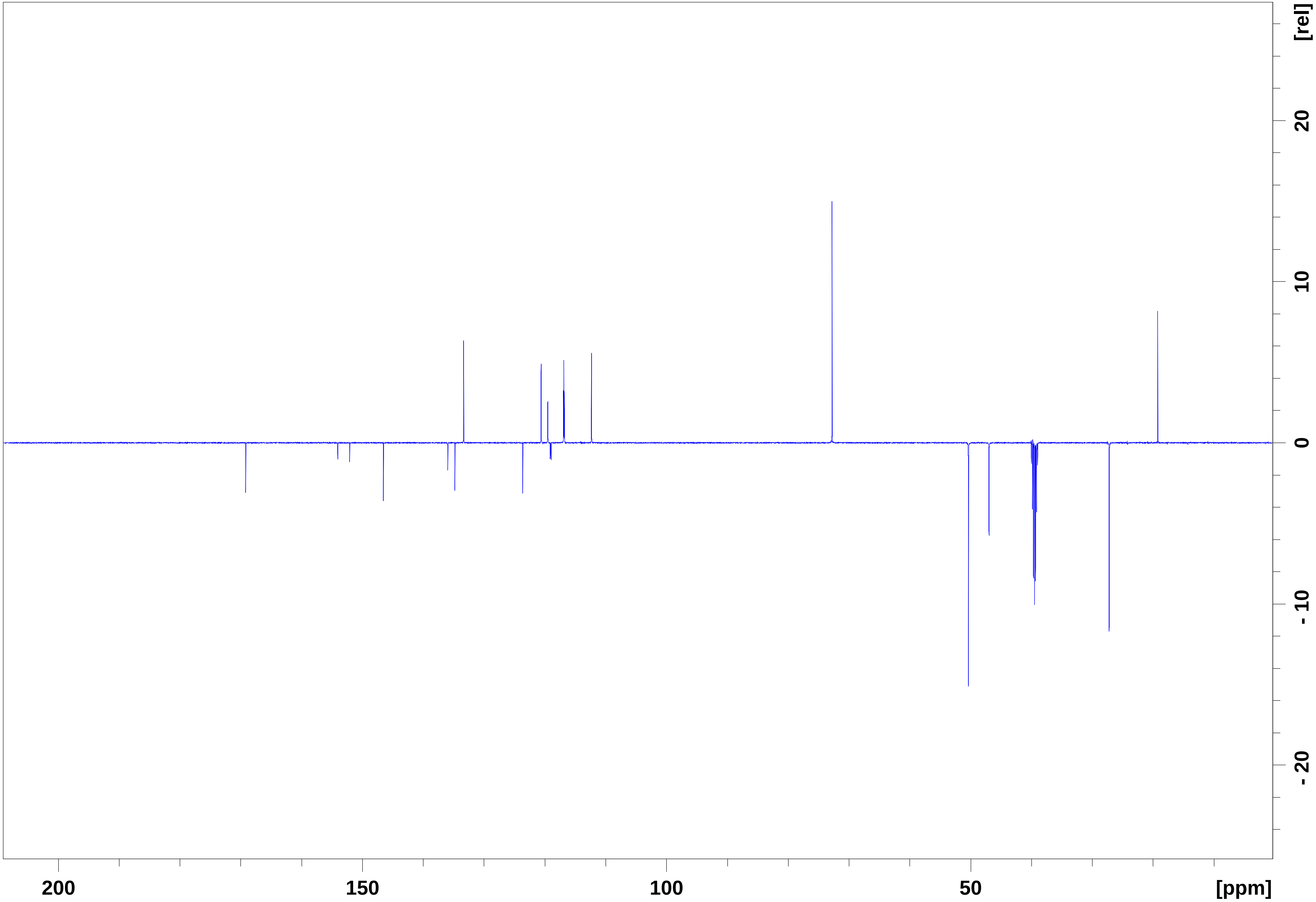

Compound 26

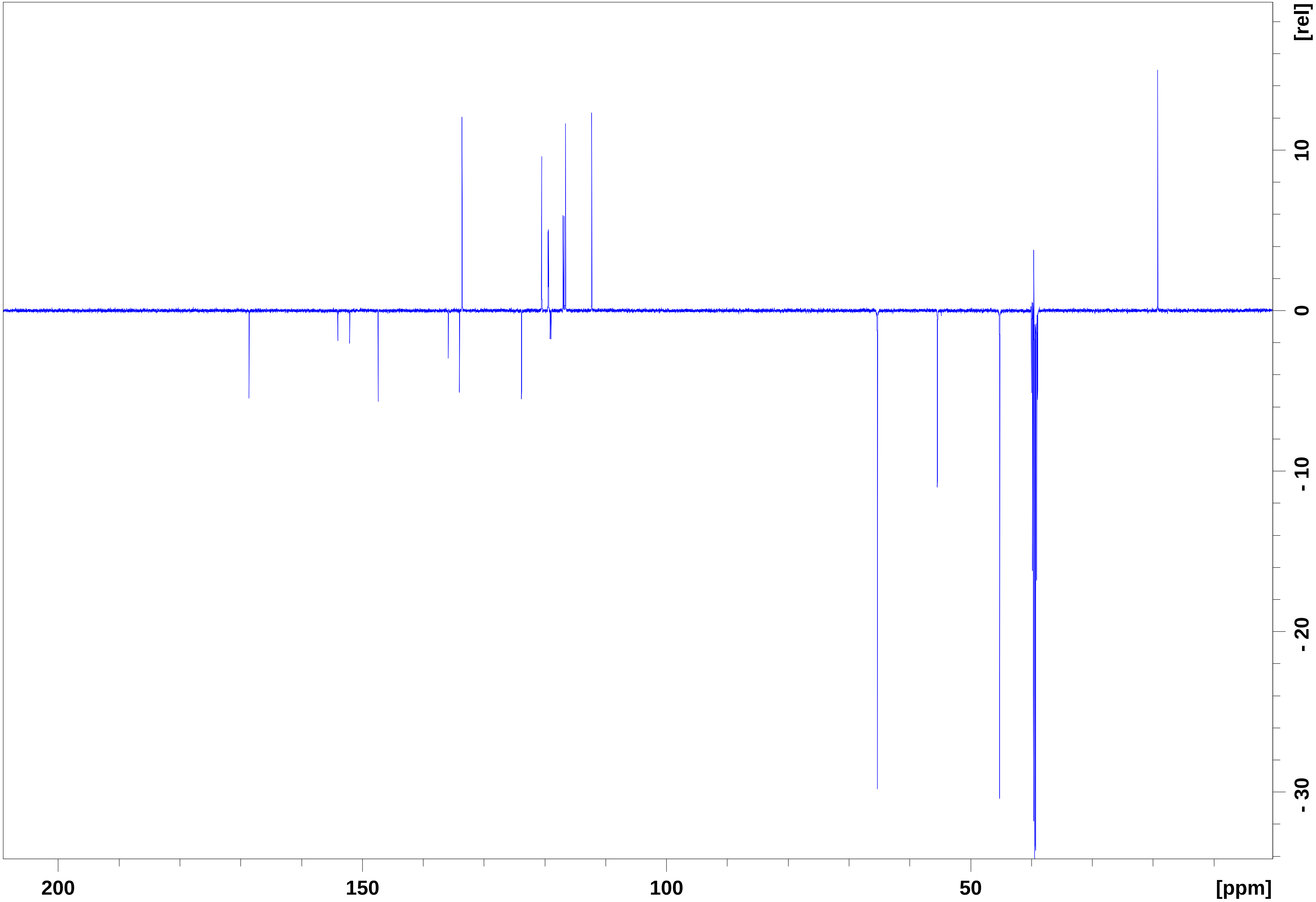

Compound 27

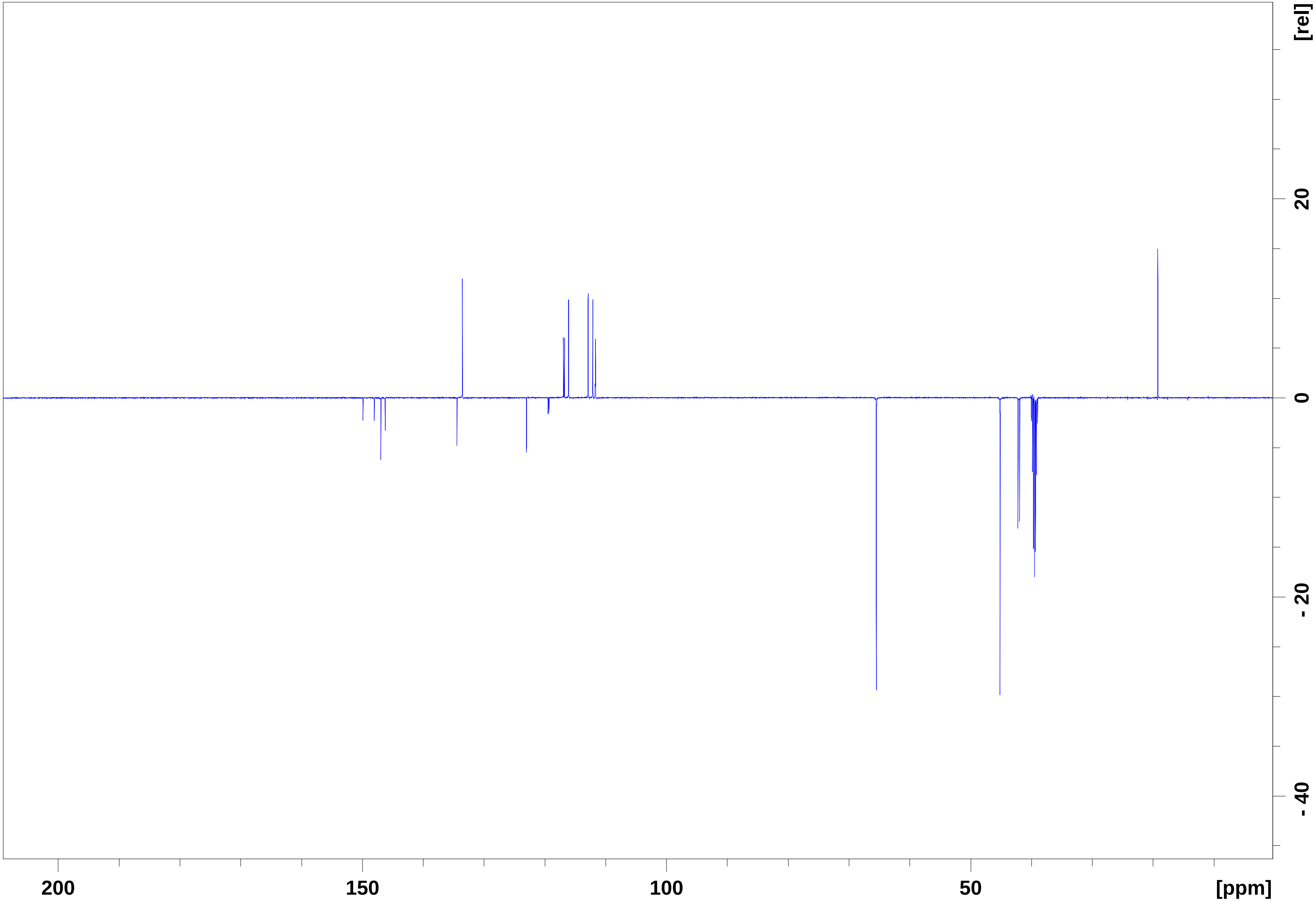

Compound 29

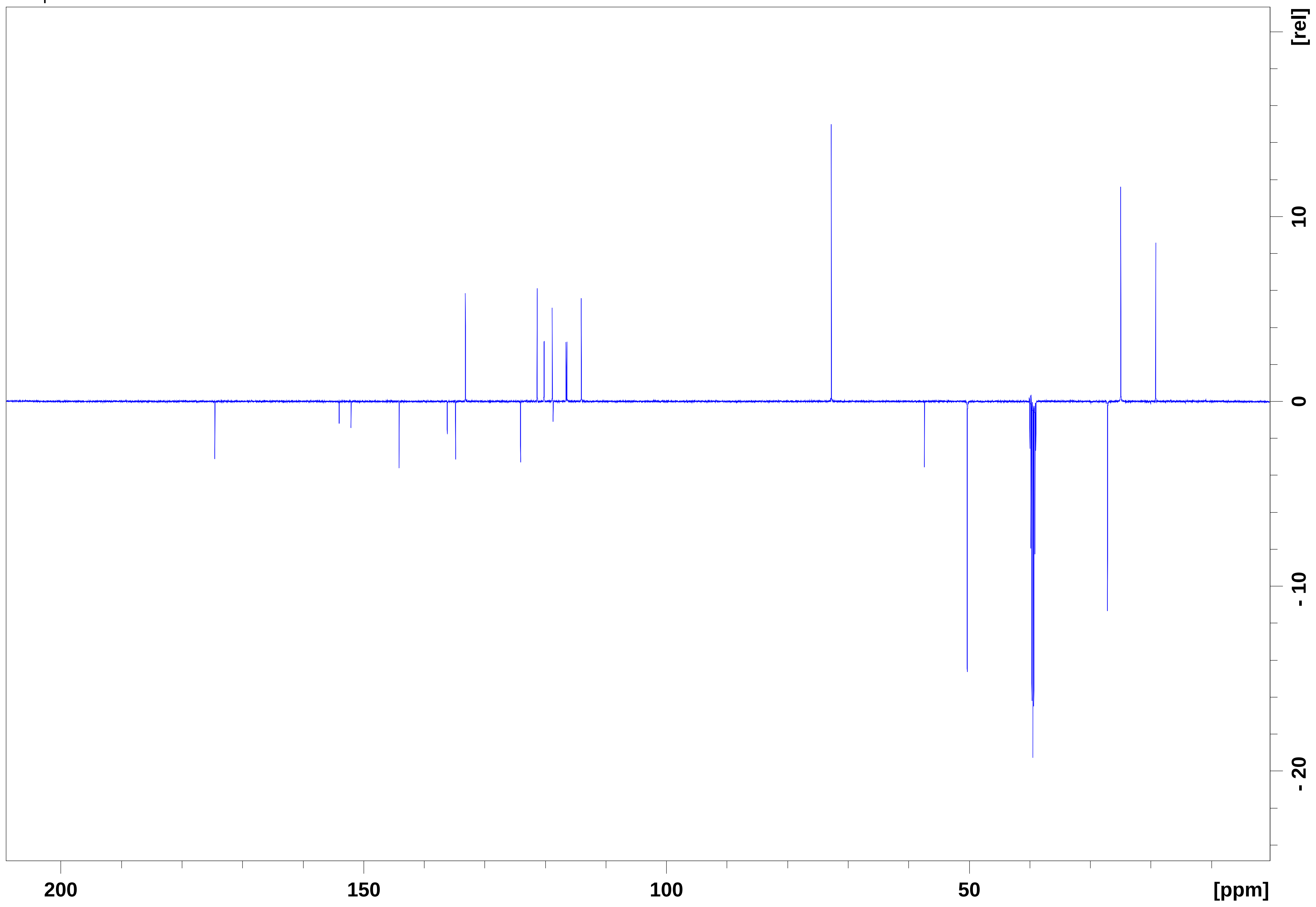

Compound 30

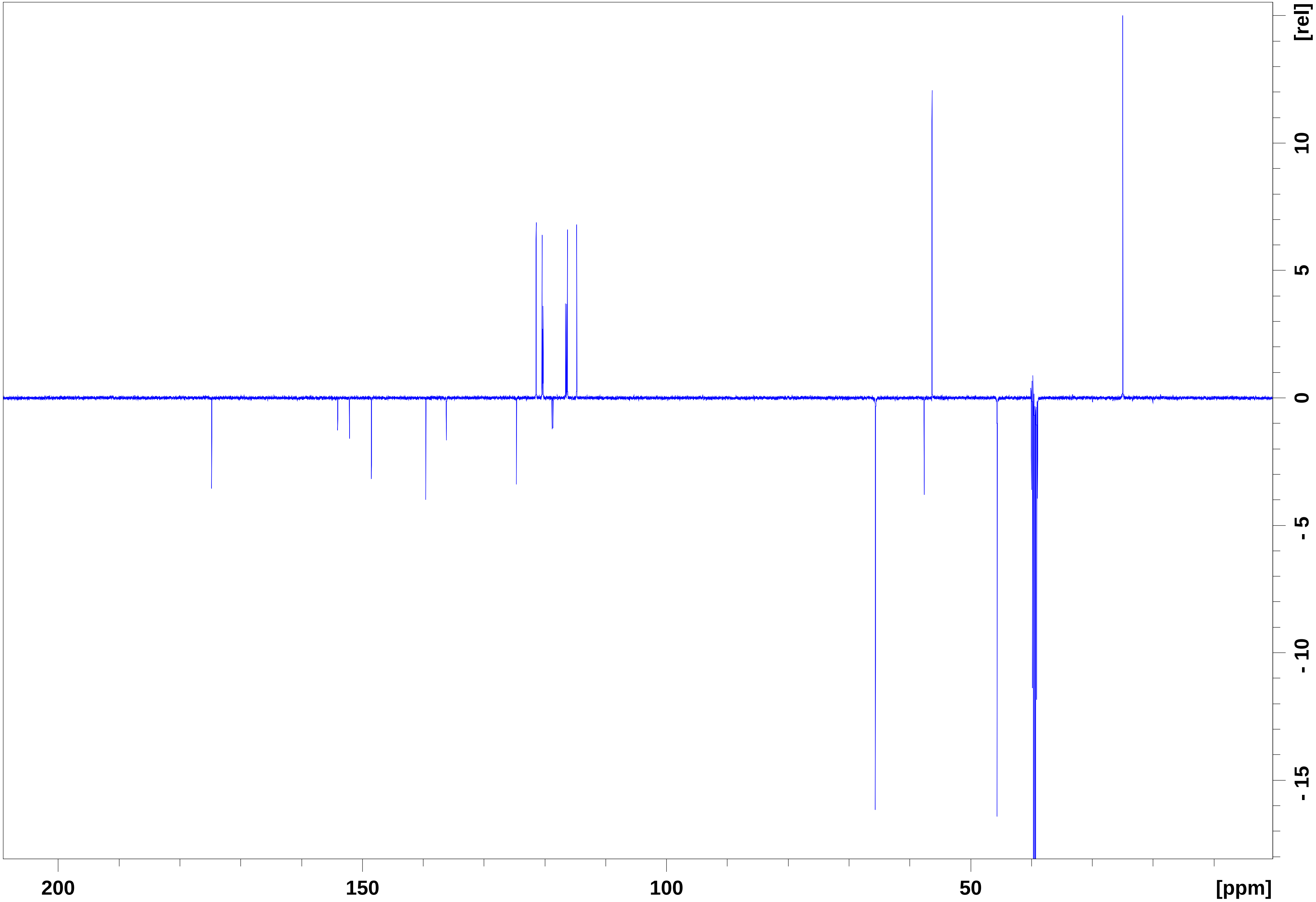

Compound 31

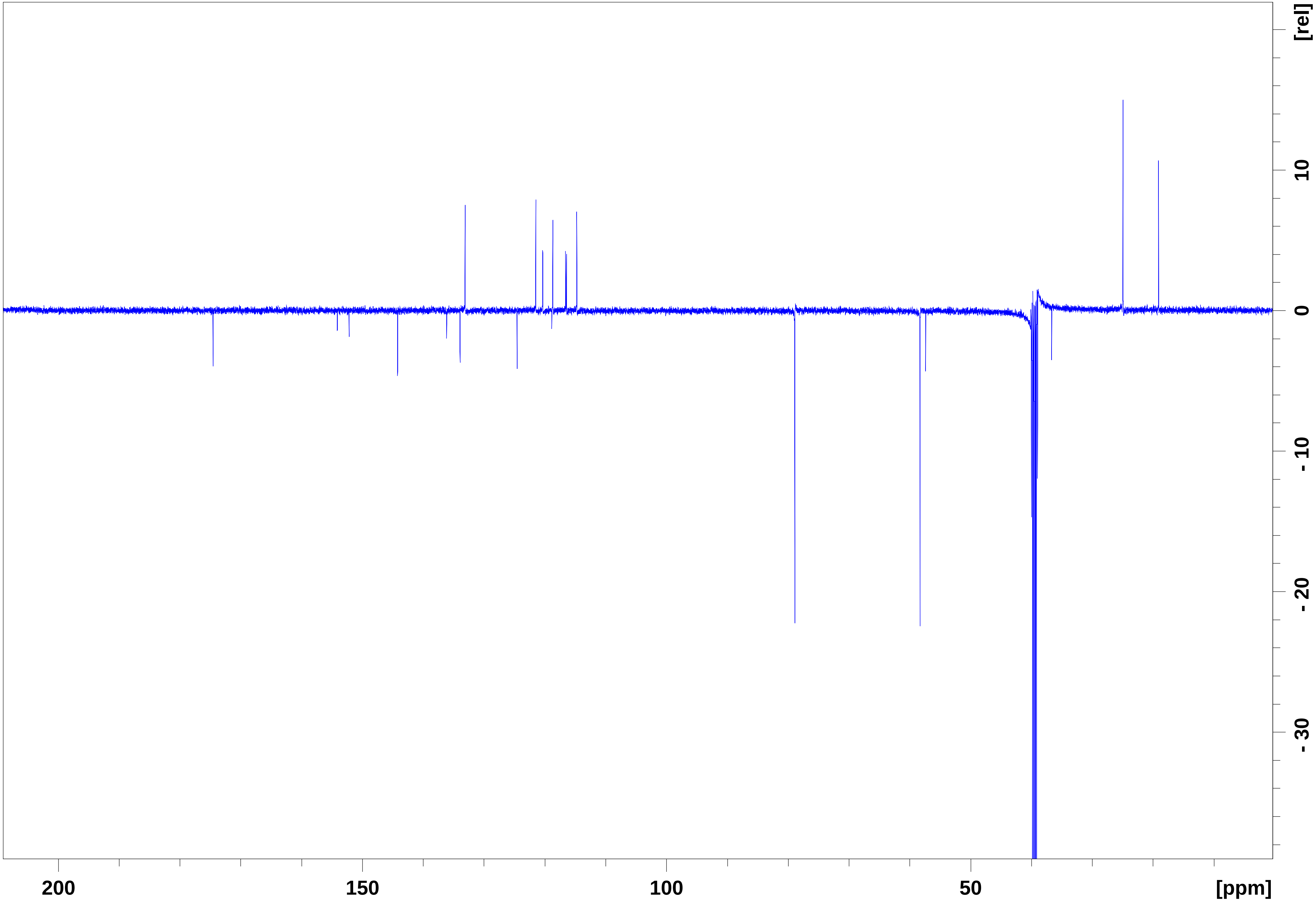

Compound 32

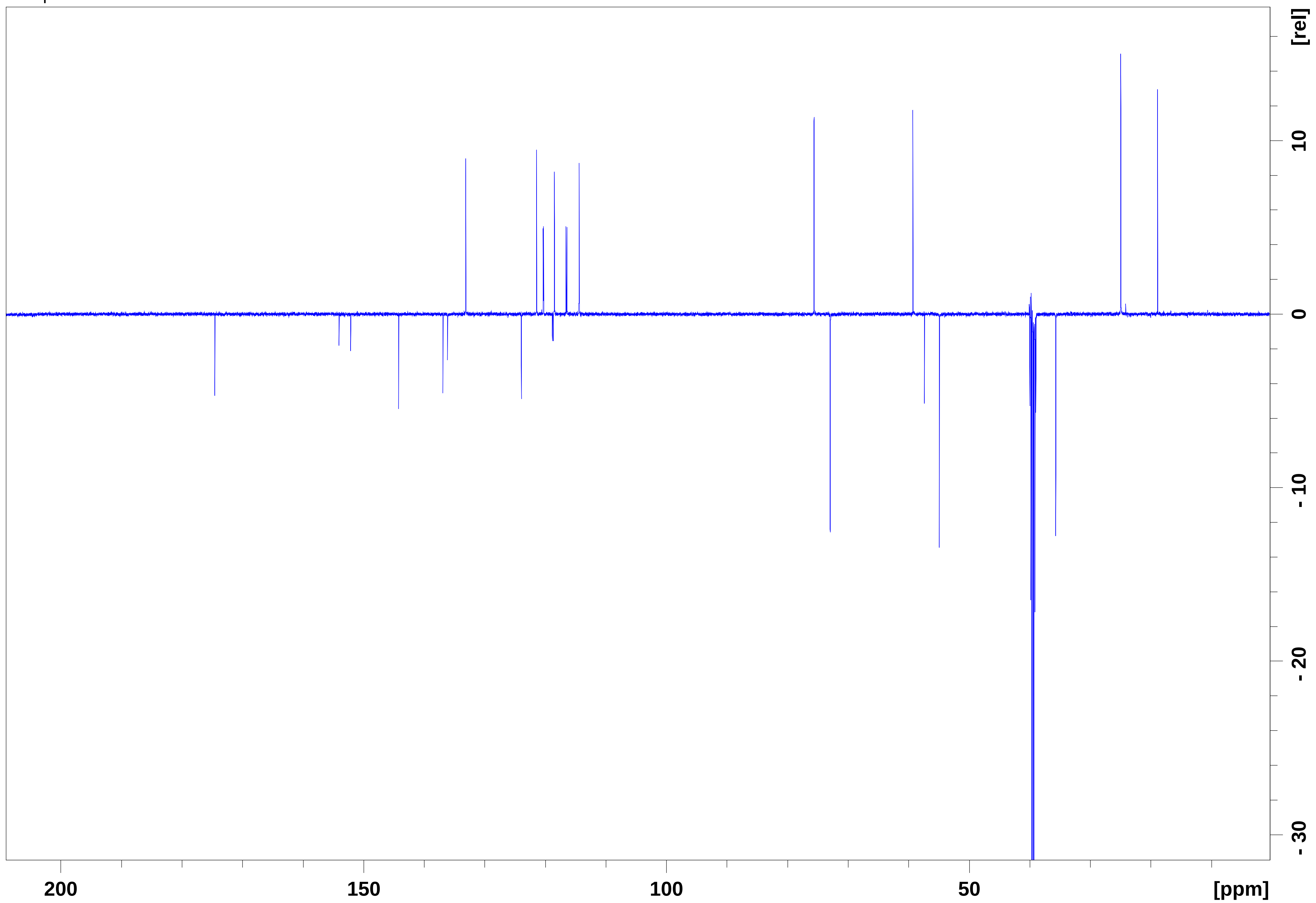

Compound 1

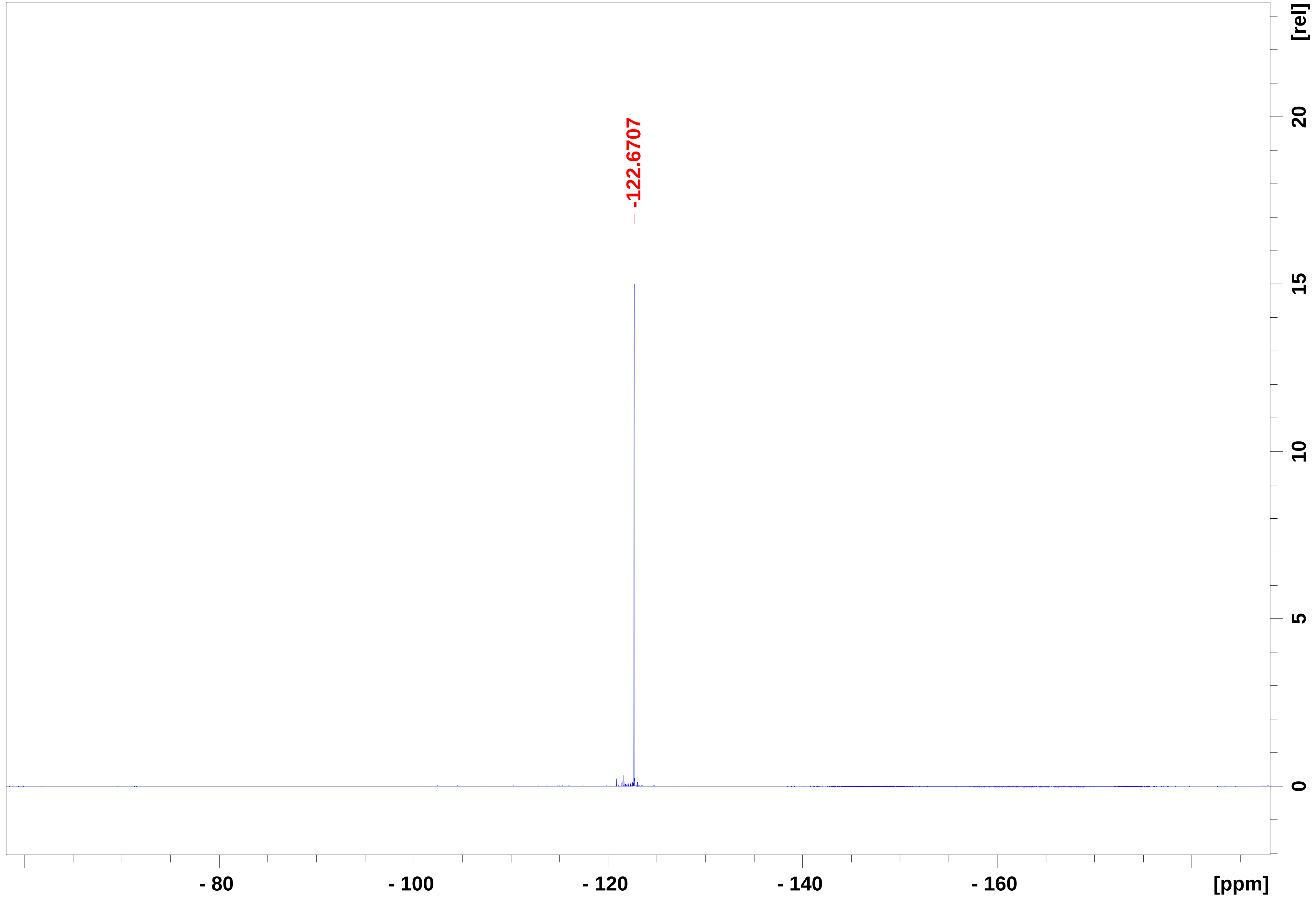

Compound 2

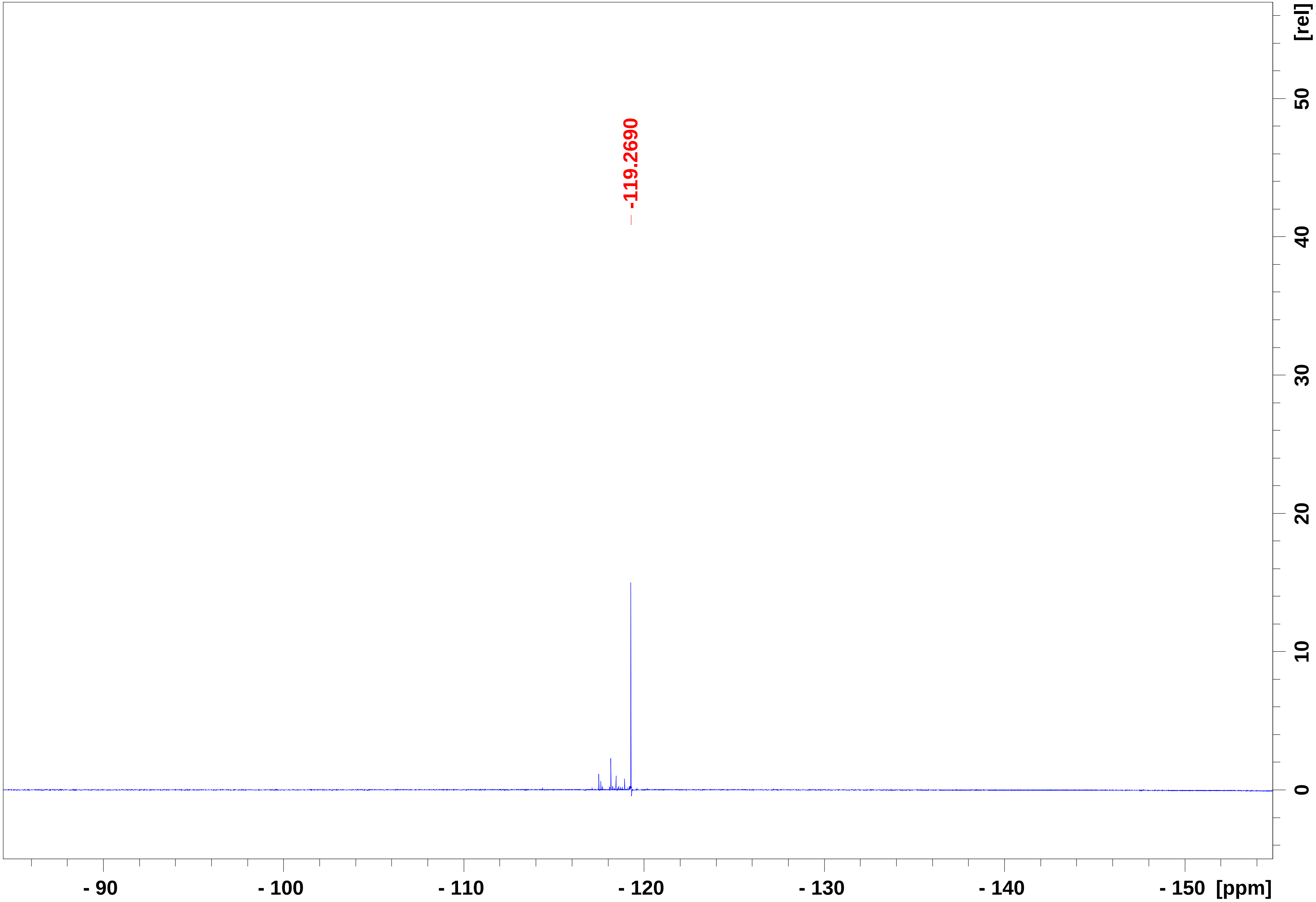

Compound 11

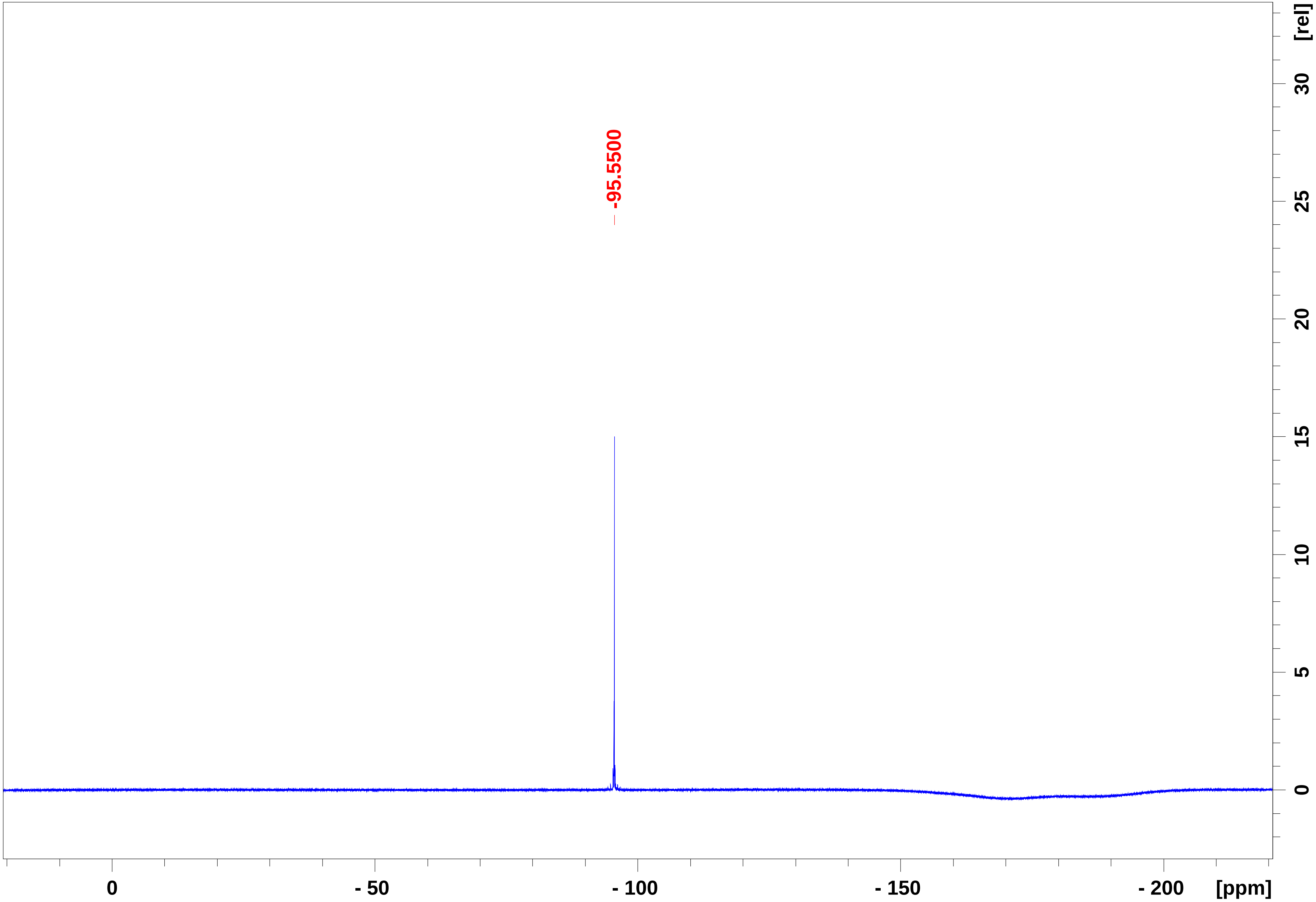

Compound 15

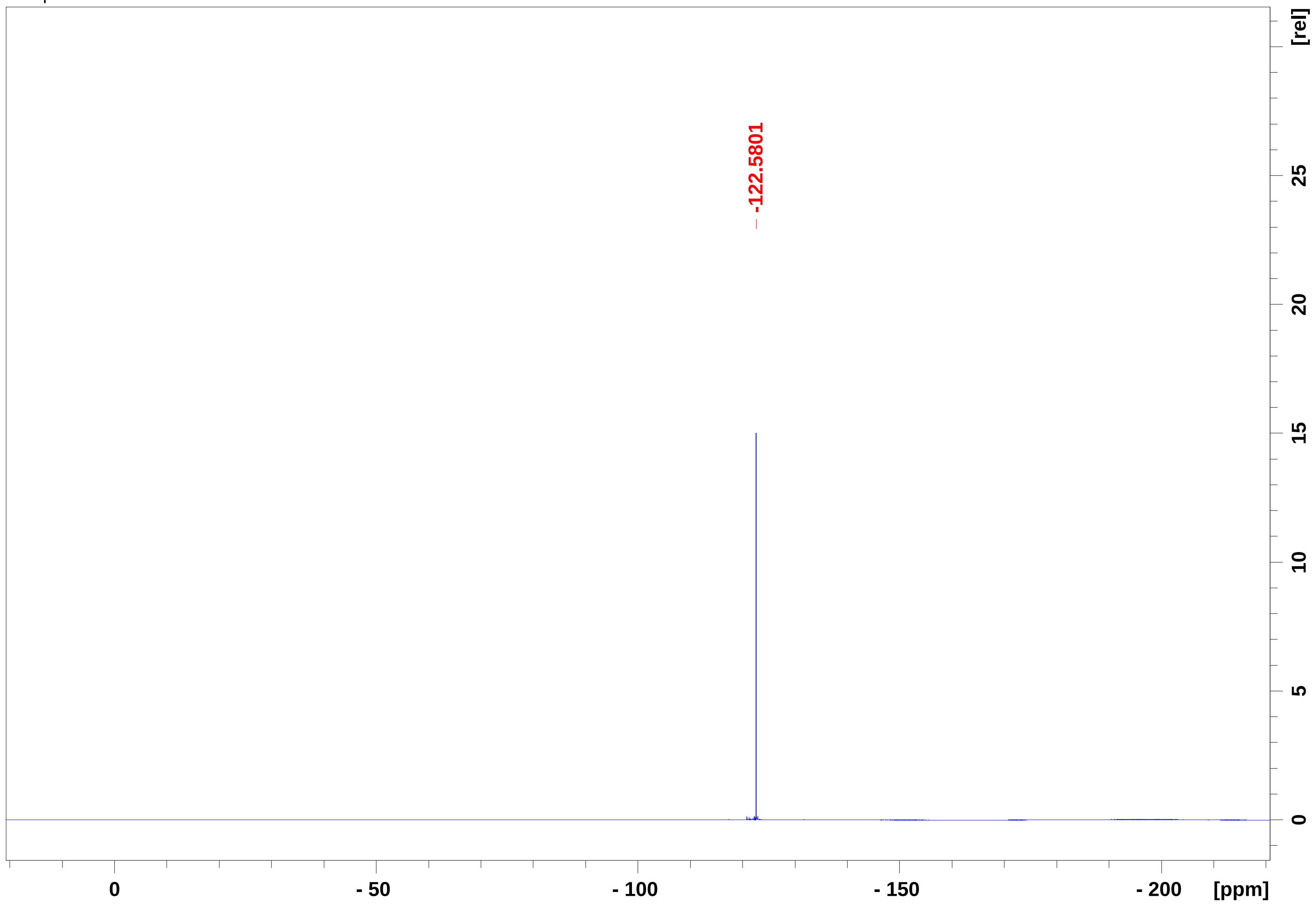

Compound 16

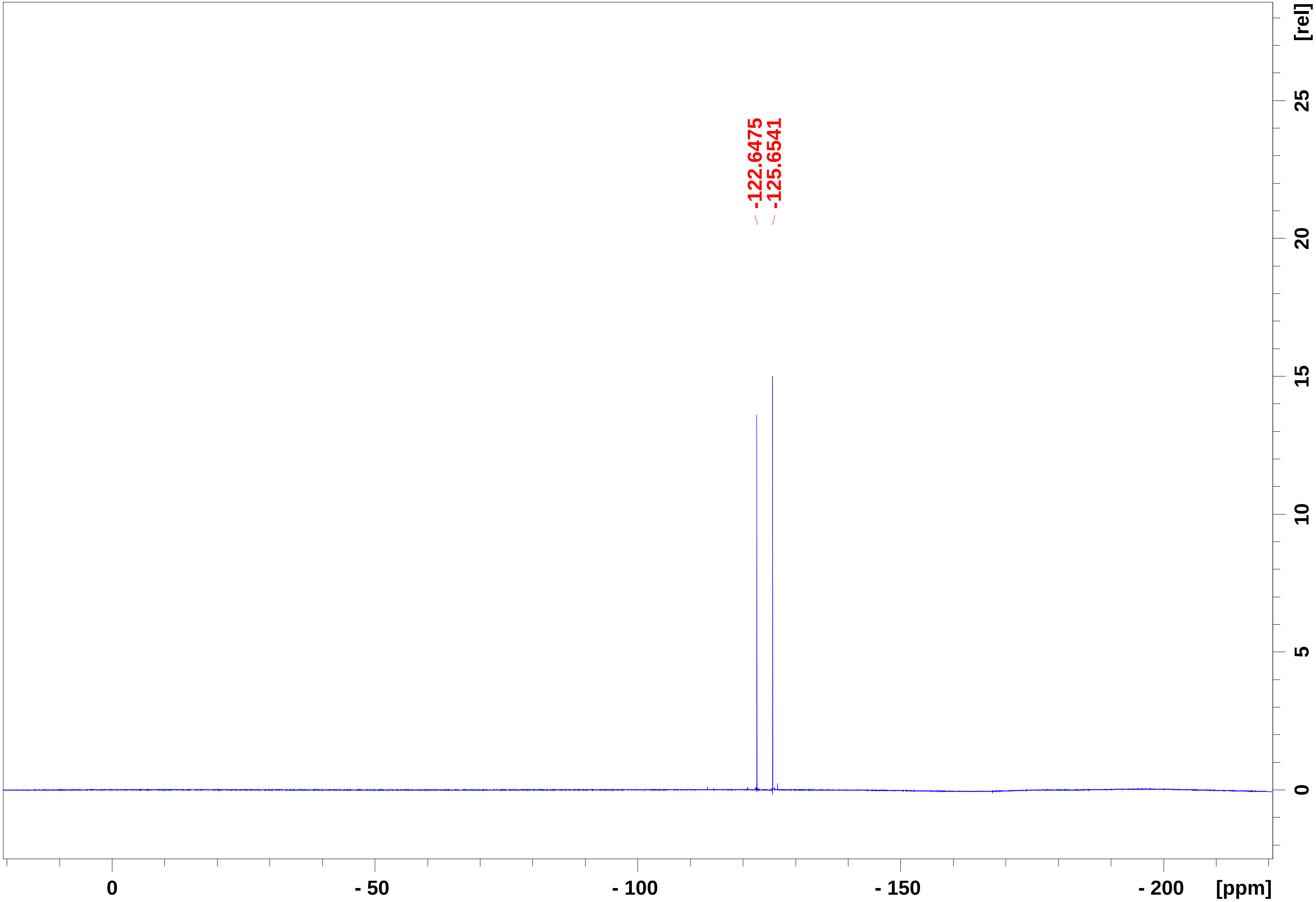

Compound 17

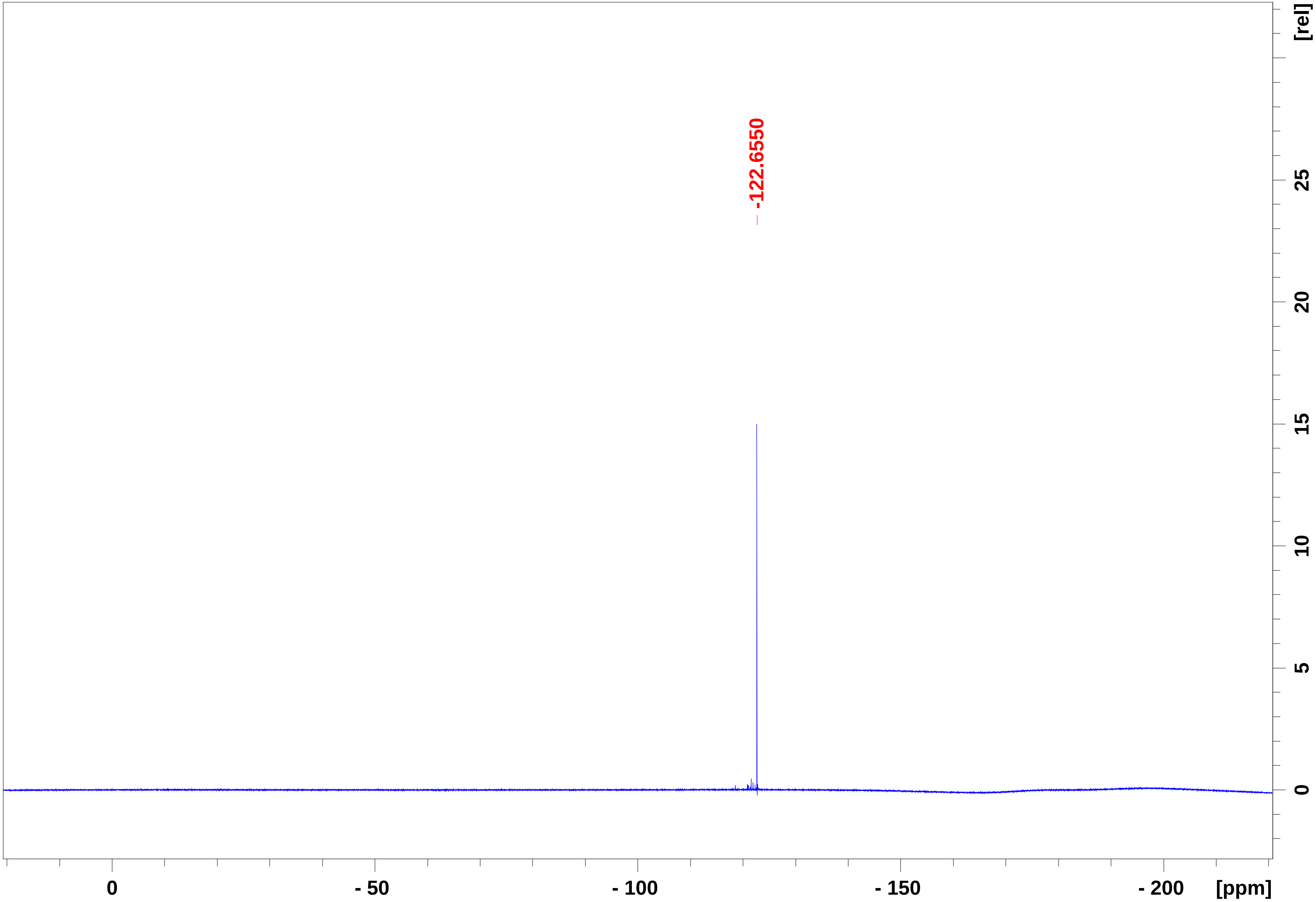

Compound 21

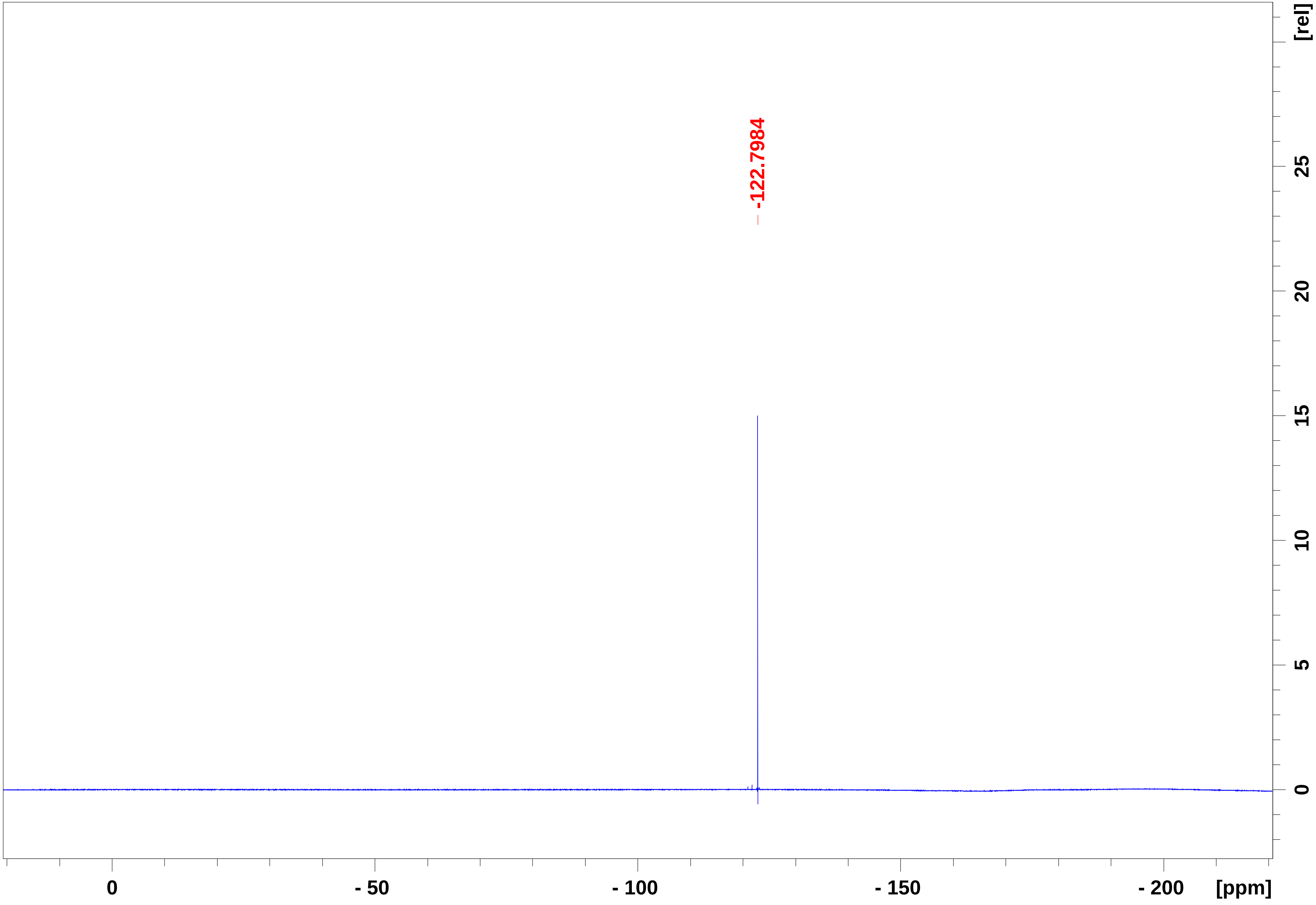

Compound 23

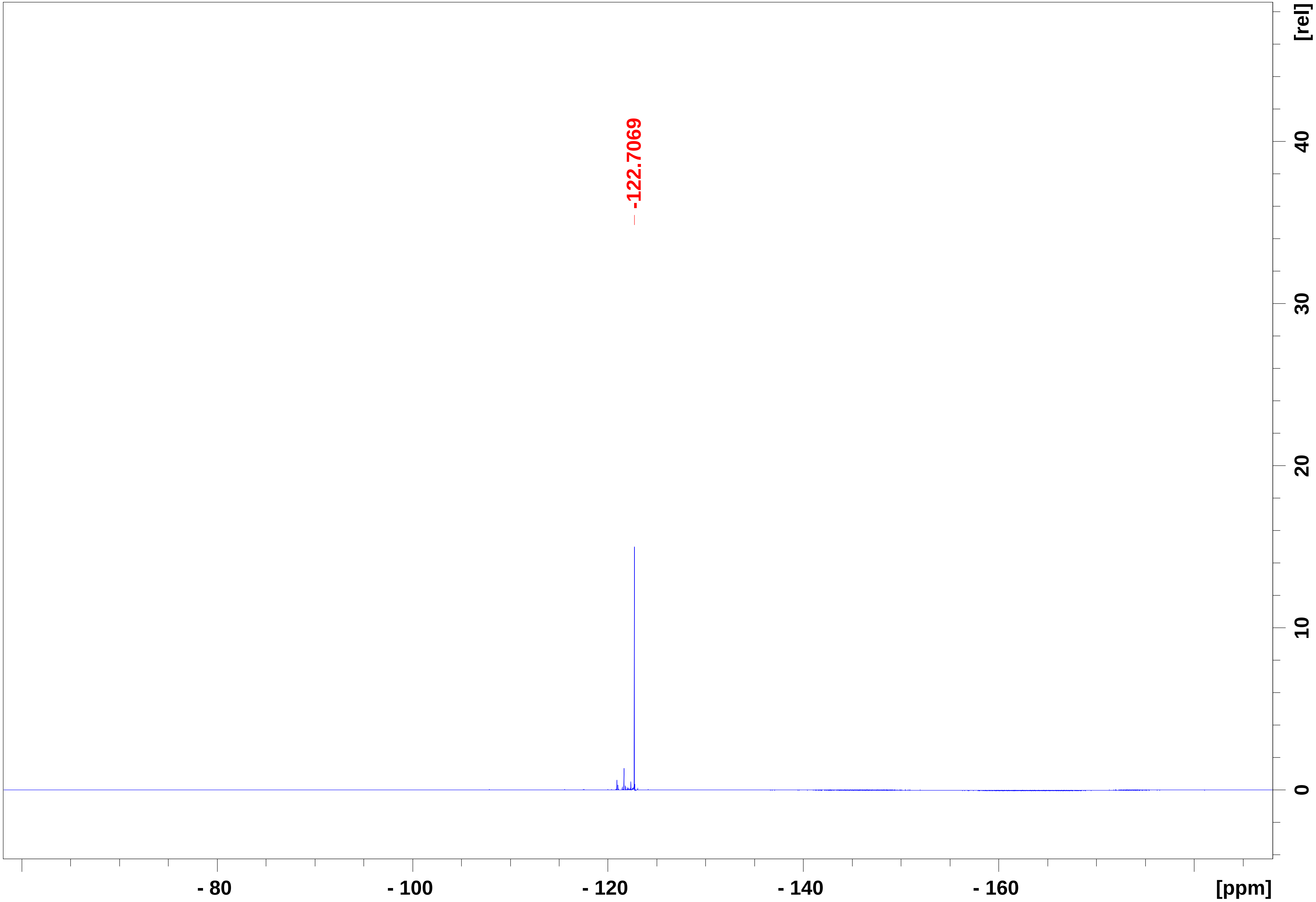

Compound 24

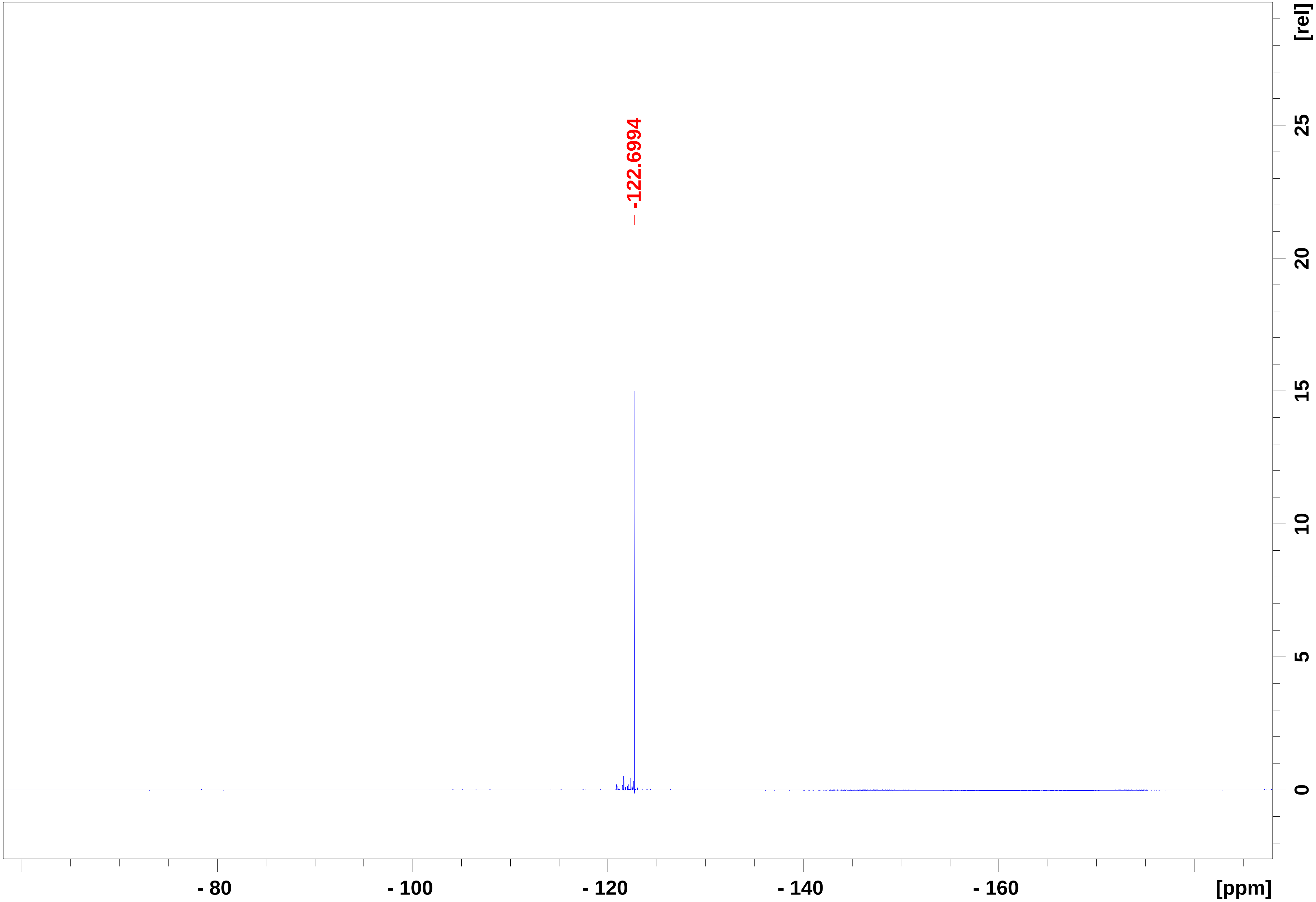

Compound 26

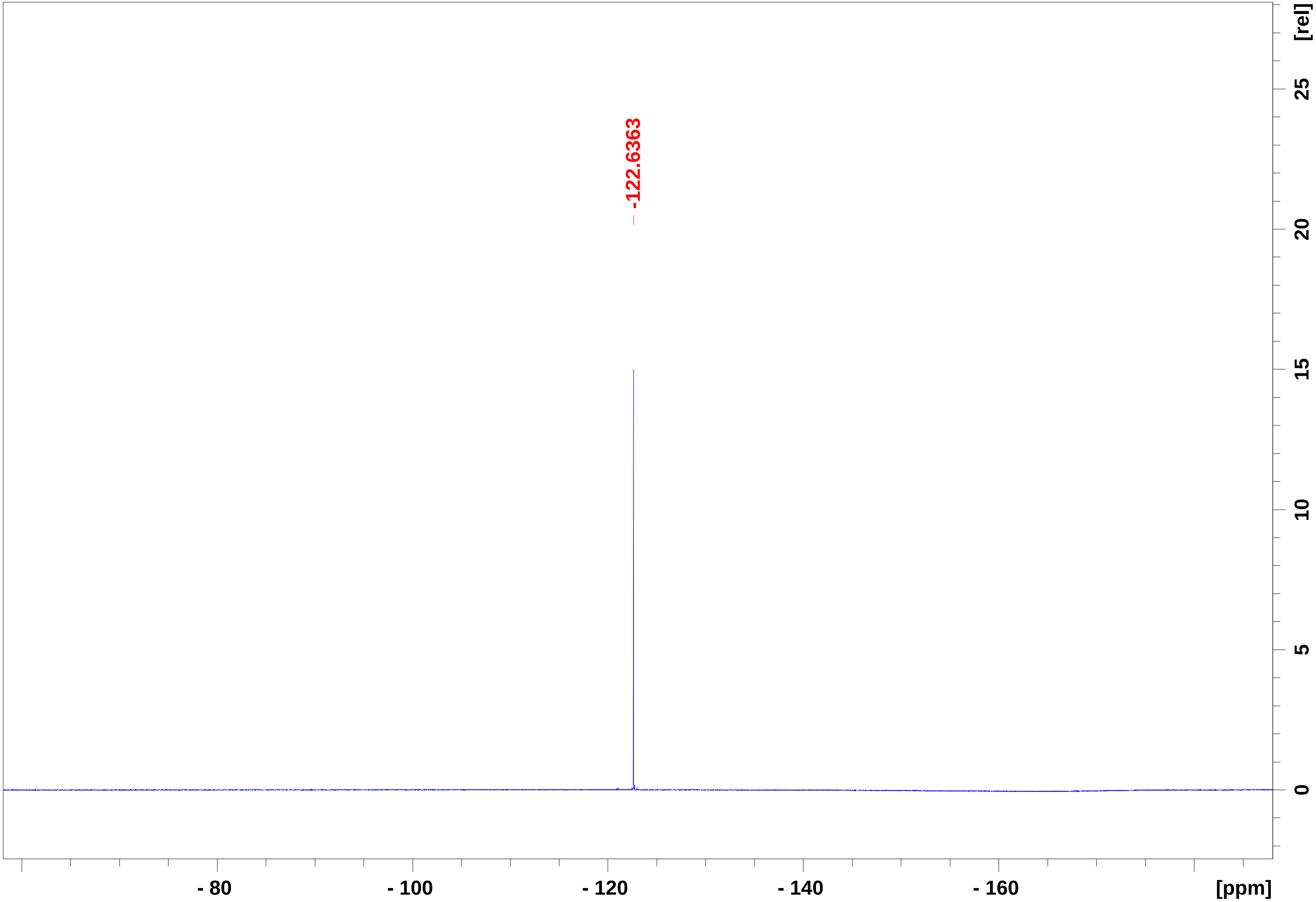

Compound 27

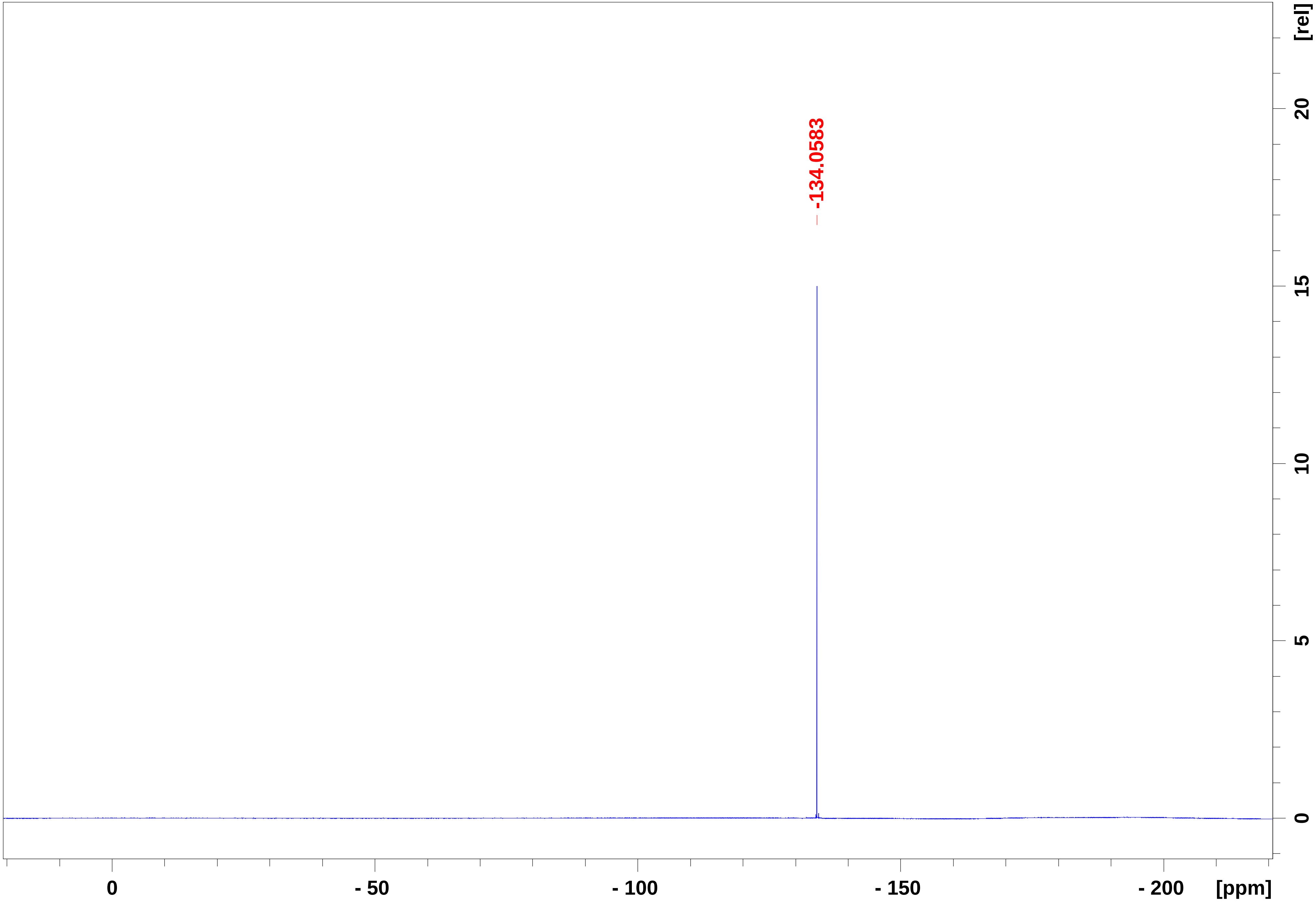

Compound 29

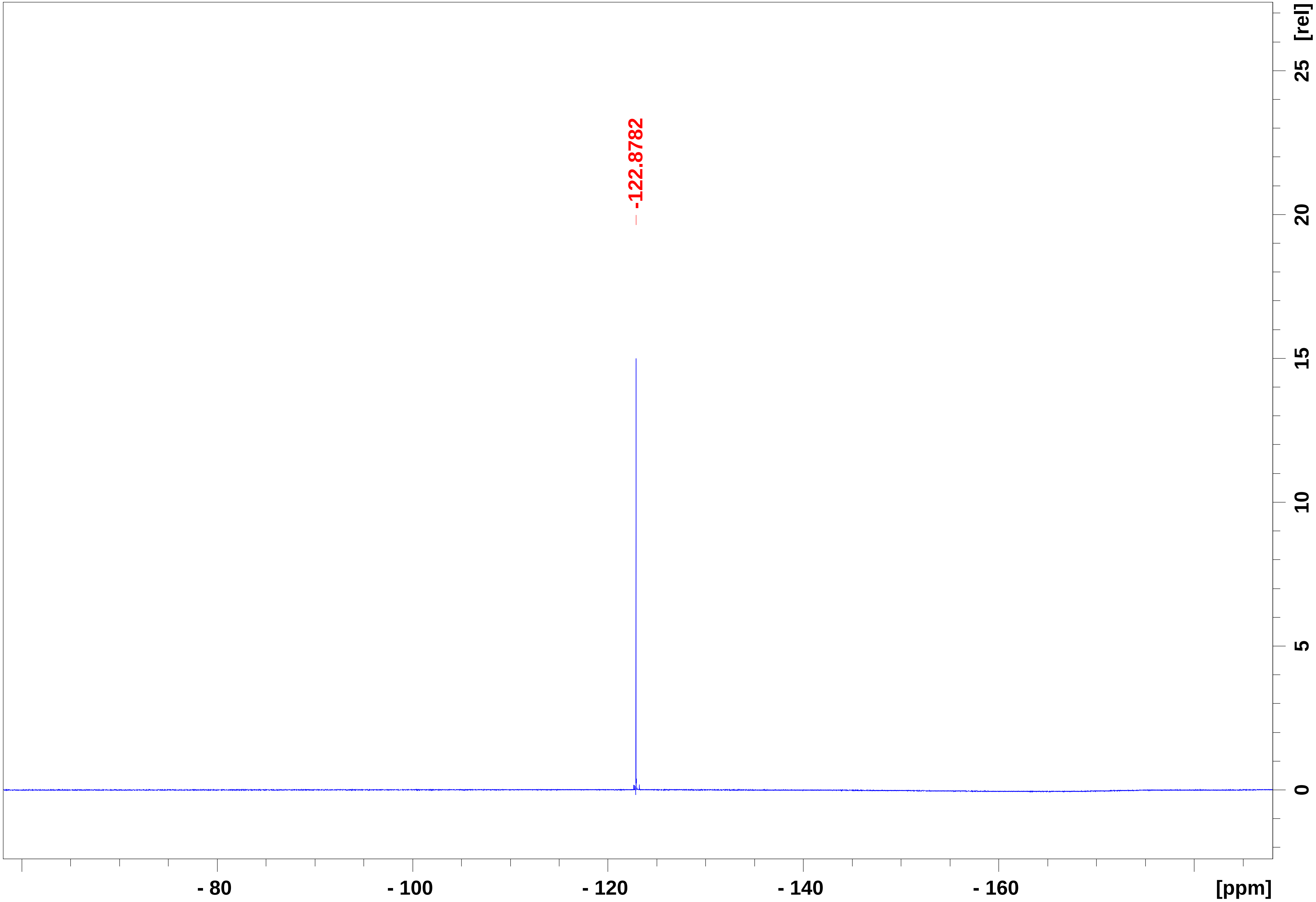

Compound 30

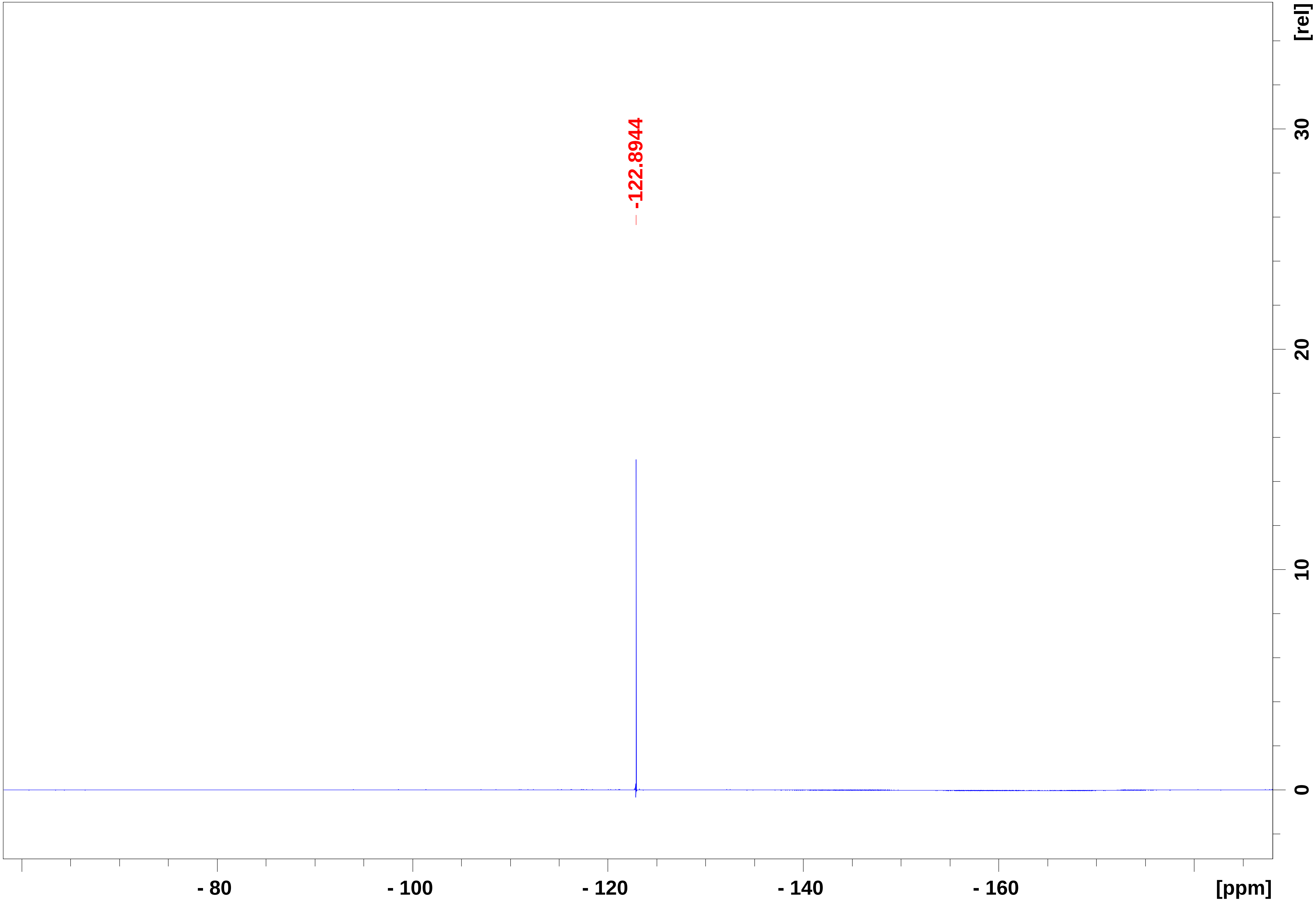

Compound 31

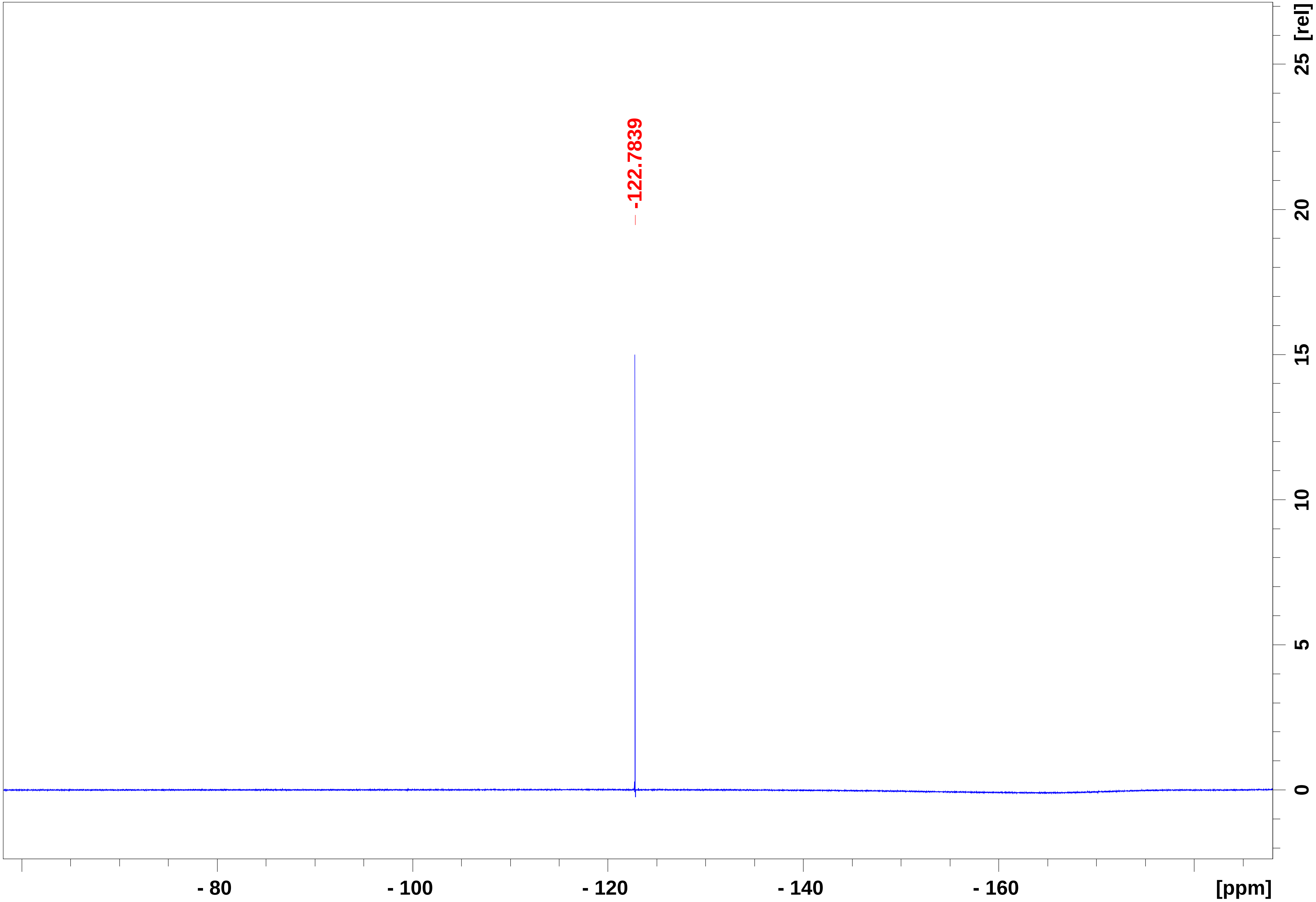

Compound 32

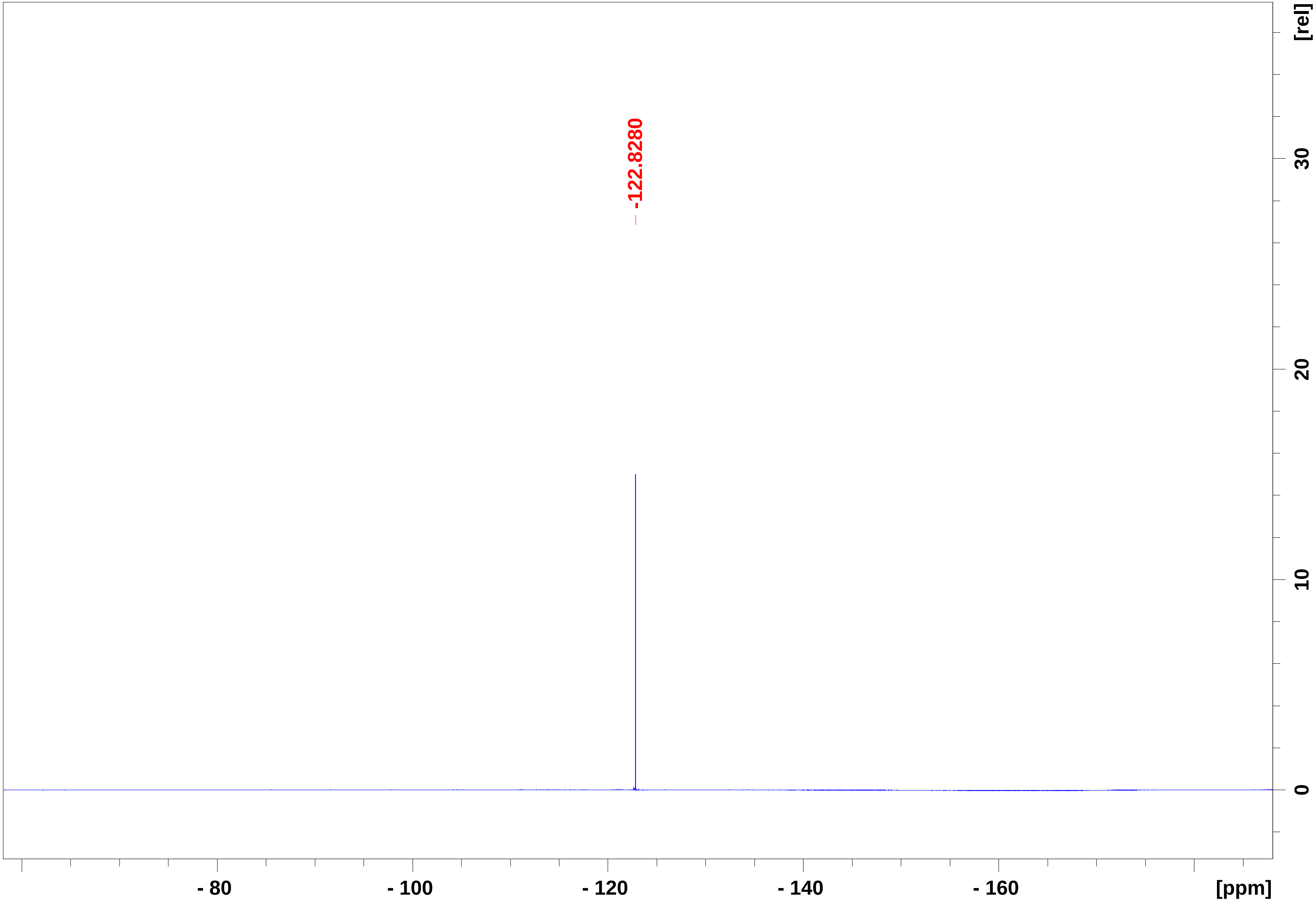

Supplement: Supplementary file 1 — Supplementary [file CMDC-14-1329-s001.pdf]
